# The impact of child health interventions and risk factors on child survival in Kenya, 1993–2014: a Bayesian spatio-temporal analysis with counterfactual scenarios

**DOI:** 10.1186/s12916-021-01974-x

**Published:** 2021-05-04

**Authors:** Peter M. Macharia, Noel K. Joseph, Robert W. Snow, Benn Sartorius, Emelda A. Okiro

**Affiliations:** 1 Population Health Unit, Kenya Medical Research Institute-Wellcome Trust Research Programme, Nairobi, Kenya; 2 Centre for Tropical Medicine and Global Health, Nuffield Department of Medicine, University of Oxford, Oxford, UK; 3 Department of Health Metrics Sciences, School of Medicine, University of Washington, Seattle, WA USA

**Keywords:** Kenya, Sub-national, Counterfactual, Impact, Under-five mortality, Spatio-temporal

## Abstract

**Background:**

During the millennium development goals period, reduction in under-five mortality (U5M) and increases in child health intervention coverage were characterised by sub-national disparities and inequities across Kenya. The contribution of changing risk factors and intervention coverage on the sub-national changes in U5M remains poorly defined.

**Methods:**

Sub-national county-level data on U5M and 43 factors known to be associated with U5M spanning 1993 and 2014 were assembled. Using a Bayesian ecological mixed-effects regression model, the relationships between U5M and significant intervention and infection risk ecological factors were quantified across 47 sub-national counties. The coefficients generated were used within a counterfactual framework to estimate U5M and under-five deaths averted (U5-DA) for every county and year (1993–2014) associated with changes in the coverage of interventions and disease infection prevalence relative to 1993.

**Results:**

Nationally, the stagnation and increase in U5M in the 1990s were associated with rising human immunodeficiency virus (HIV) prevalence and reduced maternal autonomy while improvements after 2006 were associated with a decline in the prevalence of HIV and malaria, increase in access to better sanitation, fever treatment-seeking rates and maternal autonomy. Reduced stunting and increased coverage of early breastfeeding and institutional deliveries were associated with a smaller number of U5-DA compared to other factors while a reduction in high parity and fully immunised children were associated with under-five lives lost. Most of the U5-DA occurred after 2006 and varied spatially across counties. The highest number of U5-DA was recorded in western and coastal Kenya while northern Kenya recorded a lower number of U5-DA than western. Central Kenya had the lowest U5-DA. The deaths averted across the different regions were associated with a unique set of factors.

**Conclusion:**

Contributions of interventions and risk factors to changing U5M vary sub-nationally. This has important implications for targeting future interventions within decentralised health systems such as those operated in Kenya. Targeting specific factors where U5M has been high and intervention coverage poor would lead to the highest likelihood of sub-national attainment of sustainable development goal (SDG) 3.2 on U5M in Kenya.

## Background

During the millennium development goals (MDGs) era, all-cause under-five mortality (U5M) reduced in Kenya but was characterised by spatio-temporal disparities and inequities [1]. MDG 4 target—to reduce U5M by two-thirds between 1990 and 2015—was achieved neither at national nor at sub-national level [1] with the current sustainable development goal (SDG) 3.2 aiming to reduce U5M to a more optimistic target of less than 25 deaths per 1000 live births by 2030 [2]. Similarly, improvements were observed in the coverage of interventions and declines in disease infection prevalence, which also showed spatial and temporal heterogeneities across the country [3–8]. However, the impact of sub-national intervention coverage, distribution of resources, healthcare utilisation and disease infection on the spatio-temporal disparities observed in U5M has not been adequately defined [9–13]. This limits application of sub-nationally targeted interventions for public health planning and attainment of child health development goals.

The differences observed in the rates of U5M change between counties in Kenya dictate an improved understanding of the competing risk factors and intervention contributions to child survival. Focusing on interventions with the highest impact will facilitate improved targeted disease control, better resource allocation, focus on equity and maximising impact [9, 14–16]. Therefore, policymakers will tailor U5M reducing policies in line with the SDGs principle of *leaving no one behind and reaching the farthest behind, first* by covering those most marginalised [17, 18]. In Kenya, such analyses focusing on inequities in child health have previously been conducted [4–6, 19–24].

However, the previous analyses [4–6, 19–24] relied on factors referring to the time of survey while U5M refers to a retrospective period resulting in temporal mismatches. The studies have often reported risk ratios and overlooked the prevalence of exposure. Where the population that is exposed has been accounted for, the analyses either are at the national level, use a limited set of factors, or are based on a single or limited time epoch and none has considered the impact of a full range of factors in the continuum of child survival at the units of decision-making [4–6, 19–24].

To address these limitations and data gaps, here, we collate all available data within spatio-temporal and counterfactual models to quantify how much reductions in U5M were associated with scale-up of interventions and changes in disease prevalence between 1993 to 2014 at 47 decentralised counties in Kenya (Additional file 1) to improve sub-national health planning and facilitate a reduction of health inequities.

## Methods

### Approach overview

Our analysis involved four main steps. First, data on U5M rates and factors associated with child survival were synthesised and estimated at the county level from population censuses and household sample surveys [1]. Second, a set of parsimonious factors significantly associated with U5M in the Kenyan context were selected. In the third step, a Bayesian ecological space–time mixed-effects regression model was fitted to quantify the relationship between the parsimonious set of factors and U5M. In the fourth step, counterfactual analysis, an approach widely used to assess causal attribution [25–34] in health applications [33–38], was used to determine how much changes in U5M (deaths averted (U5-DA) or lives lost (U5-LL)) were attributable to the changes in intervention coverage and disease infection prevalence between 1993 and 2014.

### Data

The outcome variable was U5M available for each county across 22 years from 1993 to 2014 generated using demographic and spatio-temporal models detailed elsewhere [1]. In brief, ten household surveys and three population censuses with birth histories were assembled and spatially aligned to county boundaries. Five demographic methods were applied to estimate U5M per county by survey and smoothed using a Bayesian spatio-temporal Gaussian process regression (GPR) accounting for spatio-temporal relatedness, sample size and demographic methods [1].

The candidate list of predictor variables included 43 factors known to be associated with U5M (Additional file 1) available at county level spanning across 22 years (Table 1 and Additional file 1). The 43 factors were identified from existing frameworks of child survival [39–42] (Additional file 1), relevance to Kenya’s health priorities and data availability and defined based on household survey guidelines while ensuring temporal comparability. Estimates for 39 factors were generated using data from 20 household surveys and three population censuses via a Bayesian spatio-temporal GPR model while four factors were available from disparate sources [7, 43–45]. Table 1 outlines the factors, while detailed definitions and the specific data sources for each factor are presented in Additional file 1. Our analyses included data up to 2014 when the last household sample survey was conducted. Alternative data sources are limited; the coverage and completeness of data from civil registration and vital statistics systems remains low in Kenya while routine data does not capture those who do not interact with the health systems and misses majority of deaths that happen in the communities.

**Table 1:** Forty-three factors associated with child survival and thematic groups as used in the current analysis. Their definitions and respective data sources are detailed in Additional file 1

Group	ID	Variable
Environmental factors	1	Rural residency
	2	Precipitation
	3	Enhanced vegetation index (EVI)
Maternal factors	4	Maternal education
	5	Maternal literacy
	6	Female-headed households (maternal autonomy)
	7	Short birth spacing
	8	Use of modern contraceptives
	9	High parity
Child factors	10	Underweight
	11	Wasted
	12	Stunted
	13	Breastfed within the first hour of birth
	14	Exclusive breastfeeding
	15	Continued breastfeeding
	16	Low birth weight (LBW)
Household factors	17	Poor household
	18	Improved sanitation
	19	Access to any form of a toilet
	10	Improved water
	21	Access to wells borehole and piped water
Infections	22	HIV infection prevalence
	23	Malaria infection prevalence
Healthcare utilisation	24	At least one antenatal care visit (ANC1)
	25	At least four antenatal care visits (ANC4)
	26	Skilled birth attendance (SBA)
	27	Health facility deliveries (HFD)
	28	Diarrhoea treatment-seeking
	29	Fever/cough treatment-seeking
Child health interventions	30	Bacille Calmette–Guérin (BCG)
	31	Three diphtheria–tetanus–pertussis vaccinations (DPT3)
	32	Three doses of polio (Polio3)
	33	Measles
	34	Fully immunised
	35	Oral rehydration salts (ORS use)
	36	Vitamin A-children
	37	Insecticide-treated bed nets (ITNs) use by children
	38	Recommended antimalarial use
Maternal health interventions	39	Tetanus toxoid injection
	40	Intermittent preventive treatment in pregnancy (IPTp 1)
	41	IPTp 2
	42	Iron supplement
	43	Vitamin A-mothers

### Statistical analysis

#### Model development

Model development aimed to select a parsimonious set of factors strongly associated with U5M in Kenya to reduce overfitting and fluctuating regression coefficients [46, 47]. Before formal statistical model development, factors whose contribution was captured by other factors among the 43 were excluded to reduce any potential collinearity, circularity and confounding [3, 48, 49] (Additional file 2). The role of insecticide-treated bed nets (ITNs) and antimalarial medicine is captured by malaria prevalence [7, 50, 51]; intermittent preventive treatment in pregnancy (IPTp) role is partially captured by low birth weight (LBW) [52–55] and was available in 13 malaria-endemic counties only [56] while the effect of three doses of diphtheria–tetanus–pertussis (DTP3) vaccine, polio (Polio3), measles and BCG vaccines was captured by fully immunised status. Likewise, factors measuring the same intervention and have an overlapping impact (for example, maternal education and maternal literacy) were grouped, their relationship with U5M quantified via a simple regression and the best fitting factor included based on a lower Akaike Information Criterion (AIC) [3, 48, 49] (Additional file 2).

All the factors under consideration are associated with U5M (Additional file 2). To validate the findings against the Kenyan context, a simple regression model was fitted to explore the bivariate association between U5M, and factors retained in the preceding stage. Factors with a *p*-value < 0.2 were retained and were considered in the elastic net regression (ENR) model. ENR, a rigorous penalisation regression, was used to reduce dimensionality by selecting factors that explain most of the variation in U5M [57–60] as applied in child survival studies [61, 62]. It was implemented via the *glmnet* R statistical package [60] with factors having non-zero coefficients forming the base model. Further simplification of the base model was explored through the Deviance Information Criterion (DIC) and model predictive capability using out of sample validation [46, 47, 63]. The presence of multicollinearity was assessed using the variance inflation factor (VIF) with a cut-off of four with the collinear and interpretable factors combined through principal component analysis [64–66].

#### Mixed-effects regression model

The final list of factors from the model development phase was included in a Bayesian ecological space–time mixed-effects regression model to estimate adjusted association with U5M (Eq. 1). The model included an intercept, fixed effects, spatial and temporal random effects and a space–time interaction term. The random variables were assigned prior distributions that borrowed the strength of information across space and time to capture better the underlying structure of U5M, changes in time-varying factors that affect all counties and unchanging factors of U5M within each county [33, 36].

Equation 1 Bayesian ecological space–time mixed-effects regression for quantifying the association between U5M and factors from the model development process.

$$ \ln {\left(5\mathrm{q}0\right)}_{i,t}={\beta}_o+{\beta}_j\sum \limits_{j=1}^n{x}_j+{\mu}_i+{v}_i+{\gamma}_t+{\delta}_{i,t} $$

where ln(5q0)_*i*, *t*_ is the natural logarithm of U5M in county *i* and year *t*, *β*_*o*_ the intercept, *β*_*j*_ regression coefficients for the fixed effects, $\sum \limits_{j=1}^n{x}_j$, (*μ*_*i*_) the structured spatial random effect, *v*_*i*_ the unstructured spatial random effect, (*γ*_*t*_) the temporal random effect and *δ*_*i*, *t*_ the type 1 space–time interaction specified for parsimony and concerns on identifiability with highly structured interactions.

The spatial dependence was defined by a neighbourhood matrix through the queen adjacency with the value of a parameter in one county influenced by the average value of its neighbouring counties with some additional variability. The convolution Besag, York and Mollié (BYM) conditional autoregressive (CAR) model was used to express spatial dependence [67, 68]. Similarly, temporal neighbours were defined by the adjacent period points (preceding and post) while the space–time interaction parameter *δ*_*i*, *t*_ accounted for any departure from predictable patterns based on the overall temporal and spatial effects [68].

The variance parameters for the random effects were assigned non-informative priors due to lack of prior corresponding data to inform the choice of such specification and allow the data to drive the model results [69]. The hyper prior distributions followed inverse gamma distributions with parameter values of 0.5 and 0.0005 [68]. The inference was made via Markov Chain Monte Carlo and posterior distributions of parameters summarised by the mean and the 95% credible intervals (CI).

#### Counterfactual analysis

The estimated regression coefficients from the space–time model were used to compute annual counterfactual U5M for every county between 1993 and 2014; U5M predictions assuming intervention coverage and disease prevalence of each factor had stayed constant at its 1993 value over 22 years. The differences between the observed U5M and counterfactual U5M were multiplied by the annual number of under-fives to compute the annual number of U5-DA and/or U5-LL between 1993 and 2014 based on census data including the corresponding CI. In summary, the set-up allowed for the estimation of the annual number of child deaths averted associated with changes in disease prevalence and intervention coverage relative to 1993 values.

Model convergence was evaluated via trace plots and Gelman–Rubin statistic [70] while the model accuracy was assessed using the MC error, the standard deviation and their ratio [71]. Data preparation and pre-processing were done in StataCorp. 2014 [Stata Statistical Software: Release 14. College Station, TX: StataCorp LP]; model development was conducted in R statistical (V.3·4·1) while the final Bayesian ecological space–time mixed-effects regression was fitted in WinBUGS Package (version 1.4.3) [72]. All the cartographies were done in ArcMap 10.5 (ESRI Inc., Redlands, CA, USA).

## Results

### Model development

A set of ten parsimonious factors were retained from the modelling building process and included in the Bayesian ecological space–time mixed-effects regression; the details are presented in Additional file 2. In summary, 17 factors were excluded to reduce collinearity, circularity and confounding; four factors not statistically significant and nine factors explaining the least U5M variation from ENR were excluded. Consequently, the best model based on information criterion and out of sample predictive accuracy included health facility deliveries, mothers with high parity, fully immunised status, households with access to better sanitation, proportion of children seeking treatment after fever, HIV and malaria infection prevalence, infants’ breastfeeding within the first hour of birth, proportion of stunted children and maternal autonomy (proxied by the proportion of female-headed households).

### Adjusted regression model

Early breastfeeding, access to better sanitation, fever treatment-seeking, maternal autonomy, facility deliveries and high parity were associated with a decrease in U5M while HIV and malaria infection prevalence were associated with an increase in U5M (Table 2). Fully immunised status and prevalence of stunting were not statistically significant. The magnitude of the effect was uneven across the ten factors with high parity and maternal autonomy, and HIV and malaria infection prevalence strongly and significantly associated decreasing and increasing U5M respectively. Overall HIV infection prevalence (2.87 [95% CI 2.03–3.72]) and high parity (− 2.05 [− 2.33 to − 1.80]) were significantly associated with the largest changes in U5M. Spatial variation was more dominant compared to temporal heterogeneity (Table 2) over the 22 years.

**Table 2:** The mean regression coefficients, 2.5–97.5% quantiles effects from the ecological Bayesian spatio-temporal mixed-effect regression model. The intercept represents the overall baseline. Sigma.w and sigma.t are the variances for spatial and temporal random effects, respectively; *Sigma.nu[1]* and *[2]* are space–time interaction effects for the stable (structured) and unstable (unstructured) risk patterns. The SD, MC error and their ratio are shown

Determinant	Mean [2.5–97.5%]	SD	MC error	Ratio
Breastfed within 1 h	− 0.39450 [− 0.52450 to − 0.26490]	0.06628	0.00085	1.3%
Better sanitation	− 0.61430 [− 0.73030 to − 0.49770]	0.05935	0.00112	1.9%
Female-headed household	− 1.05600 [− 1.24800 to − 0.86360]	0.09821	0.00124	1.3%
Fever treatment-seeking	− 0.56290 [− 0.78010 to − 0.34570]	0.11080	0.00127	1.2%
HIV risk	2.87300 [2.02500–3.72300]	0.43160	0.00894	2.1%
Health facility delivery	− 0.19650 [− 0.35470 to − 0.03879]	0.08057	0.00138	1.7%
High parity	− 2.05400 [− 2.31500 to − 1.79200]	0.13310	0.00186	1.4%
Malaria risk	0.12810 [0.05127–0.20550]	0.03936	0.00039	1.0%
Stunted	0.22580 [− 0.11780–0.57120]	0.17660	0.00279	1.6%
Fully immunised	− 0.11800 [− 0.25840–0.02259]	0.07179	0.00087	1.2%
Intercept	− 0.83470 [− 1.10600 to − 0.57020]	0.13650	0.00234	1.7%
Sigma.nu[1]	0.00544 [0.00027–0.01604]	0.00434	0.00015	3.4%
Sigma.nu[2]	0.10210 [0.07864–0.12870]	0.01267	0.00025	2.0%
Sigma.t	0.05245 [0.03791–0.07311]	0.00903	0.00004	0.4%
Sigma.w	0.57350 [0.46660–0.71040]	0.06225	0.00028	0.5%

### National counterfactual U5M

**Fig. 1:**
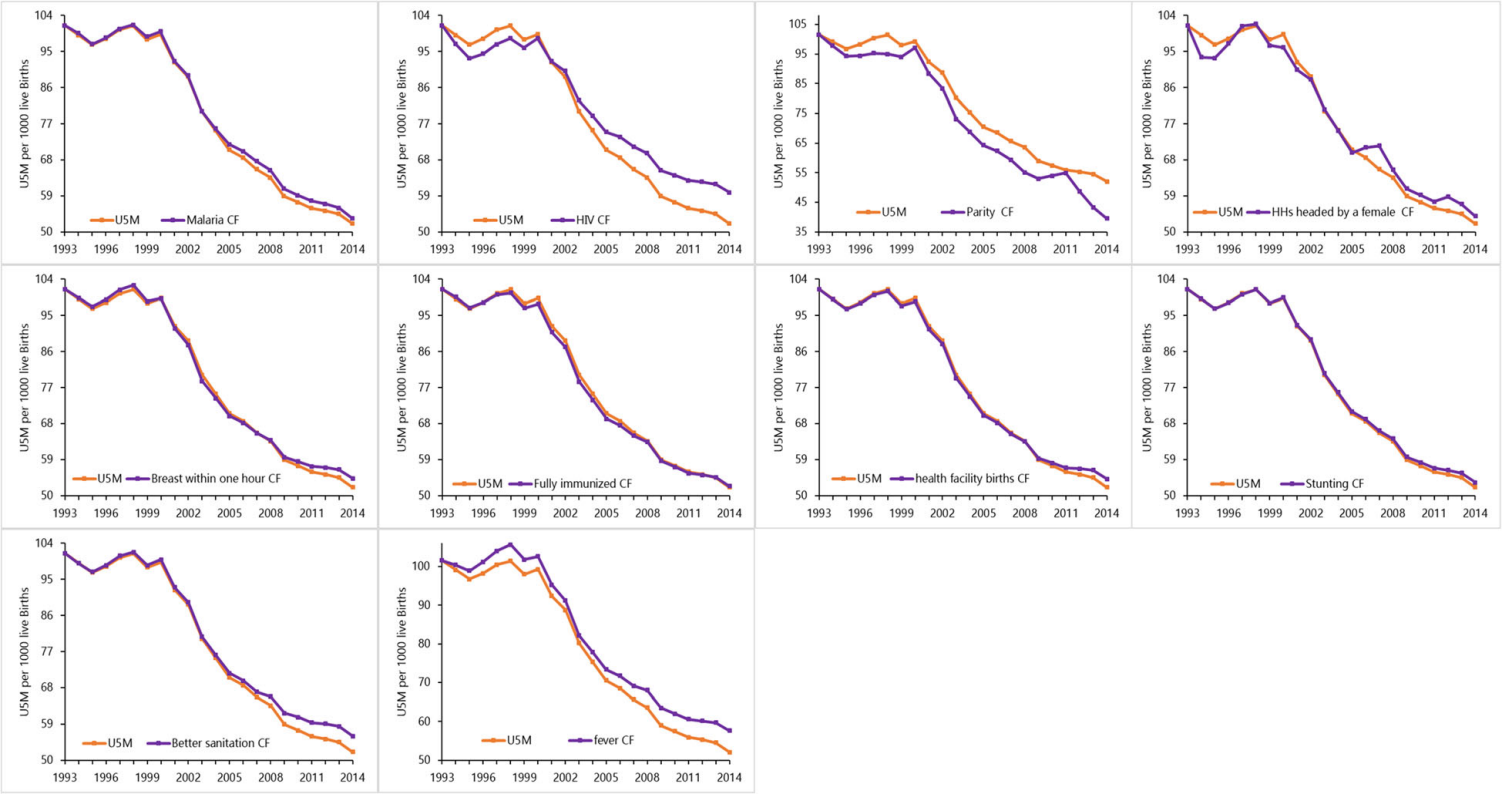
The national counterfactual and observed U5M for ten factors significantly associated with child survival in Kenya between 1993 and 2014. The observed national U5M is shown in orange [1] while the counterfactual U5M (U5M if each factor had remained at its 1993 level) is shown in purple. Source: author

Nationally, the roles of the ten key factors were heterogeneous over the 22-year period. Declining high parity and reduction in the proportion of fully immunised children were associated with U5-LL, while the increase in the coverage of early breastfeeding, access to better sanitation, seeking treatment after fever, maternal autonomy, facility deliveries and reduction in HIV and malaria infection prevalence were associated with U5-DA (Table 3, Fig. 1). The prevalence of malaria and HIV, high parity, better sanitation and fever treatment-seeking had much larger counterfactual impact compared to other factors. Across the 22 years, there were two important epochs: 1993–2000 when U5M either stagnated or increased with a high number of U5-LL witnessed and 2006–2014 epoch when U5M declined with a high number of U5-DA.

**Table 3:**
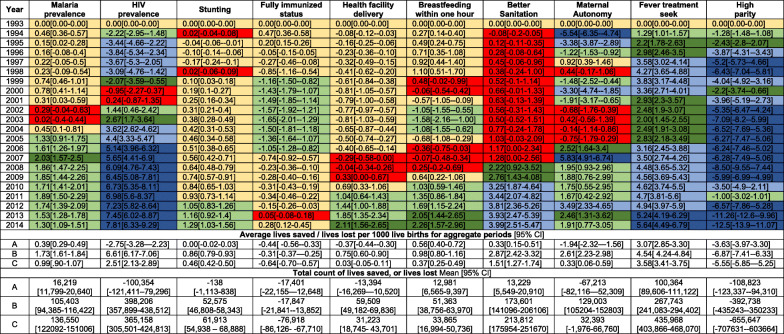
Annual under-five deaths averted [+ positive sign] or lives lost [− negative sign] per 1000 live births at the national level if the coverage/prevalence of each factor had remained unchanged between 1993 to 2014. They have been classified into < 1.0 (yellow), 1–2 (light green), 2–3 (dark green), 3–5 (light blue) and > 5 (dark blue). Red shows non-significant changes. Total counts and the average lives saved or lives lost for 1993–2000 [A], 2006–2014 [B] and 1993–2014 [C] are also presented

The period of rising U5M was largely associated with increasing HIV infection prevalence, reduction in maternal autonomy and decreasing high parity (Table 3, Fig. 1). Just over 100,000 (100,354, 95% CI 121,411–79,296) under-five lives would have been saved if HIV prevalence had remained the same as the level in 1993 through to 2000. Similarly, the decline in maternal autonomy was associated with 67,213 (52,309–82,116) U5-LL while a decline in high parity was associated with over 108,823 (94,310–123,337) U5-LL. The increase in the number of stunted children and the decline in both the number of children who were fully immunised children and the number of health facility deliveries were also associated with the stagnation; however, these seemed to have less of a counterfactual impact on U5-LL relative to HIV and maternal autonomy. However, during the stagnation epoch (1993–2000), the increase in fever treatment-seeking, early breastfeeding and declining malaria prevalence were associated with 100,364 (89,606–111,122), 12,981 (6565–9397) and 16,219 (11,799–20,640) deaths averted, respectively (Table 3).

The period of U5M decline (2006–2014) was characterised with substantial increases in the number of child deaths that were averted. The high number of U5-DA was associated with declining HIV infection prevalence 398,206 (357,899–438,512), reduction in malaria infection prevalence 105,403 (94,385–116,422) and a decline in the number of stunted children 52,575 (46,808–58,343). Increasing fever treatment-seeking rates 267,743 (241,083–294,402), access to better sanitation 173,601 (141,096–206,106), increasing maternal autonomy 129,003 [105,204–152,803], improvements in the coverage of early breastfeeding 51,363 [38,756–63,970] and institutional deliveries 59,509 (49,182–69,836) further contributed to deaths averted during 2006–2014 (Table 3, Fig. 1). However, reductions in the proportion of fully immunised children and high parity women were associated with 17,847 (3852–21,841) and 392,738 (350,233–435,243) U5-LL in the same timeline.

### County-level counterfactual U5M

**Fig. 2:**
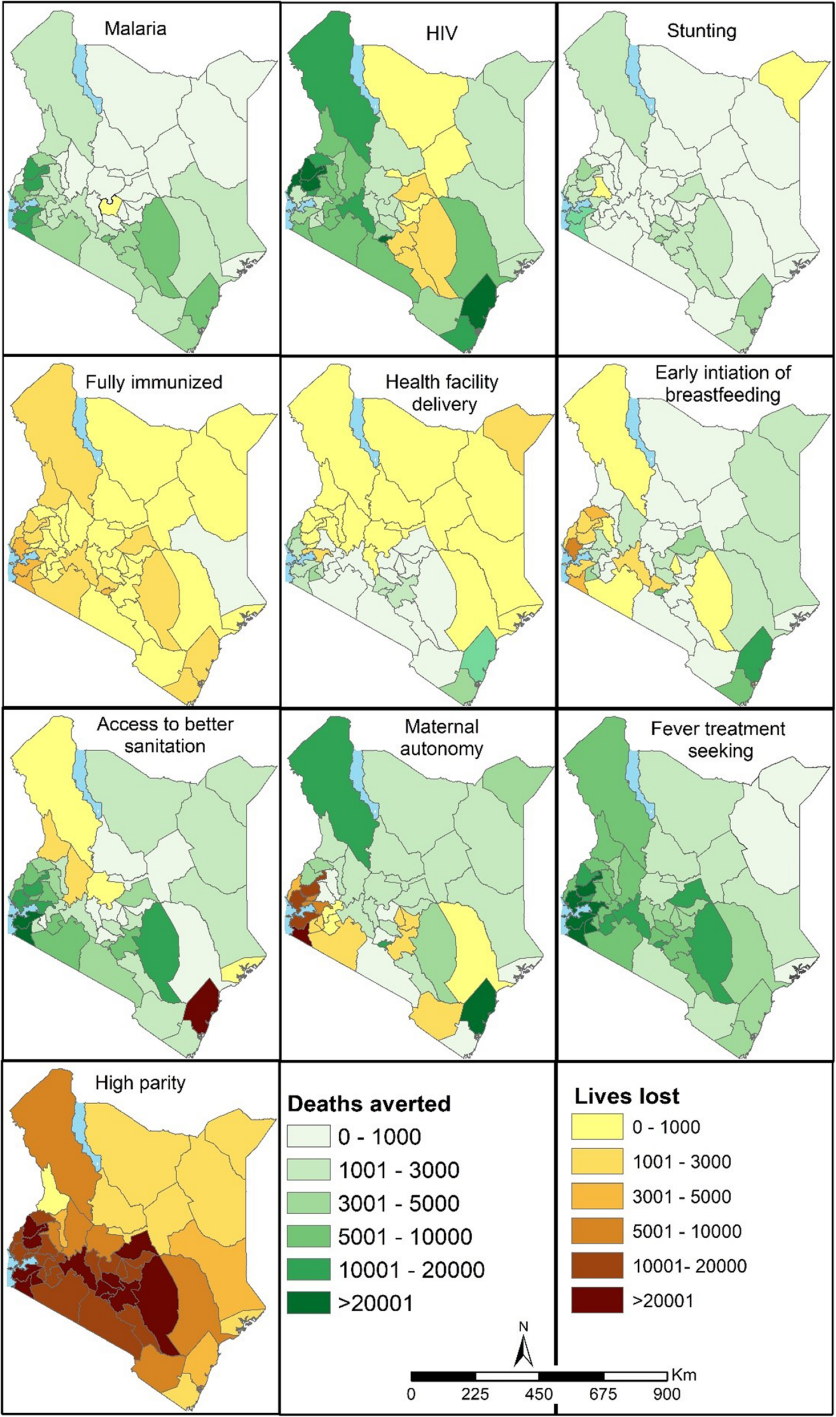
Total number of under-five deaths averted (green shades) and lives lost (yellow to brown) aggregated over 22 years [1993–2014] per county if the coverage/prevalence of factors relative to 1993 values had remained unchanged. Source: author

National-level presentation of U5-DA masks county-level variation. Figure 2 shows the number of U5-DA and U5-LL aggregated over the 22 years for the ten factors at the county level. Across the 22 years, the majority of the factors (increasing coverage and reduction in disease prevalence) across most of the counties were associated with U5-DA except for a decline in high parity and proportion of fully immunised children which were linked to U5-LL. Further, in the 22-year period, some regions recorded U5-LL: counties in northern (decline in health facility deliveries and better sanitation), eastern (slow reduction in HIV prevalence), and western (declining maternal autonomy and early breastfeeding) Kenya (Fig. 2).

There was high spatial heterogeneity in both the aggregated U5-DA and U5-LL across the counties. Counties in northern Kenya had a lower number of U5-DA associated with the declining prevalence of malaria and HIV, reduction in stunting, increase in access to better sanitation and fever treatment-seeking rates relative to other parts of Kenya which had higher rates of intervention coverage. In this region, U5-LL were associated with a decline in health facility deliveries and access to better sanitation; however, the region had a higher number of U5-DA relative to other parts of Kenya associated with an increase in maternal autonomy and proportion of infants whom breastfeeding was initiated early (Fig. 2).

Western Kenya had a larger number of U5-DA associated with increasing fever treatment-seeking rates, health facility deliveries, access to better sanitation and reduction of malaria and HIV in infection prevalence. The U5-LL in this region was associated with a decline in maternal autonomy and early breastfeeding (Fig. 2). Relative to western Kenya, the south-east region had almost a similar number of U5-DA associated with the same factors; however, some counties alternated between U5-DA and U5-LL associated with fluctuating changes in maternal autonomy and early breastfeeding (Fig. 2). Central Kenya had a moderate number of U5-DA when compared to other parts of the country. Declining high parity and fully immunised children were associated with U5-LL in all counties with considerable spatio-temporal variation (Fig. 2).

**Fig. 3:**
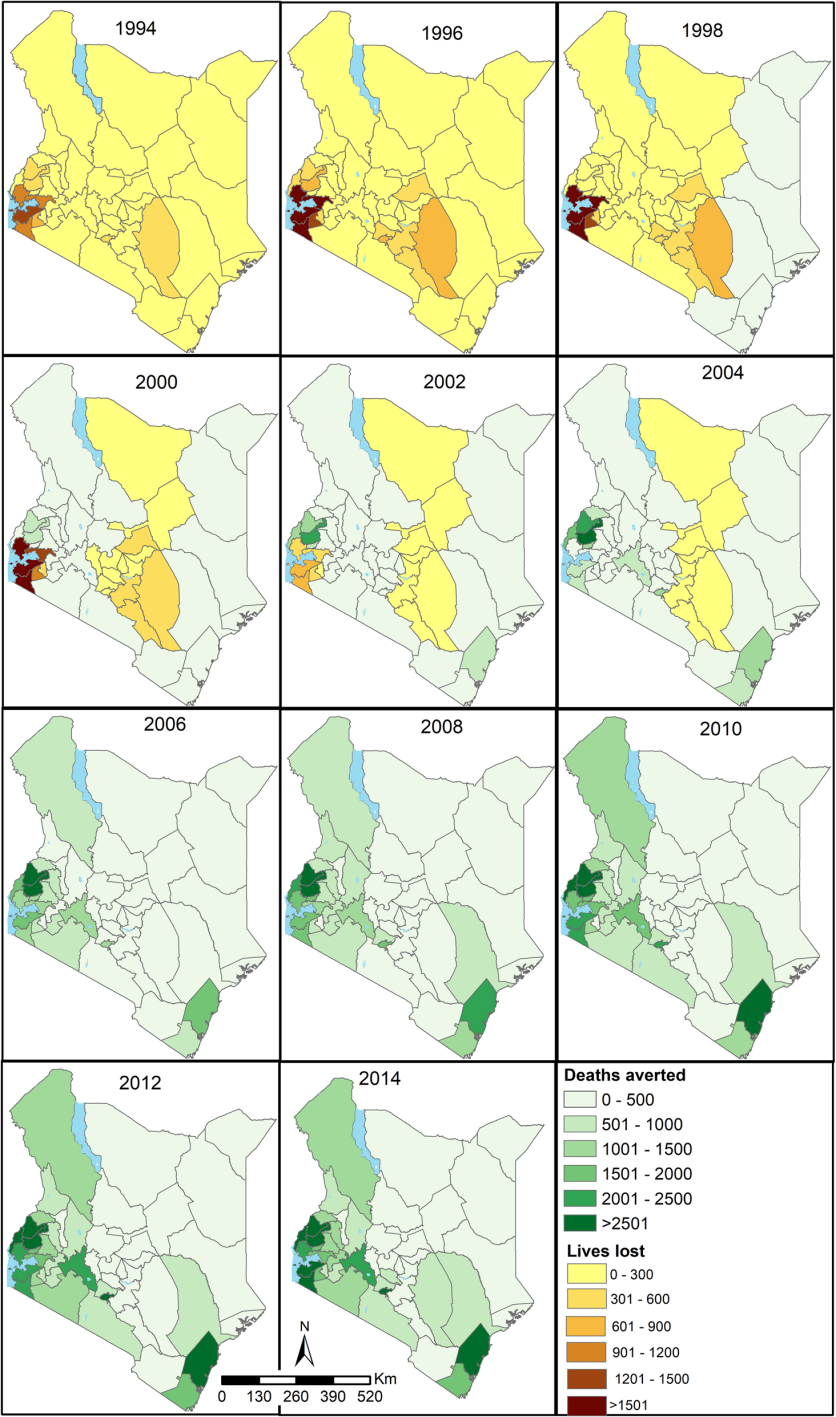
Number of deaths averted (green shades) and lives lost (yellow to brown) per year [1994–2014] per county if HIV infection prevalence relative to 1993 had remained unchanged. The maps for the other nine factors are presented in Additional file 3. Source: author

The aggregated values presented in Fig. 2 mask important spatial variations over time across individual factors. To highlight the variability, the counterfactual U5M for HIV infection prevalence is presented in Fig. 3 while the rest of the factors are presented in Additional file 3. The period between 1993 and 1997 was associated with increases in HIV infection prevalence (hence U5-LL) across all counties (Fig. 3) with a higher burden in western and parts of central Kenya. A transition phase followed from 1998 to 2005. After 2006, declining HIV infection prevalence was associated with U5-DA with the largest reductions in western and south-east Kenya. Comparable patterns of variability were observed for other factors (Additional file 3).

**Fig. 4:**
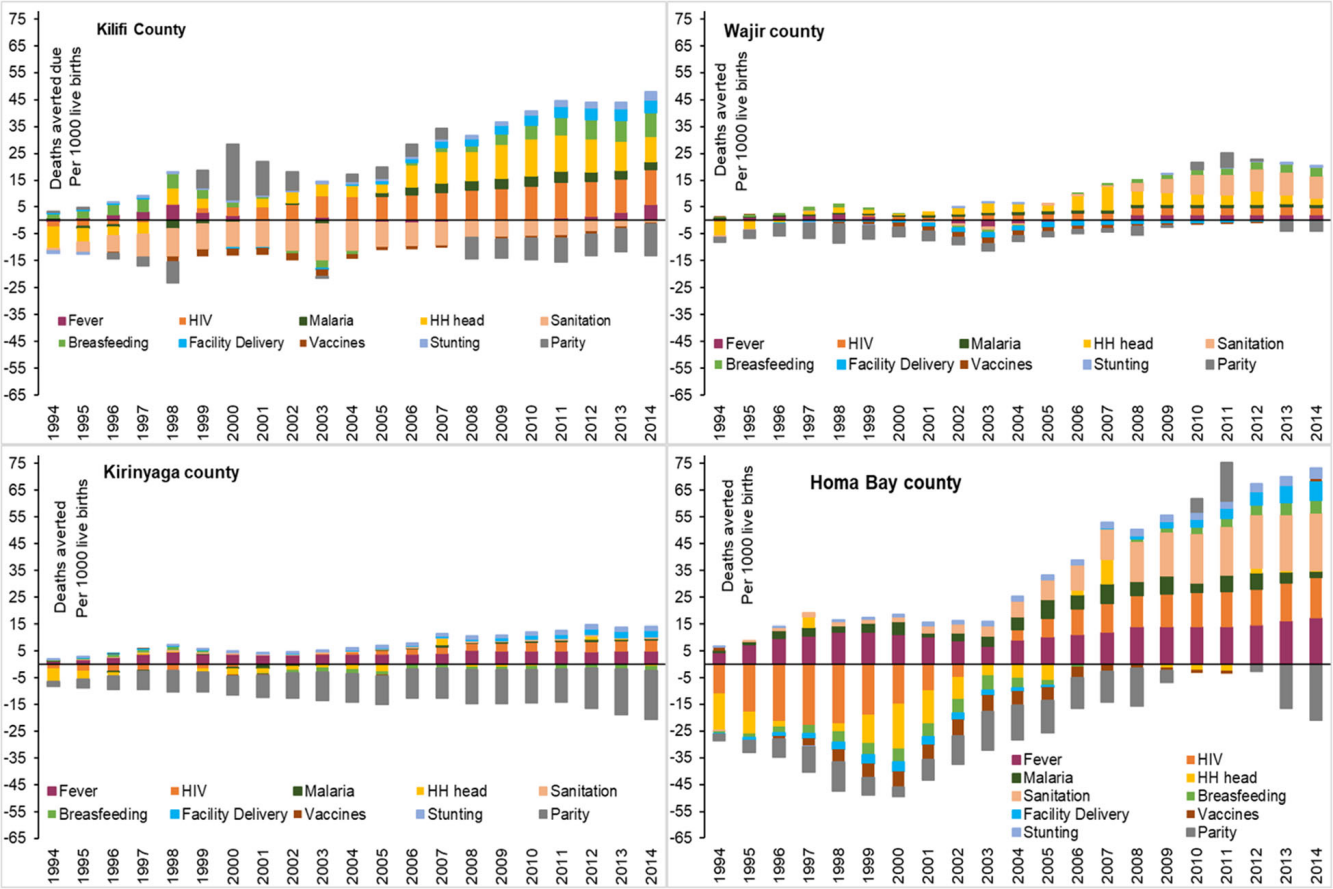
Comparison of deaths averted and live lost associated with changes in the coverage and/or prevalence of key factors relative to 1993 in Kilifi, Wajir, Homa Bay and Kirinyaga counties. All the counties are presented in Additional file 3. The magnitude of each colour represents the U5-DA (above zero-line) or U5-LL (below zero-line) per 1000 live births if the coverage/prevalence of the factor relative to 1993 had remained unchanged. Source: author

When compared simultaneously across all the factors and years in epidemiologically diverse counties (Kilifi, Homa Bay, Wajir and Kirinyaga) [1], the contribution of each factor in each region was extremely variable (Fig. 4). Temporal heterogeneity was evident, where a factor contributed to lives lost and at a different year it was associated with deaths averted relative to the baseline. Plots for all the 47 counties are presented in Additional file 3. Finally, the convergence and stabilisation of the Bayesian ecological space–time mixed-effects regression model were achieved and the accuracy was within the recommended rule of thumb [70, 71] (Additional file 2).

## Discussion

The analysis extends previous efforts to understand the impact of factors associated with child survival in Kenya [4–6, 19–24], incorporating more data, the bulk of factors in the continuum of child survival [33–36] over two decades anchored at sub-national counties used for decentralised health planning. Overall, stagnation and increase in U5M witnessed in the 1990s were likely due to increasing HIV infection prevalence and reduced maternal autonomy while the reduction in U5M observed after 2006 was likely associated with the declining HIV and malaria infection prevalence and increase in access to better sanitation, treatment-seeking and maternal autonomy. The decline in the number of stunted children and increase in early breastfeeding and institutional deliveries were likely to have had much smaller contribution to declining U5M while declining parity and proportion of fully immunised children were associated with increased childhood mortality.

The significant role of HIV on U5M variation over time has previously been observed in Kenya [5, 6, 22]. Since 2000, there have been concerted efforts to reduce and prevent HIV infection given impetus by the formation of Kenya’s National AIDS Control Council [4, 20]. Prevention of mother-to-child transmission, increased paediatric HIV programmes, antiretroviral drug uptake, testing and behavioural change campaigns increased steadily from early 2000 [73–77] contributing to improvements in child survival. The decline in malaria conspicuous after 2003 has been linked to the increased coverage of ITNs and changing antimalarial drug policies and contributed to declines observed in U5M during this period [4, 7, 78, 79].

Declines in stunting and increase in early breasting appear to have contributed moderately to the observed reduction in U5M. Increased breastfeeding can be linked to the promotion of breastfeeding in maternity wards and at the community level while complementary feeding, maternal nutrition, food fortification, micronutrient supplements, nutritional campaigns and school feeding programmes [80, 81] may have contributed to the reduction in the number of stunted children. However, the rate of decline for stunting was much smaller and slower as has previously documented [82, 83] likely due to poverty, low education attainment and lack of basic preventive health care in some regions [84]. Since 2003, the number of children receiving the minimum acceptable diet has also been low and declined over time [8].

Febrile illness is associated with a broad range of childhood illnesses; hence, fever treatment-seeking patterns provide insights on how the community seeks paediatric care [85–87]. The increasing number of deaths averted likely due to increasing treatment-seeking rates coincided with improved healthcare utilisation linked to partial abolishment of user fees (2004), direct health facility financing (2010) and free services at government outpatient facilities (2013) allowing better access to treatment [88–93]. The health voucher programme for maternity services (2006–2016), the abolishment of delivery fees (2007) and free maternity services (2013) may have led to increased facility delivery and likely contributed to improvement in child survival after 2008 [88–93]. The stagnation and small drop of children who were fully immunised could be due to non-timely immunisation, drop-out, demand and supply challenges related to physical access, health workforce, stockouts and transportation costs [94–97].

Maternal autonomy is context-specific due to differences in culture and community norms and takes various constructs [98]. In this work, maternal autonomy was defined by *households headed by a female* due to its ubiquitous availability across the surveys and a harmonised data collection approach over the years. It was associated with improved child survival (U5-DA) given mothers prioritise expenditure on basic food and health care above other needs compared to the fathers [98, 99]. High parity is routinely associated with high U5M (Additional file 1), and in this analysis, it was associated with an improvement in child survival; however, because high parity declined over time, under-five lives were lost. Such findings have been observed in different settings and have been linked to learning effect, reverse causality, sibling effect, hygiene hypothesis and residual confounding [100–104]. Nevertheless, we are unsure of the pathways through which high parity acted in the Kenyan context.

Sub-national heterogeneity observed in the role of the ten factors on U5M variation over the two decades has important consequences for the planning and prioritisation of health resources in Kenya. The factors associated with national improvements in child survival differed between counties. To reduce inequities and increase the likelihood of achieving health-related SDGs, there is a need to focus on the factors with the largest influence, most appropriate for individual counties. To make sure no one is left behind and those farthest behind are reached first, the national government could proportionally allocate funds based on U5M trends, intervention coverage and relative impact in averting child deaths. The counties can localise and tailor the resource to suit their context to achieve maximum gains.

In western Kenya, to sustain the gains and achieve further reductions in U5M, treatment-seeking rates and coverage of institutional deliveries and access to better sanitation should be improved and infection prevalence of HIV and malaria reduced further. Notably, counties located in western Kenya experience the highest levels of HIV and malaria risk and these counties already benefit from targeted HIV and malaria control viz. a viz. other counties [105]. In north-eastern Kenya, addressing access-related issues, nutritional programmes and access to improved sanitation will accelerate reductions in U5M while malaria in these semi-arid areas is not an important driver of U5M.

It should be noted that, despite statistical models showing a discrete number of major factors that explained most of the U5M variation, this does not imply that the excluded factors were not important in improving child survival. Some factors may have reached an early point of universal coverage and can no longer be used to explain contemporary variations in U5M or the counterfactual. Failure to recognise the continued importance of these factors, for example, continued breasting, at least one antenatal care visit, and BCG vaccination, could risk gains made pre-1990s. Different local settings within country present unique ways on how factors interact and co-exist within an ecosystem and their relationship with child survival. The factors whose association was not statistically significant or had smaller contributions could be due to several limitations.

### Limitations

Birth histories used to compute U5M are prone to misreporting of dates, maternal age and omissions of the dead children. Human resources for health, civil unrest and other macro-level factors were not included due to lack of data while recall biases associated with some factors were minimised by limiting the recall period to 3 years [106]. Further, the coverage estimates neither reflect the quality of interventions received nor do they measure effective coverage. The statistical inferences at the county level are prone to ecological fallacy [107] and the modifiable areal unit problem [108]. Some heterogeneities were masked especially in geographically larger counties of northern Kenya; disaggregation to lower administrative units would lower the precision. The counterfactual distribution cannot be observed in reality when using observation data; hence, changes that might have been triggered in the causal web remain unknown and part of the observed association might be due to confounding; therefore, cause and effect cannot be conclusively inferred [28, 32, 109]. Our analyses included data up to 2014 when the last household sample survey was conducted. The increased availability (quantity and quality) of data from Kenya’s routine health information system since 2011, the recently concluded housing and population census (2019) and ongoing household sample surveys (Malaria Indicator Survey) provides opportunities for updating both U5M and factors associated with child survival [110–112].

## Conclusions

During the MDG period, U5M reduction, intervention coverage increases and reduction on disease prevalence were characterised by sub-national disparities and inequities across Kenya. Ten factors were significantly associated with the majority of deaths averted or lives lost. The deaths averted were uneven in time between counties and are likely due to a decrease in the infection prevalence of HIV and malaria and an increase in access to better sanitation, treatment-seeking and maternal autonomy. A decline in the prevalence of stunting, progress in the initiation of early breastfeeding and an increase in institutional deliveries were associated with a moderate number of under-five deaths averted. Lives lost were associated with declining high parity and proportion of children who were fully immunised. The findings have improved our understanding of what factors were associated with variation in U5M in different counties over time to inform targeting and planning by decision-makers so that the gains made can be sustained and further accelerated. The results can be used to shape programmatic planning in the devolved governance structure in Kenya during the SDG era and Vision 2030, Kenya’s blueprint to providing a high quality of life to all its citizens by 2030 in a clean and secure environment.

## Supplementary Information

**Additional file 1.** Map of Kenya showing counties (Section 1), the conceptual framework on factors associated with child survival based on literature (Section 2), definitions of factors considered and the their data sources (Section 3).

**Additional file 2.** Model development framework to select a set of factors significantly associated with child survival in Kenya between 1993 and 2014 (Section 1) and model diagnostics (Section 2).

**Additional file 3.** Annual number of deaths averted, and lives lost (1994–2014) per county if coverage/ prevalence relative to 1993 had remained unchanged for factors associated with child survival (Section 1) and their comparison via an overlay of all ten factors per county (Section 2).

## Data Availability

The datasets generated and/or analysed during the current study either are available in the manuscript, based on previously published work, Macharia et al. [1], or are available open access from online data repositories for registered users in the following data portals: Integrated Public Use Microdata Series (IPUMS)—https://international.ipums.org/international/index.shtml Multiple Indicator Cluster Surveys (MICS)—http://mics.unicef.org/ Demographic and Health Surveys (DHS)—https://dhsprogram.com/ Kenya National Bureau of Statistics (KNBS)—http://statistics.knbs.or.ke/nada/index.php/home Population health Harvard Dataverse—https://dataverse.harvard.edu/dataverse/population-health

## Abbreviations

AIC: Akaike Information Criterion
ANC1: At least one antenatal care visit
ANC4: At least four antenatal care visits
BCG: Bacille Calmette–Guérin
CAR: Conditional autoregressive
CI: Credible interval
DIC: Deviance information criterion
DPT3: Three diphtheria–tetanus–pertussis vaccinations
ENR: Elastic net regression
EVI: Enhanced vegetation index
HFD: Health facility deliveries
HIV: Human immunodeficiency virus
IPTp: Intermittent preventive treatment in pregnancy
ITN: Insecticide-treated bed net
LBW: Low birth weight
MDGs: Millennium development goals
ORS: Oral rehydration salts
polio3: Three doses of polio
SBA: Skilled birth attendance
SDGs: Sustainable development goals
U5-DA: Under-five deaths averted
U5-LL: Under-five lives lost
U5M: Under-five mortality
VIF: Variance inflation factor

## References

[1] Macharia PM, Giorgi E, Thuranira PN, Joseph NK, Sartorius B, Snow RW, Okiro EA (2019). Sub national variation and inequalities in under-five mortality in Kenya since 1965. BMC Public Health.

[2] UNDESA (2015). The 17 sustainable development goals.

[3] Okiro EA (2019). Estimates of subnational health trends in Kenya. Lancet Glob Heal.

[4] Keats EC, Ngugi A, Macharia W, Akseer N, Khaemba EN, Bhatti Z, Rizvi A, Tole J, Bhutta ZA (2017). Progress and priorities for reproductive, maternal, newborn, and child health in Kenya: a countdown to 2015 country case study. Lancet Glob Heal.

[5] Opiyo CO, Sawhney M (2014). Determinants of the recent rise in childhood mortality in sub-Saharan Africa: evidence from Kenya demographic and health surveys, 1990-2003. Etude la Popul Africaine.

[6] Wafula SW, Ikamari LDE, K’Oyugi BO (2012). In search for an explanation to the upsurge in infant mortality in Kenya during the 1988-2003 period. BMC Public Health.

[7] Macharia PM, Giorgi E, Noor AM, Waqo E, Kiptui R, Okiro EA, Snow RW (2018). Spatio-temporal analysis of Plasmodium falciparum prevalence to understand the past and chart the future of malaria control in Kenya. Malar J.

[8] Matanda DJ, Urke HB, Mittelmark MB (2016). Changes in optimal childcare practices in Kenya: insights from the 2003, 2008-9 and 2014 demographic and health surveys. Plos One.

[9] Sartorius BKD, Sartorius K (2014). Global infant mortality trends and attributable determinants – an ecological study using data from 192 countries for the period 1990–2011. Popul Health Metrics.

[10] Sartorius BK, Kahn K, Vounatsou P (2010). Young and vulnerable: spatial-temporal trends and risk factors for infant mortality in rural South Africa (Agincourt), 1992-2007. BMC Public Health.

[11] Boco AG (2010). Individual and community-level effects on child mortality: an analysis of 28 demographic and health surveys in sub-Saharan Africa. DHS Work. Pap.

[12] Sartorius B, Veerman LJ, Manyema M, Chola L, Hofman K (2015). Determinants of obesity and associated population attributability, South Africa: empirical evidence from a national panel survey, 2008-2012. Plos One.

[13] Akachi Y, Steenland M, Fink G (2018). Associations between key intervention coverage and child mortality: an analysis of 241 sub-national regions of sub-Saharan Africa. Int J Epidemiol.

[14] Sartorius BKD, Sartorius K, Chirwa TF, Fonn S (2011). Infant mortality in South Africa-distribution, associations and policy implications, 2007: an ecological spatial analysis. Int J Health Geogr.

[15] Landier J, Rebaudet S, Piarroux R, Gaudart J (2018). Spatiotemporal analysis of malaria for new sustainable control strategies. BMC Med.

[16] Byberg S, Østergaard MD, Rodrigues A, Martins C, Benn CS, Aaby P, Fisker AB (2017). Analysis of risk factors for infant mortality in the 1992-3 and 2002-3 birth cohorts in rural Guinea-Bissau. PLoS One.

[17] Marmot M, Bell R (2017). The sustainable development goals and health equity. Epidemiology.

[18] Stuart E, Woodroffe J (2016). Leaving no-one behind: can the sustainable development goals succeed where the millennium development goals lacked?. Gend Dev.

[19] Achoki T, Miller-Petrie MK, Glenn SD, Kalra N, Lesego A, Gathecha GK, Alam U, Kiarie HW, Maina IW, Adetifa IMO, Barsosio HC, Degfie TT, Keiyoro PN, Kiirithio DN, Kinfu Y, Kinyoki DK, Kisia JM, Krish VS, Lagat AK, Mooney MD, Moturi WN, Newton CRJ, Ngunjiri JW, Nixon MR, Soti DO, van de Vijver S, Yonga G, Hay SI, Murray CJL, Naghavi M (2018). Health disparities across the counties of Kenya and implications for policy makers, 1990–2016: a systematic analysis for the Global Burden of Disease Study 2016. Lancet Glob Heal.

[20] Keats EC, Macharia W, Singh NS, Akseer N, Ravishankar N, Ngugi AK, Rizvi A, Khaemba EN, Tole J, Bhutta ZA (2018). Accelerating Kenya’s progress to 2030: understanding the determinants of under-five mortality from 1990 to 2015. BMJ Glob Heal.

[21] Hategeka C, Tuyisenge G, Bayingana C, Tuyisenge L (2019). Effects of scaling up various community-level interventions on child mortality in Burundi, Kenya, Rwanda, Uganda and Tanzania: a modeling study. Glob Heal Res Policy.

[22] Hill K, Bicego G, Mahy M (2001). Childhood mortality in Kenya: an examination of trends and determinants in the late 1980s to mid 1990s.

[23] Demombynes G, Trommlerová SK (2016). What has driven the decline of infant mortality in Kenya in the 2000s?. Econ Hum Biol.

[24] Frings M, Lakes T, Müller D, Khan MMH, Epprecht M, Kipruto S, Galea S, Gruebner O (2018). Modeling and mapping the burden of disease in Kenya. Sci Rep.

[25] Rogers P. Overview: strategies for causal attribution. Methodol. Briefs Impact Eval. 2014:16. http://devinfolive.info/impact_evaluation/ie/img/downloads/Overview_Strategies_for_Causal_Attribution_ENG.pdf. Accessed 3 Aug 2020.

[26] Stern E, Stame N, Mayne J (2012). Broadening the range of designs and benefits of trade methods for impact evaluations: report of a study commissioned by the Department for International Development.

[27] Maldonado G, Greenland S (2002). Estimating causal effects. Int J Epidemiol.

[28] Yé Y, Eisele TP, Eckert E, Korenromp E, Shah JA, Hershey CL, Ivanovich E, Newby H, Carvajal-Velez L, Lynch M, Komatsu R, Cibulskis RE, Moore Z, Bhattarai A (2017). Framework for evaluating the health impact of the scale-up of malaria control interventions on all-cause child mortality in sub-Saharan Africa. Am J Trop Med Hyg.

[29] Murray CJL, Lopez AD (1999). On the comparable quantification of health risks: lessons from the global burden of disease study. Epidemiology.

[30] WHO (2002). Summary measures of population health: concepts, ethics, measurements and applications.

[31] Ezzati M, Hoorn S, Vander LAD, et al. Chapter 4: comparative quantification of mortality and burden of disease attributable to selected risk factors. Glob Burd Dis Risk Factors. 2006:241–68. 10.5860/CHOICE.44-4479. 21250375

[32] Murray CJ, Ezzati M, Lopez AD (2003). Comparative quantification of health risks: conceptual framework and methodological issues. Popul Health Metrics.

[33] Gakidou E, Cowling K, Lozano R, Murray CJL (2010). Increased educational attainment and its effect on child mortality in 175 countries between 1970 and 2009: a systematic analysis. Lancet.

[34] Verguet S, Nandi A, Filippi V, Bundy DAP (2016). Maternal-related deaths and impoverishment among adolescent girls in India and Niger: findings from a modelling study. BMJ Open.

[35] Makela SM, Dandona R, Dilip TR, Dandona L (2013). Social sector expenditure and child mortality in India: a state-level analysis from 1997 to 2009. Plos One.

[36] Ng M, Colson KE, Fullman N (2017). Assessing the contribution of malaria vector control and other maternal and child health interventions in reducing all-cause under-five mortality in Zambia. Am J Trop Med Hyg.

[37] Murray CJ, Lopez AD. The Global Burden of Disease: a comprehensive assessment of mortality and disability from diseases, injuries, and risk factors in 1990 and projected to 2020. 1. Havard University Press 1996. doi:10.1088/1742-6596/707/1/012025

[38] Mokdad AH, Jaber S, Abdel Aziz MI (2014). The state of health in the Arab world, 1990-2010: an analysis of the burden of diseases, injuries, and risk factors. Lancet.

[39] Hill K (2003). Frameworks for studying the determinants of child survival. Bull World Health Organ.

[40] Corsi DJ, Subramanian SV (2014). Association between coverage of maternal and child health interventions, and under-5 mortality: a repeated cross-sectional analysis of 35 sub-Saharan African countries. Glob Health Action.

[41] Mosley WH, Chen CL (1984). An analytical framework for the study of child survival in developing countries. Popul Dev Rev.

[42] Schell CO, Reilly M, Rosling H, Peterson S, Mia Ekström A (2007). Socioeconomic determinants of infant mortality: a worldwide study of 152 low-, middle-, and high-income countries. Scand J Public Health.

[43] NACC (2020). Kenya HIV prevalence estimates.

[44] Funk C, Peterson P, Landsfeld M, Pedreros D, Verdin J, Shukla S, Husak G, Rowland J, Harrison L, Hoell A, Michaelsen J (2015). The climate hazards infrared precipitation with stations - a new environmental record for monitoring extremes. Sci Data.

[45] Matsushita B, Yang W, Chen J, Onda Y, Qiu G (2007). Sensitivity of the enhanced vegetation index (EVI) and normalized difference vegetation index (NDVI) to topographic effects. Sensors.

[46] Babyak MA (2004). What you see may not be what you get: a brief, nontechnical introduction to overfitting in regression-type models. Psychosom Med.

[47] Murtaugh PA (2009). Performance of several variable-selection methods applied to real ecological data. Ecol Lett.

[48] Moraga P, Cano J, Baggaley RF, Gyapong JO, Njenga SM, Nikolay B, Davies E, Rebollo MP, Pullan RL, Bockarie MJ, Hollingsworth TD, Gambhir M, Brooker SJ (2015). Modelling the distribution and transmission intensity of lymphatic filariasis in sub-Saharan Africa prior to scaling up interventions: integrated use of geostatistical and mathematical modelling. Parasites Vectors.

[49] Deribe K, Fronterre C, Dejene T, Biadgilign S, Deribew A, Abdullah M, Cano J (2020). Measuring the spatial heterogeneity on the reduction of vaginal fistula burden in Ethiopia between 2005 and 2016. Sci Rep.

[50] Noor AM, Kinyoki DK, Mundia CW, Kabaria CW, Mutua JW, Alegana VA, Fall IS, Snow RW (2014). The changing risk of Plasmodium falciparum malaria infection in Africa: 2000-10: a spatial and temporal analysis of transmission intensity. Lancet.

[51] Snow RW, Kibuchi E, Karuri SW, Sang G, Gitonga CW, Mwandawiro C, Bejon P, Noor AM (2015). Changing malaria prevalence on the Kenyan coast since 1974: climate, drugs and vector control. Plos One.

[52] Brabin BJ (1983). An analysis of malaria in pregnancy in Africa. Bull World Health Organ.

[53] Brabin BJ. The risks and severity of malaria in pregnant women 1991;1:1–33.http://apps.who.int/iris/bitstream/10665/61511/1/TDR_FIELDMAL_1.pdf. Accessed 18 Oct 2016

[54] Guyatt HL, Snow RW (2001). The epidemiology and burden of Plasmodium falciparum-related anemia among pregnant women in sub-Saharan Africa. Am J Trop Med Hyg.

[55] Guyatt HL, Snow RWR (2004). Impact of malaria during pregnancy on low birth weight in sub-Saharan Africa. Clin Microbiol Rev.

[56] GoK MoH (2010). Kenya national malaria policy.

[57] Zou H, Hastie T (2005). Regularization and variable selection via the elastic net. J R Stat Soc Ser B Stat Methodol.

[58] Tibshirani R (1996). Regression shrinkage and selection via the lasso. J R Stat Soc Ser B.

[59] Waldmann P, Mészáros G, Gredler B, Fuerst C, Sölkner J (2013). Evaluation of the lasso and the elastic net in genome-wide association studies. Front Genet.

[60] Friedman J, Hastie T, Tibshirani R (2010). Regularization paths for generalized linear models via coordinate descent. J Stat Softw.

[61] Dwomoh D, Amuasi S, Agyabeng K, Incoom G, Alhassan Y, Yawson AE (2019). Understanding the determinants of infant and under-five mortality rates: a multivariate decomposition analysis of Demographic and Health Surveys in Ghana, 2003, 2008 and 2014. BMJ Glob Heal.

[62] Tagoe ET, Agbadi P, Nakua EK, Duodu PA, Nutor JJ, Aheto JMK (2020). A predictive model and socioeconomic and demographic determinants of under-five mortality in Sierra Leone. Heliyon.

[63] Walther BA, Moore JL, Rahbek C (2005). The concepts a literature with of species richness the performance estimators, of estimator review performance precision. Ecography (Cop).

[64] Vyas S, Kumaranayake L (2006). Constructing socio-economic status indices: how to use principal components analysis. Health Policy Plan.

[65] O’Brien RM (2007). A caution regarding rules of thumb for variance inflation factors. Qual Quant.

[66] Pezzulo C, Bird T, Utazi EC, *et al.* Geospatial modeling of child mortality across 27 countries in sub-Saharan Africa. DHS Spat. Anal. Reports No. 13. 2016.http://dhsprogram.com/pubs/pdf/SAR13/SAR13.pdf. Accessed 1 Jan 2016

[67] Besag J, York J, Mollié A (1991). Bayesian image restoration, with 2 applications in spatial statistics. Ann Inst Stat Math.

[68] Abellan JJ, Richardson S, Best N (2008). Use of space time models to investigate the stability of patterns of disease. Environ Health Perspect.

[69] Ruktanonchai CW, Nilsen K, Alegana VA, Bosco C, Ayiko R, Seven Kajeguka AC, Matthews Z, Tatem AJ (2018). Temporal trends in spatial inequalities of maternal and newborn health services among four east African countries, 1999–2015. BMC Public Health.

[70] Gelman A, Rubin DB (1992). Inference from iterative simulation using multiple sequences. Stat Sci.

[71] Spiegelhalter D, Thomas A, Best N, *et al.* WinBUGS user manual version 1.4 MRC Biostatistics Unit. 2003.http://citeseerx.ist.psu.edu/viewdoc/download?doi=10.1.1.726.775&rep=rep1&type=pdf. Accessed 5 Jan 2019

[72] Lunn DJ, Thomas A, Best N, Spiegelhalter D (2000). WinBUGS – a Bayesian modelling framework: concepts, structure, and extensibility. Stat Comput.

[73] Lupia R, Chien SC (2012). HIV and AIDS epidemic in Kenya: an overview. J Exp Clin Med.

[74] Grabbe KL, Menzies N, Taegtmeyer M, Emukule G, Angala P, Mwega I, Musango G, Marum E (2010). Increasing access to HIV counseling and testing through mobile services in Kenya: strategies, utilization, and cost-effectiveness. J Acquir Immune Defic Syndr.

[75] Marum E, Taegtmeyer M, Chebet K (2006). Scale-up of voluntary HIV counseling and testing in Kenya. J Am Med Assoc.

[76] Marum E, Morgan G, Hightower A, Ngare C, Taegtmeyer M (2008). Using mass media campaigns to promote voluntary counseling and HIV-testing services in Kenya. Aids.

[77] Kohler PK, Okanda J, Kinuthia J, Mills LA, Olilo G, Odhiambo F, Laserson KF, Zierler B, Voss J, John-Stewart G (2014). Community-based evaluation of PMTCT uptake in Nyanza Province, Kenya. Plos One.

[78] Noor AM, Amin AA, Akhwale WS, Snow RW (2007). Increasing coverage and decreasing inequity in insecticide-treated bed net use among rural Kenyan children. Plos Med.

[79] Amin AA, Zurovac D, Kangwana BB, Greenfield J, Otieno DN, Akhwale WS, Snow RW (2007). The challenges of changing national malaria drug policy to artemisinin-based combinations in Kenya. Malar J.

[80] Kavle JA, Ahoya B, Kiige L, Mwando R, Olwenyi F, Straubinger S, Gathi CM (2019). Baby-Friendly Community Initiative—from national guidelines to implementation: a multisectoral platform for improving infant and young child feeding practices and integrated health services. Matern Child Nutr.

[81] Maingi M, Kimiywe J, Iron-Segev S (2018). Effectiveness of Baby Friendly Community Initiative (BFCI) on complementary feeding in Koibatek, Kenya: a randomized control study. BMC Public Health.

[82] Masibo PK, Makoka D (2012). Trends and determinants of undernutrition among young Kenyan children: Kenya Demographic and Health Survey; 1993, 1998, 2003 and 2008-2009. Public Health Nutr.

[83] Matanda DJ, Mittelmark MB, Kigaru DMD (2014). Child undernutrition in Kenya: trend analyses from 1993 to 2008-09. BMC Pediatr.

[84] Kabubo-Mariara J, Ndenge GK, Mwabu DK (2009). Determinants of children’s nutritional status in Kenya: evidence from demographic and health surveys. J Afr Econ.

[85] D’Acremont V, Kilowoko M, Kyungu E (2014). Beyond malaria - causes of fever in outpatient Tanzanian children. N Engl J Med.

[86] WHO (1997). IMCI: the integrated approach.

[87] Alegana VA, Maina J, Ouma PO, Macharia PM, Wright J, Atkinson PM, Okiro EA, Snow RW, Tatem AJ (2018). National and sub-national variation in patterns of febrile case management in sub-Saharan Africa. Nat Commun.

[88] Dennis ML, Abuya T, Campbell OMR, Benova L, Baschieri A, Quartagno M, Bellows B (2018). Evaluating the impact of a maternal health voucher programme on service use before and after the introduction of free maternity services in Kenya: a quasi-experimental study. BMJ Glob Heal.

[89] Dennis ML, Benova L, Abuya T, Quartagno M, Bellows B, Campbell OMR (2019). Initiation and continuity of maternal healthcare: examining the role of vouchers and user-fee removal on maternal health service use in Kenya. Health Policy Plan.

[90] Gitobu CM, Gichangi PB, Mwanda WO (2018). The effect of Kenya’s free maternal health care policy on the utilization of health facility delivery services and maternal and neonatal mortality in public health facilities. BMC Pregnancy Childbirth.

[91] Dennis ML, Benova L, Goodman C, *et al.* Examining user fee reductions in public primary healthcare facilities in Kenya, 1997–2012: effects on the use and content of antenatal care. International Journal for Equity in Health. 2020;8:1–13. 10.1186/s12939-020-1150-8. 10.1186/s12939-020-1150-8 PMC7073011 32171320

[92] Janisch CP, Albrecht M, Wolfschuetz A, Kundu F, Klein S (2010). Vouchers for health: a demand side output-based aid approach to reproductive health services in Kenya. Glob Public Health.

[93] Abuya T, Njuki R, Warren CE, Okal J, Obare F, Kanya L, Askew I, Bellows B (2012). A policy analysis of the implementation of a reproductive health vouchers program in Kenya. BMC Public Health.

[94] Haji A, Lowther S, Ngan Z, *et al.* Reducing routine vaccination dropout rates: evaluating two interventions in three Kenyan districts, 2014. BMC Public Health 2016;16:1–8. doi:10.1186/s12889-016-2823-5, 1. 10.1186/s12889-016-2823-5 PMC4754928 26880141

[95] Gibson DG, Ochieng B, Kagucia EW, Obor D, Odhiambo F, O’Brien KL, Feikin DR (2015). Individual level determinants for not receiving immunization, receiving immunization with delay, and being severely underimmunized among rural western Kenyan children. Vaccine.

[96] Calhoun LM, Van Eijk AM, Lindblade KA (2014). Determinants and coverage of vaccination in children in Western Kenya from a 2003 cross-sectional survey. Am J Trop Med Hyg.

[97] GoK MoH (2010). Kenya DVI comprehensive multi-year plan 2006-2010.

[98] Carlson GJ, Kordas K, Murray-Kolb LE (2015). Associations between women’s autonomy and child nutritional status: a review of the literature. Matern Child Nutr.

[99] Mahapatro SR (2012). Utilization of maternal and child health care services in India: does women’s autonomy matter?. J Fam Welf.

[100] Jensen ER, Ahlburg DA (2002). Family size, unwantedness, and child health and health care utilisation in Indonesia. Bull Indones Econ Stud.

[101] Peters C, Rees DI, Hernández-Julián R (2014). The trade-off between family size and child health in rural Bangladesh. East Econ J.

[102] Kravdal Ø, Kodzi I (2011). Children’s stunting in sub-Saharan Africa: is there an externality effect of high fertility?. Demogr Res.

[103] Lundborg P, Ralsmark H, Rooth D (2013). The more the healthier? Health and family size.

[104] Kozuki N, Lee AC, Silveira MF (2013). The associations of parity and maternal age with small-for-gestational-age, preterm, and neonatal and infant mortality: a meta-analysis. BMC Public Health.

[105] MoH/GoK (2019). Kenya malaria strategy 2019–2023.

[106] Ngandu NK, Manda S, Besada D, Rohde S, Oliphant NP, Doherty T (2016). Does adjusting for recall in trend analysis affect coverage estimates for maternal and child health indicators? An analysis of DHS and MICS survey data. Glob Health Action.

[107] Wakefield J, Shaddick G (2006). Health-exposure modeling and the ecological fallacy. Biostatistics.

[108] Fotheringham AS, Wong DWS (1991). The modifiable areal unit problem in multivariate statistical analysis. Environ Plan A.

[109] Kulhánová I, Hoffmann R, Judge K, Looman CW, Eikemo TA, Bopp M, Deboosere P, Leinsalu M, Martikainen P, Rychtaříková J, Wojtyniak B, Menvielle G, Mackenbach JP, EURO-GBD-SE Consortium (2014). Assessing the potential impact of increased participation in higher education on mortality: evidence from 21 European populations. Soc Sci Med.

[110] KNBS. 2019 Kenya Population and Housing Census Reports 2019.https://www.knbs.or.ke/?p=5732. Accessed 22 Apr 2020

[111] Minnesota Population Center (2016). Integrated public use microdata series, international: version 6.5 [Kenya Census data].

[112] Alegana VA, Okiro EA, Snow RW (2020). Routine data for malaria morbidity estimation in Africa: challenges and prospects. BMC Med.

