# Supplementary material for: The impact of child health interventions and risk factors on child survival in Kenya, 1993–2014: a Bayesian spatio-temporal analysis with counterfactual scenarios
Source: BMC Med. 2021 May 4;19:102. doi: 10.1186/s12916-021-01974-x (PMC8094495; doi:10.1186/s12916-021-01974-x)
Supplement: Supplementary file 1 — Additional file 1. Map of Kenya showing counties (Section 1), the conceptual framework on factors associated with child survival based on literature (Section 2), definitions of factors considered and the their data sources (Section 3). [file 12916_2021_1974_MOESM1_ESM.docx]

**Additional File 1**

**Section 1**

Figure 1: The map of Kenya showing 8 provinces (coloured) and the 47 sub-national units (counties) as dark lines (see footnote), water bodies and major rivers are shown in blue. Source- Author


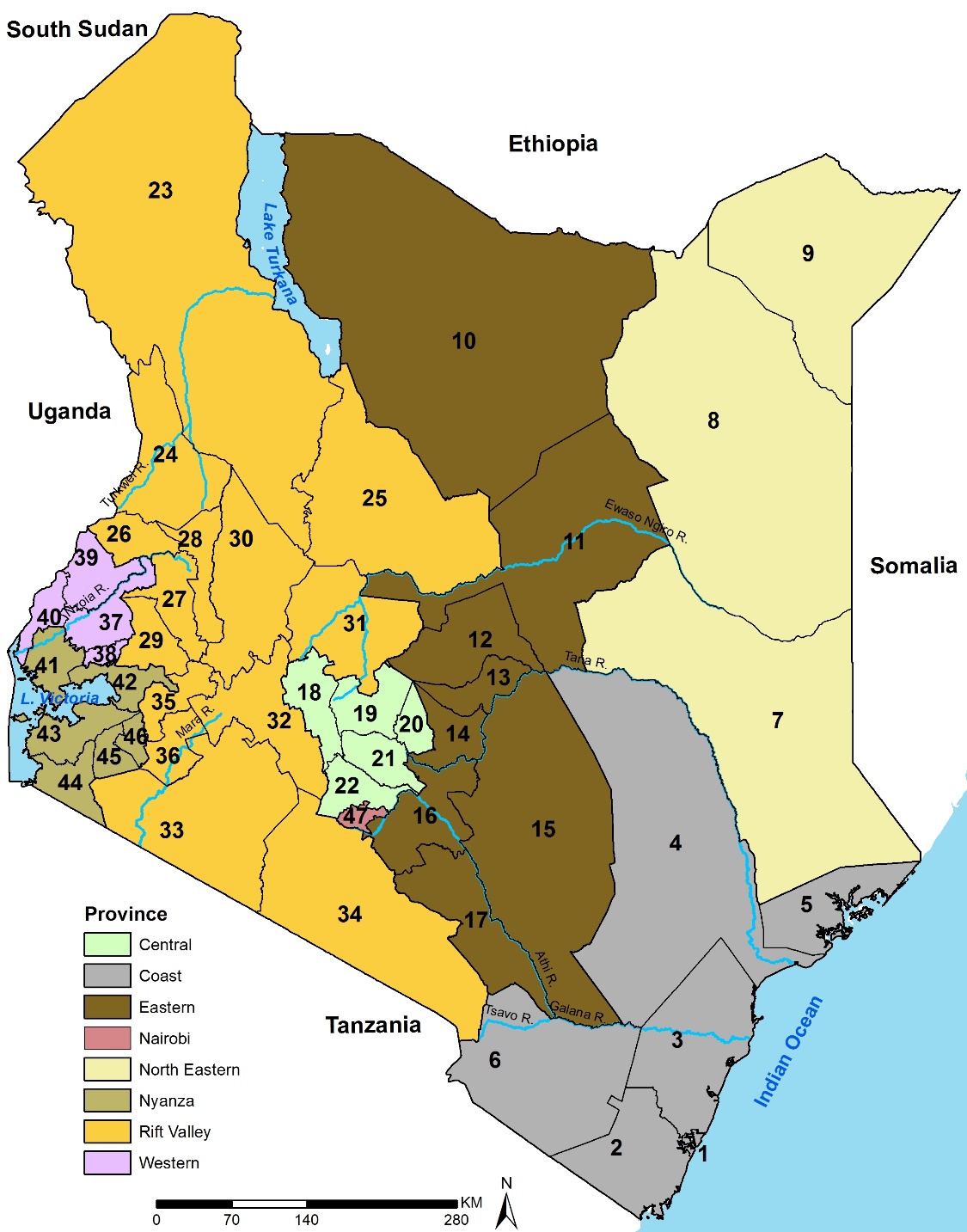


**Footnote Figure 1**: Coast province: Mombasa [1], Kwale [2], Kilifi [3], Tana River [4], Lamu [5], Taita Taveta [6]; North Eastern province: Garissa [7], Wajir [8], Mandera [9]; Eastern province: Marsabit [10], Isiolo [11], Meru [12], Tharaka Nithi [13], Embu [14], Kitui [15], Machakos [16], Makueni [17]; Central province: Nyandarua [18], Nyeri [19], Kirinyaga [20], Murang'a [21], Kiambu [22]; Rift Valley province: Turkana [23], West Pokot [24], Samburu [25], Trans Nzoia [26], Uasin Gishu [27], Elgeyo Marakwet [28], Nandi [29], Baringo [30], Laikipia [31], Nakuru [32], Narok [33], Kajiado [34], Kericho [35], Bomet [36]; Western province: Kakamega [37], Vihiga [38], Bungoma [39], Busia [40]; Nyanza province: Siaya [41], Kisumu [42], Homa Bay [43], Migori [44], Kisii [45], Nyamira [46]; Nairobi province: Nairobi [47].

**Section 2: Factors associated with under-five mortality (U5M)**

There are many conceptual frameworks of child survival [1–5]. The fundamental basis across the frameworks is that distal factors operate via intermediate then through proximate factors that directly influence the balance between health, morbidity and mortality. That is, child deaths are attributable to a wide range of factors related to each other hierarchically. Intermediate factors are usually at the interface of distal and proximal factors, however, some frameworks do not distinguish between proximal and intermediate factors [3].

The most widely used framework is the Mosely-Chen framework of *proximate determinants* [1,3,6,7]. Based on this framework, 97% of newborn are expected to survive through to age five in an ideal setting, and any reduction in child survival is due to the operation of social, economic, biological and environmental factors. The framework identifies proximate variables through which socio-economic factors act to influence morbidity and mortality [3,8]. The proximate determinants include maternal factors (age, parity, birth interval); environmental (water source), nutritional deficiency, injury (accidental, intentional) and personal illness control (such as medical treatment). These four affect the rate at which children move from healthy to sick, while personal illness control (prevention and treatment) affect this rate and that of recovery [1,3]. In the current analysis, the Mosely-Chen framework [3] formed the base framework which was adapted using other frameworks of child survival [2,4,5].

Further, the factors associated with U5M and how they affect child survival have been extensively reviewed in literature [9,10]. Roberts *et al* (2015), identified 20 maternal and child health (MCH) indicators in Uganda via in-country meetings with collaborators and relevant stakeholders [11]. Similar approaches were used to identify 20 MCH outcomes and interventions in Nigeria [12] and 17 MCH indicators in Zambia [13].

The process through which these factors operate to result in U5M is detailed in Figure 2 and expounded in Table 1. In the adapted framework (Figure 2), the factors associated with U5M have been divided broadly into two categories; distal and a combination of proximate and intermediate factors. The distal factors are classified into three broad categories which include maternal, societal, environmental and governmental. The distal variables act through a set of proximate/intermediate factors that include maternal, child and household factors that influence the balance between health and sickness and whether growth falters leading to death (Figure 2). The control of personal illness and the use of prevention measures (interventions) available at health facilities mediates between health, sickness and U5M. Access to intervention at the facilities is affected by access which has a bearing on utilization patterns (Figure 2).

Figure 2: Conceptual framework relating determinants of child survival and U5M based on literature [2–5]. Abbreviations are presented in Table 1


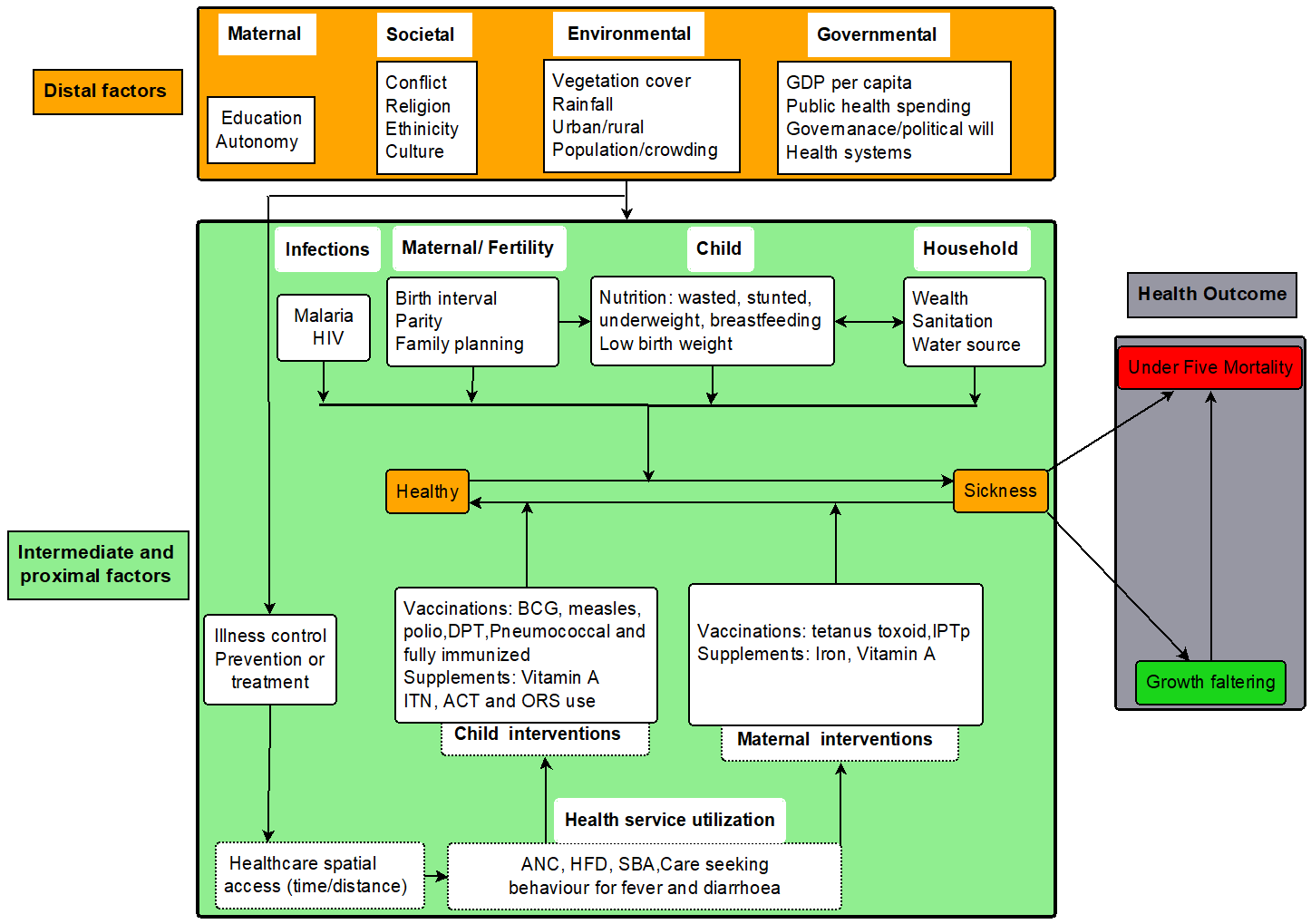


**Table 1: Factors associated with child survival grouped into distal and proximate/intermediate variables.**

| **Group** | **Factor** | **Rationale** |
| --- | --- | --- |
|  | **Distal Factors** | |
| **Governmental** | **GDP per capita** | GDP a measure of the standard of living or the level of economic development and is indirectly associated with U5M [14,15]. It increases the share of resources allocated for food, housing, medical, public health services, education and health-related research which is likely to reduce U5M [15]. In the 1990s, a 10% increase in GDP was linked to a 4% reduction in child mortality [16]. |
|  | **Government healthcare spending (GHS)** | GHS is associated with U5M for up to 5 years after changes in GHS [17] and affects U5M through health and education services [18]. For example, pay cuts to healthcare workers, lower hospital budgets, reduced amounts spent on interventions and medical equipment, an increase of out-of-pocket payments, introduction of user fees and non-price rationing such as an increase in waiting times [18–20] impacts U5M negatively [17]. |
|  | **Governance** | Public health resources are fewer compared to health sector needs and requires prudent management and governance. However, in the presence of poor governance, there are leakages in public resources which results in undesired outcomes such as poor child education and health [21]. |
|  | **Health systems** | The functioning of a health system plays a significant role in child survival. It entails legislation, health financing, workforce, infrastructure, commodities, service delivery and health information systems. Gaps in performance in any of these indicators might result in adverse outcomes for child health resulting in higher U5M [22,23]. |
| **Societal** | **Conflict** | Child survival is affected negatively when there is conflict within and between communities. Food production is interrupted, and the population is displaced which results in internally displaced persons increasing food insecurity. Health facilities are destroyed affecting healthcare access for preventive and curative treatment [24,25]. Conflicts areas are often deprived of basic access to clean water and proper sanitation. Being born in an armed conflict zone has a 5·2 per 1000 births higher risk of dying than being born in a non-conflict by age one [26]. |
|  | **Ethnicity, religion and cultural beliefs** | Community norms, perceptions and belief’s related to disease might be antagonistic to western biomedical theory resulting in adverse effects on a child’s growth and survival [10]. For example, the probability of deaths before age two, was high in the Luo community compared to Kikuyus in Kenya and 40% higher among the Hutu when compared to Tutsi in Rwanda [27]. Similar differences have been observed in Cameroon, Mali, and Senegal [28]. |
| **Environmental** | **Urban/Rural** **residency** | Urban areas have a lower probability of infectious disease contraction because they are associated with better living conditions (e.g. healthcare access) compared to rural areas [Balk et al., 2003]. Higher population density in the urban areas facilitates sharing of information and resources related to health [29]. However, urban slums are associated with increased disease risks due to poor access to health services, inadequate sanitation and hygiene, which might lead to disease [30]. Slums are also overcrowded (Item 8) |
|  | **Crowding and Population density** | In highly populated and overcrowded areas, disease transmissions rate is high thus higher U5M. Healthcare services in the slum areas are inadequate, sanitation is poor and access to safe and clean water is inadequate which results in higher U5M compared to non-slum areas [10]. |
|  | **Rainfall and Vegetation index** | Precipitation and vegetation density are associated with a higher prevalence of vector-borne diseases such as malaria (which result in U5M) due to favourable conditions created for disease vectors. Inadequate rainfall is associated with lower agricultural productivity leading to increases in undernutrition. In addition, in arid areas, water scarcity due to lack of rain may lead to poor sanitation [10,31,32]. |
| **Maternal** | **Education and literacy** | Increased education levels are associated with reduced U5M levels through better use of health services, financial advantages, autonomy and reduced fertility [33–37]. For example, a one year increase in mother’s education was associated with a 7-9% reduction in U5M in developing countries [37] and U5M was 58% lower among mothers with at least seven years of schooling compared to those without education [38]. About 51% of under-five lives were saved between 1970 and 2009 due to increased maternal educational [33]. |
|  | **Autonomy (female-headed households)** | Women’s autonomy influences the health and mortality of their children through decisions on family planning, health care seeking, pregnancy care and child-rearing [39,40]. A higher level of women empowerment is associated with lower fertility, longer birth interval and lower rates of unintended pregnancy [41]. However, in some cultures, women living in rural areas, are subordinate to men and considered ‘inferior’ to men. Women are often disadvantaged compared to men in terms of their access to assets, employment, health care, and education. Thus female-headed households are often poorer than male-headed households [42]. |
|  | **Short birth**  **spacing** | Short birth intervals may drain the reproductive and nutritional resources of the mother resulting in premature and weaker births. Births spaced closely together compete for fewer resources, and at a higher likelihood of transmitting infections exists [34,43,44]. Childhood mortality decreases with an increasing birth interval up to 36 months where it plateaus [45]. |
|  | **Family planning** | Family planning practices can reduce U5M because births that occur within short birth intervals and the extremes of parity or maternal age are at a higher risk of death or obstetric complications [46]. Thus, family planning averts closely spaced births and any ill-timed births [34,43,44]. It facilitates better-informed choices on the time of birth, the period between two births, and the (maximum) number of children a couple wants [47]. |
|  | **Parity** | High parity (number of children a mother has ever had) is associated with high U5M. This is because there are high physical and caloric demands for repeated pregnancy that leads to maternal depletion syndrome and linked to shorter birth intervals. High parity may be associated with more inferior nutritional status due to lesser parental investment and competition of finite resources between siblings [48,49]. |
| **Child** | **Nutrition status:** | Undernutrition predisposes children to high risk of chronic diseases leading to childhood mortality [50–52]. It comprises of wasting (low weight-for-height- acute weight loss), stunting (low height-for-age), underweight (low weight compared with that expected for a well-nourished child of that age and sex) and deficiencies of essential micronutrients (vitamins and minerals), obesity and over-consumption of specific nutrients [50]. Stunting or chronic undernutrition develops over a long period due to limited access to food, health and care and is associated with cognitive impairments. Acute food shortages trigger wasting or acute malnutrition and are characterized by a rapid deterioration of nutritional status over a short period. About 56% of children (6-59 months) deaths could be attributed to malnutrition with 83% of these being due to mild-moderate malnutrition [52]. |
|  | **Breastfeeding**: | Breast milk has immunological properties that offer protection against diarrhoea and common childhood illnesses and has all nutritional requirements for up to six months and is sterile. It can extend the postpartum anovulation period, thus, a larger birth interval [53]. The WHO recommends exclusive breastfeeding of infants up to six months after birth, followed by nutritious complementary foods with continued breastfeeding up to 24 months [54,55]. Improved breastfeeding practices can prevent 823,000 annual deaths in under-fives. This is in addition to lower intelligence associated with non-breastfeeding, and economic loses of 0·49% of world gross national income [54,56]. |
|  | **Low birth weight (LBW):** | LBW (< 2,500 grams) is associated with increased foetal, neonatal, post-neonatal, infant, childhood mortality and morbidity [57]. About 14% of infants weigh <2500 g at birth in low-middle income countries (LMICs) [58,59]. In rural Ghana, infants born with LBW were two times more likely to die in infancy compared to those with average birth weight [58]. The global target is a 30% reduction of LBW by 2025 based on 2012 values [60]. |
| **Household** | **Wealth/ socio-economic status:** | Children belonging to households of lower socioeconomic status have been associated with higher U5M [61]. Poorer socioeconomic classes might be more prone to traits that do not promote better health, e.g. open defecation and often associated with poor housing, sanitation, less access to health-enhancing interventions constrained by money and longer travel time to facilities [62]. |
|  | **Water and sanitation:** | Diarrheal diseases are a major cause of U5M [63,64]. Key to its control is hygiene, access to safe and clean water and sanitation. Water is classified as poor quality if it is based on surface water (rivers, lakes or standing rainwater), intermediate if it is abstracted below the surface (standpipes, springs, wells and boreholes) and improved if its piped water. Similarly, sanitation is subdivided into poor (no access to any toilet facilities), intermediate (any toilet except flush) and improved (flush toilet). Improved sanitation and water access have been associated with a lower risk of mortality, diarrhoea and stunting [65,66]. |
| **Infections** | **Human immune-deficiency virus (HIV)** | Child survival is influenced negatively by HIV/AIDS. Children can acquire the virus through vertical transmission (about 25–30%) if they are born of HIV-seropositive mothers [67]. The mother-to-child transmission can occur before, during or after delivery [68]. Most of these children die before age five with HIV prevalence [67]. Additionally, many resources are needed to ensure health and survival among the HIV seropositive affecting those that are not infected [67]. |
|  | **Malaria** | Malaria is a risk factor of U5M that results in death through direct and indirect pathways [69]. In addition to being a direct cause of death, malaria infection increases the risk of severe anaemia in pregnant women, LBW, growth retardation in childhood [70–74], consequential infections such as adverse reactions to treatment, neurologic disabilities, cognitive impairment [75,76], enhanced severity of disease through suppression of the immune system all which can lead to death before age five [77]. |
| **Access to healthcare** | **Access to healthcare** | Healthcare access involves availability of resources, acceptability of the healthcare systems, accommodation of population demands, ability to meet financial obligations, and geographical accessibility [78–84]. It facilitates interaction of the population in need (children and pregnant women) with lifesaving interventions and treatment that prevents mortality [85–88]. In LMICs, higher odds of childhood mortality are associated with living further away from a health facility [87,89]. |
| **Healthcare utilization** | **Antenatal care (ANC)** | There are several interventions (e.g. Tetanus toxoid vaccination) and services given during pregnancy, labour, delivery, and post-natal period during ANC from a skilled health professional meant to reduce morbidity and mortality. These services play an essential role in ensuring a healthy mother and baby during pregnancy and after delivery through risk identification, prevention and management of pregnancy-related or concurrent diseases and health education and promotion [90]. Until 2016, the WHO recommended at least 4 ANC visits [91] which were revised to ≥8 in 2016 [92,93]. |
|  | **Skilled birth attendant (SBA)** | When health workers with midwifery training provide delivery care, pregnancy-related complications can be detected early which facilitates the provision of timely lifesaving interventions such as emergency obstetric services. An insufficient number of skilled health workers, weak healthcare systems, poor services, cultural beliefs, cost, access, and lack of women autonomy are associated with low SBA [94]. |
|  | **Place of delivery** | The place of delivery determines the quality of care received by the mother and the infant. Delivery at a health facility reduces deaths that might arise due to pregnancy-related complications, through a sanitary environment and medically correct birth assistance or referral services [95]. Health facility delivery can reduce neonatal mortality by up to 29% [94,96]. |
|  | **Health seeking behaviour** | Timeliness in seeking treatment facilitates prompt treatment, early diagnosis and prevents progression of the disease to severe levels. Different markers of treatment-seeking behaviour can be used. Treatment-seeking for fever is often used because fever is a significant symptom of malaria and other acute infections in children such as pneumonia. In addition, seeking diarrhoea treatment is also used as a marker of health-seeking behaviour. Low access to seeking care could be due to reasons such as affordability and availability. |
| **Child health interventions** | **Immunization** | Immunizations reduce U5M significantly [29,97] E.g. Between two and three million child deaths are averted per year when children are vaccinated against diphtheria, tetanus, pertussis and measles [97–100]. The WHO established the expanded programme on immunization in 1974 which introduced vaccines to target tuberculosis, polio, diphtheria, pertussis, tetanus, and measles [101]. Over time other vaccines such as yellow fever, hepatitis B, Hib, and pneumococcal conjugate were introduced [100,101]. The global target aims for a vaccination coverage of at least 90% nationally and at least 80% coverage subnational [102]. |
|  | **Children supplements** | Micronutrient deficiency is associated with childhood morbidity and mortality [103]. Micronutrients can be sourced from foods or through supplements. For example, the deficiency of vitamin A is the leading cause of blindness in undernourished children, contributes to morbidity and mortality due to diarrheal diseases and measles [104]. Other micronutrients include iron, zinc, folate and iodine. |
|  | **ITN and ACT use** | Different preventive measures have been recommended to reduce mortality and morbidity due to malaria. Key among the interventions include the use of insecticide-treated bed net (ITNs) and prompt treatment of clinical malaria cases with artemisinin-based combination therapy (ACT). About 68% of the decline in malaria risk from 1990 to 2015 is thought to be due to ITNs and 19% due to ACTs in sub-Saharan Africa [105]. |
|  | **Oral Rehydration Salts (ORS)** | ORS is used to treat diarrhoea by reducing severe dehydration thus mitigate against morbidity and mortality. Safe drinking water, adequate sanitation and hygiene are preventive measures against diarrhoea [106]. |
| **Maternal interventions** | **Tetanus toxoid (TT)** | Neonatal tetanus is associated with infant mortality especially in LMICs where a substantial number of deliveries take place at home or at settings where hygienic conditions may be poor. It is given to women during pregnancy to prevent neonatal tetanus which accounted for about 7% of IMR globally in 2000 [107,108]. The odds of neonatal deaths were reduced by 0.46 following more than one dose of TT vaccine [107]. At least two doses of TT vaccine are recommended during pregnancy for full protection [109]. |
|  | **Intermittent preventive treatment in pregnancy (IPTp)** | IPTp of malaria in pregnancy entails antimalarial medicine given to pregnant women during ANC visits, regardless of whether the recipient is infected with malaria or not [110]. It involves treatment with sulfadoxine-pyrimethamine in all areas with moderate to high malaria transmission in SSA. It reduces maternal malaria episodes, anaemia, placental parasitemia, LBW, and neonatal mortality [110]. In Mozambique, it reduced neonatal mortality by 61% in a trial setting [111]. |
|  | **Iron and Vitamin A** | Micronutrient intake (vitamins and iron) by women benefits both women and their children. Iron given during pregnancy protects the mother and foetus against anaemia, a major cause of perinatal and maternal mortality and an increased risk of preterm births and LBW [112]. Vitamin A is only recommended in areas where it is a severe public health problem to prevent night blindness [113]. |

**Section 3: Factors associated with child survival including their definition and source of data.**

| Group | ID | Variable | Definition | Datasets in reference to Table 1 | Time |
| --- | --- | --- | --- | --- | --- |
| Environmental factors | 1 | Rural residency | The proportion of households in rural areas | All census, DHS, WMS, MICS, AIS, KIHBS and MIS | Year of Survey |
|  | 2 | Precipitation | Annual average precipitation by county between 1990 and 2015 | Tropical Rainfall Measuring Mission Multi-Satellite and weather station data. | - |
|  | 3 | Enhanced vegetation index (EVI) | A vegetation index at 250 spatial resolution averaged to the county level | MODIS -Moderate Resolution Imaging Spectroradiometer- | - |
| Maternal Factors | 4 | Maternal education | The proportion of mothers (15-49 years) who had less than primary education at the time of the survey | All DHS, MICS, MIS and KIHBS; WMS 1997, AIS 2007; Census 1989,1999 | Year of Survey |
|  | 5 | Maternal literacy | The proportion of mothers (15-49 years) who can read all or parts of a sentence provided at the time of the survey | All DHS, MIS and KIHBS; WMS 1997; MICS 2007, 2008, 2011, 2013 | Year of Survey |
|  | 6 | Female-headed Households | The proportion of households headed by a female at the time of survey | All census, DHS, WMS, MICS, AIS and KIHBS | Year of Survey |
|  | 7 | Short birth spacing | The proportion of children with a preceding or succeeding birth interval <24 months | All DHS | Previous 5 years |
|  | 8 | Use of modern contraceptives | The proportion of women aged 15 to 49 years using any form of modern contraceptive at the time of the survey | All DHS; MICS 2007, 2008, 2011, 2013; WMS 1994 | Year of survey |
|  | 9 | High parity | The proportion of women aged, <30 years with 3 or more children or aged >29 years with 5 or more children | All Census, DHS and MICS; MIS 2015, AIS 2007 |  |
| Child factors | 10 | Underweight | The proportion of children whose weight for age was <=-2 standard deviations at the time of the survey | DHS 1993, 1998, 2003, 2014; MICS 2007,2011; All WMS; KIHBS 2015.  The estimates were computed based on the WHO 2006 reference for a well-nourished population |  |
|  | 11 | Wasted | The proportion of children whose weight for height was <=-2 standard deviations at the time of the survey |  |  |
|  | 12 | Stunted | The proportion of children whose height for age was <=-2 standard deviations at the time of the survey |  |  |
|  | 13 | Breastfed within the first hour of birth | The proportion of last born <5 years who were breastfed within the first hour after birth | DHS 1993, 1998, 2003 2008, 2014; MICS 2007, 2013; KIBHS 2015 | Previous 5 years for DHS, WMS and KIHBS  Previous 2 years for MICS |
|  | 14 | Exclusive breastfeeding | The proportion of lastborn living with the mother and breastfed up to six months at the time of the survey | DHS 1993, 1998, 2003, 2008, 2014; MICS 2007, 2008, 2011, 2013; All KIHBS |  |
|  | 15 | Continued breastfeeding | The proportion of children 6-18 months old who were still being breastfed at the time of the survey. | DHS 1993, 1998, 2003, 2008, 2014; All WMS, MICS and KIHBS |  |
|  | 16 | Low Birthweight (LBW) | The proportion of children weighing < 2500g at birth at the time of the survey among those whose weight was taken | DHS 1993, 1998, 2003, 2008, 2014  MICS 2000, 2011, 2008, 2013: All WMS |  |
| Household factors | 17 | Poor household | The proportion of households classified as poor or poorer by wealth index constructed using principal component analysis | DHS 1993, 1998, 2003, 2008, 2014; Census 1999,2009; All MICS, MIS and AIS | Year of survey |
|  | 18 | Improved Sanitation | The proportion of households who have access to flush toilet facilities | All Census, DHS, WMS, MICS, AIS, KIHBS and MIS |  |
|  | 19 | Access to any form of a toilet | The proportion of households who have access to any form of a toilet facility (improved and intermediate) |  |  |
|  | 10 | Improved water | The proportion of households who have access to piped water for drinking |  |  |
|  | 21 | Access to wells borehole and piped water | The proportion of households who have access to either piped (improved water) or boreholes/wells water for drinking (Intermediate) |  |  |
| Infections | 22 | HIV infection prevalence | The proportion of adults aged 15-49 who were HIV positive during the time of the survey | [114] | Year of survey |
|  | 23 | Malaria infection prevalence | The proportion of children aged 2-10 years with *Plasmodium falciparum* parasite in their blood. | [115] | Year of survey |
| Healthcare Utilization | 24 | At least one antenatal care visit (ANC1) | The proportion of women (15-49) who attended at least one ANC visit by a skilled provider (doctor nurse or midwife) during their last pregnancy in the last three years from the survey time | DHS 1993, 1998, 2003, 2008, 2014; MICS 2007, 2008, 2011, 2013; All AIS and MIS | Previous 5 years for all surveys expect MICS (2 year) |
|  | 25 | At least four antenatal care visits (ANC4) | The proportion of women (15-49) who attended four or more ANC visit during their last pregnancy in the last three years from survey time | DHS 1993, 1998, 2003, 2008, 2014; MICS 2008, 2011, 2013 AIS 2012; MIS 2015 |  |
|  | 26 | Skilled birth attendance (SBA) | Proportion of mothers who were attended by a doctor, nurse, midwife or a community health worker during delivery | All DHS, WMS and MICS; AIS 2012; KIBHS 2015 |  |
|  | 27 | Health facility deliveries (HFD) | Proportion of mothers who delivered at a health facility | All DHS, WMS; KIBHS and MICS; AIS 2012 |  |
|  | 28 | Diarrhoea treatment-seeking | The proportion of children <5 years who had diarrhoea within the past two weeks before the survey whose treatment was sought from a medical facility | All DHS; MICS 2007, 2008, 2011,2013 | Year of survey |
|  | 29 | Fever/cough treatment-seeking | The proportion of children <5 years who had fever/cough within the past two weeks before the survey whose treatment was sought from a health provider | All DHS and MICS; MIS 2015 |  |
| Child health interventions | 30 | Bacille Calmette–Guérin (BCG) | Proportion of children alive at the time of the survey aged 12-23 months who received BCG vaccine | All DHS, WMS and MICS; KIHBS 2015. | Previous 2 years for all surveys (12-24 months old) |
|  | 31 | Three diphtheria–tetanus–pertussis vaccinations (DPT3) | The proportion of children alive at the time of the survey aged 12-23 months who received up to the third dose of DPT vaccine |  |  |
|  | 32 | Three doses of Polio (Polio3) | The proportion of children alive at the time of the survey aged 12-23 months who received up to the third dose of Polio vaccine |  |  |
|  | 33 | Measles | Proportion of children alive at the time of the survey aged 12-23 months who received measles vaccine |  |  |
|  | 34 | Fully immunized | The proportion of children aged 12-23 months, alive at the time of the survey who received Polio 3, DPT3, BCG and measles vaccines |  |  |
|  | 35 | Oral rehydration salts (ORS use) | Proportion of children <5 years old who had diarrhoea within the past two weeks before the survey who received ORS treatment | All DHS, MICS and KIHBS | Year of survey |
|  | 36 | Vitamin A- children | The proportion of children 6-59 months old who received Vitamin A supplement within six months before the survey | DHS 2003, 2008, 2014; All MICS |  |
|  | 37 | Insecticide-treated bed nets (ITN) use by children | The proportion of children <5 years who slept under an ITN in the previous night before the survey | DHS 2003, 2008, 2014, KIHBS 2005; All MIS |  |
|  | 38 | Recommended antimalarial use | Proportion of children <5 old who had fever within the past two weeks before the survey who received the recommended antimalarial drugs among those given any drug for fever | DHS 2003, 2008, 2014; All MIS and MICS |  |
| Maternal health interventions | 39 | Tetanus toxoid injection | Proportion of women who received the recommended doses (2) of tetanus toxoid injection during their last pregnancy up to three years since data collection. | DHS 1993, 1998, 2003, 2008, 2014 | Previous 5 years.  **Note*** The factors with a recall period of 5 years preceding the survey were limited to three years due to errors and bias associated with a longer recall period [116] |
|  | 40 | Intermittent preventive treatment in pregnancy (IPTp 1) | Percentage of women (15-49 years) with live birth, three years preceding a survey who during the last pregnancy received at least one dose of SP/Fansidar | DHS 2003, 2008, 2014; All MIS |  |
|  | 41 | IPTp 2 | Percentage of women (15-49 years) with a live birth in three years preceding the survey who, during the last pregnancy, received two doses of SP/Fansidar at least one of which was received during an ANC visit |  |  |
|  | 42 | Iron supplement | The proportion of mothers who took iron tablets/syrup during their last pregnancy | DHS 2003, 2008, 2014 |  |
|  | 43 | Vitamin A-mothers | Proportion of mothers who received vitamin A supplement within 2 months after delivery |  |  |

**References**

1 Hill K. Frameworks for studying the determinants of child survival. *Bull World Health Organ* 2003;**81**:138–9.

2 Corsi DJ, Subramanian S V. Association between coverage of maternal and child health interventions, and under-5 mortality: a repeated cross-sectional analysis of 35 sub-Saharan African countries. *Glob Health Action* 2014;**7**:24765. doi:10.3402/gha.v7.24765

3 Mosley WH, Chen CL. An Analytical Framework for the Study of Child Survival in Developing Countries. *Popul Dev Rev* 1984;**81**:25–45.

4 Schell CO, Reilly M, Rosling H, *et al.* Socioeconomic determinants of infant mortality: a worldwide study of 152 low-, middle-, and high-income countries. *Scand J Public Health* 2007;**35**:288–97. doi:10.1080/14034940600979171

5 Liang S, Macinko J, Yue D, *et al.* The impact of the health care workforce on under-five mortality in rural China. *Hum Resour Health* 2019;**17**:21. doi:10.1186/s12960-019-0357-5

6 Mammo A. Factors responsible for childhood mortality variation in rural Ethiopia. *J Biosoc Sci* 1993;**25**:223–38. doi:10.1017/S0021932000020514

7 Boco AG. Individual and Community-level Effects on Child Mortality: An Analysis of 28 Demographic and Health Surveys in Sub-Saharan Africa. DHS Work. Pap. 2010;:1–86.http://www.measuredhs.com/pubs/pdf/WP73/WP73.pdf (accessed 17 Sep 2017).

8 Schultz TP. Studying the impact of household economic and community variables on child mortality. *Popul Dev Rev Suppl Surviv Strateg Res* 1984;**10**:215–35. doi:10.2307/2807962

9 Balk D, Pullum T, Storeygard A, *et al.* A spatial analysis of childhood mortality in West Africa. *Popul Space Place* 2004;**10**:175–216. doi:10.1002/psp.328

10 Balk D, Pullum T, Storeygard A, *et al.* Spatial Analysis of Childhood Mortality in West AFrica. DHS Geogr. Stud. 2003;:60.https://dhsprogram.com/pubs/pdf/SAR5/GS1.pdf (accessed 20 Sep 2017).

11 Roberts DA, Ng M, Ikilezi G, *et al.* Benchmarking health system performance across regions in Uganda: a systematic analysis of levels and trends in key maternal and child health interventions, 1990–2011. *BMC Med* 2015;**13**:285. doi:10.1186/s12916-015-0518-x

12 Wollum A, Burstein R, Fullman N, *et al.* Benchmarking health system performance across states in Nigeria: A systematic analysis of levels and trends in key maternal and child health interventions and outcomes, 2000-2013. *BMC Med* 2015;**13**:208. doi:10.1186/s12916-015-0438-9

13 Colson KE, Dwyer-Lindgren L, Achoki T, *et al.* Benchmarking health system performance across districts in Zambia: a systematic analysis of levels and trends in key maternal and child health interventions from 1990 to 2010. *BMC Med* 2015;**13**:69. doi:10.1186/s12916-015-0308-5

14 O’Hare B, Makuta I, Chiwaula L, *et al.* Income and child mortality in developing countries: A systematic review and meta-analysis. *J R Soc Med* 2013;**106**:408–14. doi:10.1177/0141076813489680

15 Preston SH. The Changing Relation between Mortality and level of Economic Development. *Popul Stud (NY)* 1975;**29**:231–48. doi:10.1080/00324728.1975.10410201

16 Pritchett L, Summers L. Wealthier is Healthier. *J Hum Resour* 1996;**31**:841–68.

17 Maruthappu M, Ng KYB, Williams C, *et al.* Government Health Care Spending and Child Mortality. *Pediatrics* 2015;**135**:e887–94. doi:10.1542/peds.2014-1600

18 Horton R. The global financial crisis: an acute threat to health. *Lancet* 2009;**373**:355–6. doi:10.1016/s0140-6736(09)60116-1

19 Garenne M, Gakusi AE. Vulnerability and Resilience: Determinants of Under-Five Mortality Changes in Zambia. *World Dev* 2006;**34**:1765–87. doi:10.1016/j.worlddev.2006.02.005

20 Martin A, Lassman D, Whittle L, *et al.* Recession contributes to slowest annual rate of increase in health spending in five decades. *Health Aff* 2011;**30**:11–22. doi:10.1377/hlthaff.2010.1032

21 Hu B, Mendoza RU. Public Health Spending, Governance and Child Health Outcomes: Revisiting the Links. *J Hum Dev Capab* 2013;**14**:285–311. doi:10.1080/19452829.2013.765392

22 Keats EC, Ngugi A, Macharia W, *et al.* Progress and priorities for reproductive, maternal, newborn, and child health in Kenya: a Countdown to 2015 country case study. *Lancet Glob Heal* 2017;**5**:e782–95. doi:10.1016/S2214-109X(17)30246-2

23 Keats EC, Macharia W, Singh NS, *et al.* Accelerating Kenya’s progress to 2030: understanding the determinants of under-five mortality from 1990 to 2015. *BMJ Glob Heal* 2018;**3**:e000655. doi:10.1136/bmjgh-2017-000655

24 Kinyoki DK, Moloney GM, Uthman OA, *et al.* Conflict in Somalia: impact on child undernutrition. *BMJ Glob Heal* 2017;**2**:e000262. doi:10.1136/bmjgh-2016-000262

25 Egal F. Nutrition in conflict situations. *Br J Nutr* 2010;**96**:S17–9. doi:10.1079/bjn20061692

26 Burke M, Bhutta ZA, Black RE, *et al.* Armed conflict and child mortality in Africa: a geospatial analysis. *Lancet* 2018;**392**:857–65. doi:10.1016/s0140-6736(18)31437-5

27 Tabutin D, Akoto E. Socio-economic and cultural differentials in the mortality of sub-Saharan Africa. In: Walle tienne van de, Pison G, Sala-Diakanda M, eds. *Mortality and society in Sub-Saharan Africa*. Oxford, England: : Clarendon Press 1992.

28 Brockerhoff M, Hewett P. Inequality of child mortality among ethnic groups in sub- Saharan Africa. *Bull World Health Organ* 2000;**78**:30–41. doi:10.1590/S0042-96862000000100004

29 Defo BK. Determinants of infant and early childhood mortality in Cameroon: the role of socioeconomic factors, housing characteristics, and immunization status. *Soc Biol* 1994;**41**:181–211. doi:10.1080/19485565.1994.9988872

30 Kabaria CW. *Impacts of urbanization on malaria parasite prevalence in Africa*. 2015.

31 Balk D, Storeygard A, Levy M, *et al.* Child hunger in the developing world: An analysis of environmental and social correlates. *Food Policy* 2005;**30**:584–611. doi:10.1016/j.foodpol.2005.10.007

32 Webb P. Isolating Hunger: Reaching People in Need Beyond the Mainstream. 1998.https://www.wfp.org/sites/default/files/JointPaper.pdf (accessed 9 Sep 2017).

33 Gakidou E, Cowling K, Lozano R, *et al.* Increased educational attainment and its effect on child mortality in 175 countries between 1970 and 2009: A systematic analysis. *Lancet* 2010;**376**:959–74. doi:10.1016/S0140-6736(10)61257-3

34 Gupta M Das. Death Clustering , Mothers’ Education and the Determinants of Child Mortality in Rural Punjab , India. *Popul Stud (NY)* 2010;**44**:37–41.

35 Byhoff E, Hamati MC, Power R, *et al.* Increasing educational attainment and mortality reduction: a systematic review and taxonomy. *BMC Public Health* 2017;**17**:719. doi:10.1186/s12889-017-4754-1

36 Cadwell J. Mortality Decline An Examination of Nigerian Data. *Popul Stud (NY)* 1979;**33**:395–413.

37 Cleland JG, van Ginneken JK. Maternal education and child survival in developing countries: The search for pathways of influence. *Soc Sci Med* 1988;**27**:1357–68. doi:10.1016/0277-9536(88)90201-8

38 Hobcraft J. Women ’ s education , child welfare and child survival : a review of the evidence. *Heal Transit Rev* 1993;**3**:159–75. doi:10.2307/40652016

39 Doctor H V. Does living in a female-Headed household lower child mortality? The case of rural nigeria. *Rural Remote Health* 2011;**11**:1–14.

40 Adhikari R, Sawangdee Y. Influence of women’s autonomy on infant mortality in Nepal. *Reprod Health* 2011;**8**:7. doi:10.1186/1742-4755-8-7

41 Upadhyay UD, Gipson JD, Withers M, *et al.* Women’s empowerment and fertility: A review of the literature. *Soc Sci Med* 2014;**115**:111–20. doi:10.1016/j.socscimed.2014.06.014

42 Adhikari R, Podhisita C. Household headship and child death: Evidence from Nepal. *BMC Int Health Hum Rights* 2010;**10**:13. doi:10.1186/1472-698X-10-13

43 Forstet R. The Effects of Breastfeeding and Birth Spacing on Infant and Child Mortality in Bolivia *. 1994;**48**:497–511.

44 Manda SOM. Birth intervals, breastfeeding and determinants of childhood mortality in Malawi. *Soc Sci Med* 1999;**48**:301–12. doi:10.1016/S0277-9536(98)00359-1

45 Rutstein SO. Effects of preceding birth intervals on neonatal, infant and under-five years mortality and nutritional status in developing countries: Evidence from the demographic and health surveys. *Int J Gynecol Obstet* 2005;**89**:S7—S24. doi:10.1016/j.ijgo.2004.11.012

46 Bongaarts J. Data and Perspectives Does Family Planning Reduce Infant Mortality Rates ? *Popul Dev Rev* 1987;**13**:323–34.

47 Saha UR, Soest A van. Does Family Planning Reduce Infant Mortality? Evidence From Surveillance Data in Matlab, Bangladesh. Cent. Discuss. Pap. Ser. No. 2012-019. 2012. doi:10.2139/ssrn.2009853

48 Sonneveldt E, DeCormier Plosky W, Stover J. Linking high parity and maternal and child mortality: what is the impact of lower health services coverage among higher order births? *BMC Public Health* 2013;**13 Suppl 3**:S7. doi:10.1186/1471-2458-13-S3-S7

49 Hobcraft J, Mcdonald JW, Rutstein S, *et al.* Child-Spacing Effects on Infant and Early Child Mortality. *Popul Index* 1983;**49**:585–618.

50 Black RE, Allen LH, Bhutta ZA, *et al.* Maternal and child undernutrition: global and regional exposures and health consequences. *Lancet* 2008;**371**:243–60. doi:10.1016/S0140-6736(07)61690-0

51 Pelletier DL, Frongillo EA. Changes in Child Survival Are Strongly Associated with Changes in Malnutrition in Developing Countries. *J Nutr* 2018;**133**:107–19. doi:10.1093/jn/133.1.107

52 Pelletier DL, Frongillo EA, Schroeder DG, *et al.* The effects of malnutrition on child mortality in developing countries. *Bull World Health Organ* 1995;**73**:443–8. doi:10.1093/ije/dyr050

53 Huffman SL, Lamphere BB. Breastfeeding Performance and Child. *Popul Dev Rev* 1984;**10**:93–116.

54 Rollins NC, Bhandari N, Hajeebhoy N, *et al.* Why invest, and what it will take to improve breastfeeding practices? *Lancet* 2016;**387**:491–504. doi:10.1016/S0140-6736(15)01044-2

55 Michael K, Kakuma R. Optimal duration of exclusive breastfeeding ( Review ). *Cochrane Database ofSystematic Rev* 2012;**8**:1–3. doi:10.1002/14651858.CD003517.pub2.Copyright

56 Victora CG, Bahl R, Barros AJD, *et al.* Breastfeeding in the 21st century: Epidemiology, mechanisms, and lifelong effect. *Lancet* 2016;**387**:475–90. doi:10.1016/S0140-6736(15)01024-7

57 Maccormick MC. The Contribution of Low Birth Weight to Infant and Childhood mortality. *N Engl J Med* 1985;**312**:82–90.

58 O’Leary M, Edmond K, Floyd S, *et al.* A cohort study of low birth weight and health outcomes in the first year of life, Ghana. *Bull World Health Organ* 2017;**95**:574–83. doi:10.2471/BLT.16.180273

59 Lee ACC, Katz J, Blencowe H, *et al.* National and regional estimates of term and preterm babies born small for gestational age in 138 low-income and middle-income countries in 2010. *Lancet Glob Heal* 2013;**1**:e26–36. doi:10.1016/S2214-109X(13)70006-8

60 WHO. Global nutrition targets 2025: policy brief series (WHO/NMH/NHD/14.2). Policy Br. Ser. 2014. doi:10.2165/00024677-200302060-00002

61 Schoeps A, Souares A, Niamba L, *et al.* Childhood mortality and its association with household wealth in rural and semi-urban Burkina Faso. *Trans R Soc Trop Med Hyg* 2014;**108**:639–47. doi:10.1093/trstmh/tru124

62 Kutty VR, Thankappan KR, Kannan KP, *et al.* How Socioeconomic Status Affects Birth and Death Rates in Rural Kerala, India: Results of a Health Study. *Int J Heal Serv* 2005;**23**:373–86. doi:10.2190/9n4p-f1l2-13hm-cqvw

63 Boschi-Pinto C, Velebit L, Shibuya K. Estimating child mortality due to diarrhoea in developing countries. *Bull World Health Organ* 2008;**86**:710–7. doi:10.2471/BLT.07.050054

64 Anker R, Knowles JC. An Empirical Analysis of Mortality Differentials in Kenya at the Macro and Micro Levels. *Econ Dev Cult Change* 1980;**29**:165. doi:10.1086/451236

65 Fink G, Günther I, Hill K. The effect of water and sanitation on child health: Evidence from the demographic and health surveys 1986-2007. *Int J Epidemiol* 2011;**40**:1196–204. doi:10.1093/ije/dyr102

66 Victora CG, Smith PG, Vaughan JP, *et al.* Water supply, sanitation and housing in relation to the risk of infant mortality from diarrhoea. *Int J Epidemiol* 1988;**17**:651–4.http://ovidsp.ovid.com/ovidweb.cgi?T=JS&PAGE=reference&D=emed88&NEWS=N&AN=1988250841

67 Adetunji J. Trends in under-5 mortality rates and the HIV/AIDS epidemic. *Bull World Health Organ* 2000;**78**:1200–6.

68 Newell ML, Brahmbhatt H, Ghys PD. Child mortality and HIV infection in Africa: a review. *AIDS* 2004;**18**:S27–34. doi:10.1097/01.aids.0000125981.71657.0d

69 Snow RW, Korenromp EL, Gouws E. Pediatric mortality in Africa: Plasmodium falciparum malaria as a cause or risk? *Am J Trop Med Hyg* 2004;**71**:16–24. doi:http://dx.doi.org/10.1016/S1471-4922%2801%2902031-1

70 Brabin BJ. The risks and severity of malaria in pregnant women. 1991;**1**:1–33.http://apps.who.int/iris/bitstream/10665/61511/1/TDR_FIELDMAL_1.pdf (accessed 18 Oct 2016).

71 Guyatt H, Snow R. Malaria in pregnancy as an indirect cause of infant mortality in sub- Saharan Africa. *Trans R Soc Trop Med Hyg* 2001;**95**:569–76. doi:10.1016/S0035-9203(01)90082-3

72 McGregor IA. Epidemiology, malaria and pregnancy. *Am J Trop Med Hyg* 1984;**33**:517–25.

73 Desai M, ter Kuile FO, Nosten F, *et al.* Epidemiology and burden of malaria in pregnancy. *Lancet Infect Dis* 2007;**7**:93–104. doi:10.1016/S1473-3099(07)70021-X

74 Guyatt HL, Snow RWR. Impact of malaria during pregnancy on low birth weight in sub-Saharan Africa. *Clin Microbiol Rev* 2004;**17**:760–9. doi:10.1128/CMR.17.4.760

75 Holding PA, Snow RW. Impact of Plasmodium falciparum malaria on performance and learning: Review of the evidence. *Am J Trop Med Hyg* 2001;**64**:68–75.

76 Snow RW. The burden of malaria: Understanding the balance between immunity, public health and control. *J Med Microbiol* 2000;**49**:1053–5. doi:10.1099/0022-1317-49-12-1053

77 Molineaux L. Malaria and mortality: some epidemiological considerations. *Ann Trop Med Parasitol* 1997;**91**:811–25. doi:10.1080/00034989760572

78 Aday LA, Andersen R. A framework for the study of access to medical care. *Health Serv Res* 1974;**9**:208–20.

79 Penchansky R, Thomas JW. The concept of access: definition and relationship to consumer satisfaction. *Med Care* 1981;**19**:127–40.

80 Gautam S, Li Y, Johnson TG. Do alternative spatial healthcare access measures tell the same story? *GeoJournal* 2014;**79**:223–35. doi:10.1007/s10708-013-9483-0

81 Levesque J-F, Harris MF, Russell G. Patient-centred access to health care: conceptualising access at the interface of health systems and populations. *Int J Equity Health* 2013;**12**:18. doi:10.1186/1475-9276-12-18

82 Higgs G. A Literature Review of the Use of GIS-Based Measures of Access to Health Care Services. *Health Serv Outcomes Res Methodol* 2004;**5**:119–39. doi:10.1007/s10742-005-4304-7

83 Alun EJ, David RP. *Accesibility and Utilization : Geographical Perspectives on Health Care Delivery*. London: Harper & Row Ltd 1984.

84 Haas JS, Phillips KA, Sonneborn D, *et al.* Variation in access to health care for different racial/ethnic groups by the racial/ethnic composition of an individual’s county of residence. *Med Care* 2004;**42**:707–14.

85 Evans DB, Hsu J, Boerma T. Universal health coverage and universal access. *Bull World Health Organ* 2013;**91**:10–1. doi:10.2471/BLT.13.125450

86 Anthopolos R, Simmons R, O’Meara WP. A retrospective cohort study to quantify the contribution of health systems to child survival in Kenya: 1996–2014. *Sci Rep* 2017;**7**:44309. doi:10.1038/srep44309

87 Karra M, Fink G, Canning D. Facility distance and child mortality: a multi-country study of health facility access, service utilization, and child health outcomes. *Int J Epidemiol* 2016;**46**:817–826. doi:10.1093/ije/dyw062

88 Kadobera D, Sartorius B, Masanja H, *et al.* The effect of distance to formal health facility on childhood mortality in rural Tanzania, 2005-2007. *Glob Health Action* 2012;**5**:1–9. doi:10.3402/gha.v5i0.19099

89 Okwaraji YB, Edmond KM. Proximity to health services and child survival in low- and middle-income countries: a systematic review and meta-analysis. *BMJ Open* 2012;**2**:e001196. doi:10.1136/bmjopen-2012-001196

90 WHO. Integrated Management of Pregnancy and Childbirth. WHO Recommended Interventions for Improving Maternal and Newborn Health. 2009;:1–6.http://apps.who.int/iris/bitstream/10665/69509/1/WHO_MPS_07.05_eng.pdf (accessed 8 Sep 2016).

91 Villar J, Ba’aqeel H, Piaggio G, *et al.* WHO antenatal care randomised trial for the evaluation of a new model of routine antenatal care. *Lancet* 2001;**357**:1551–64. doi:10.1016/S0140-6736(00)04722-X

92 Islam MM, Masud MS. Determinants of frequency and contents of antenatal care visits in Bangladesh: Assessing the extent of compliance with the WHO recommendations. *PLoS One* 2018;**13**:e0204752. doi:10.1371/journal.pone.0204752

93 WHO. WHO recommendations on antenatal care for a positive pregnancy experience. 2016;:1–72.https://dl140.zlibcdn.com/download/article/17712983?token=58b55c86c54c84616cc8f4e37385de25 (accessed 22 Apr 2019).

94 Chinkhumba J, De Allegri M, Muula AS, *et al.* Maternal and perinatal mortality by place of delivery in sub-Saharan Africa: A meta-analysis of population-based cohort studies. *BMC Public Health* 2014;**14**:1–9. doi:10.1186/1471-2458-14-1014

95 Ajaari J. Impact Of Place Of Delivery On Neonatal Mortality In Rural Tanzania. *Int J MCH AIDS* 2012;**1**:49–59. doi:10.1016/j.jval.2013.03.1059

96 Tura G, Fantahun M, Worku A. The effect of health facility delivery on neonatal mortality: Systematic review and meta-analysis. *BMC Pregnancy Childbirth* 2013;**13**:1–9. doi:10.1186/1471-2393-13-18

97 Philippe D, Jean-Marie O-B, Marta G-D, *et al.* Global immunization: status, progress, challenges and future. *BMC Int Health Hum Rights* 2009;**9**:S2. doi:10.1186/1472-698X-9-S1-S2

98 Simons E, Mort M, Dabbagh A, *et al.* Strategic planning for measles control: Using data to inform optimal vaccination strategies. *J Infect Dis* 2011;**204**:S28-34. doi:10.1093/infdis/jir095

99 WHO, UNICEF, World Bank. State of the world`s vaccines and immunization. 2009;:1–208. doi:10.4161/hv.6.2.11326

100 Adeloye D, Jacobs W, Amuta AO, *et al.* Coverage and determinants of childhood immunization in Nigeria: A systematic review and meta-analysis. *Vaccine* 2017;**35**:2871–81. doi:10.1016/j.vaccine.2017.04.034

101 Wiysonge CS, Uthman OA, Ndumbe PM, *et al.* A bibliometric analysis of childhood immunization research productivity in Africa since the onset of the Expanded Program on Immunization in 1974. *BMC Med* 2013;**11**:66. doi:10.1186/1741-7015-11-66

102 WHO. Global Vaccine Action Plan Global Vaccine Action Plan 2011-2020. 2013. doi:10.1016/j.vaccine.2013.02.015

103 Martinez H. Vitamin A supplementation and child mortality. *Lancet* 1986;**328**:451–2. doi:10.1111/j.1744-6198.2000.tb01169.x

104 Rotondi MA, Khobzi N. Vitamin A supplementation and neonatal mortality in the developing world: a meta-regression of cluster-randomized trials. *Bull World Health Organ* 2010;**88**:697–702.

105 Bhatt S, Weiss DJ, Cameron E, *et al.* The effect of malaria control on Plasmodium falciparum in Africa between 2000 and 2015. *Nature* 2015;**526**:207–11. doi:10.1038/nature15535

106 WHO. Diarrhoeal disease. 2017.https://www.who.int/news-room/fact-sheets/detail/diarrhoeal-disease (accessed 3 May 2019).

107 Singh A, Pallikadavath S, Ogollah R, *et al.* Maternal Tetanus Toxoid Vaccination and Neonatal Mortality in Rural North India. *PLoS One* 2012;**7**:e48891. doi:10.1371/journal.pone.0048891

108 Lawn JE, Cousens S, Zupan J. 4 Million neonatal deaths: When? Where? Why? *Lancet* 2005;**365**:891–900. doi:10.1016/S0140-6736(05)71048-5

109 WHO. WHO recommendation on tetanus toxoid vaccination for pregnant women. WHO Reprod. Heal. Libr. 2016.https://extranet.who.int/rhl/topics/preconception-pregnancy-childbirth-and-postpartum-care/antenatal-care/who-recommendation-tetanus-toxoid-vaccination-pregnant-women (accessed 29 Apr 2019).

110 WHO. Intermittent preventive treatment in pregnancy (IPTp). 2018.https://www.who.int/malaria/areas/preventive_therapies/pregnancy/en/ (accessed 22 Apr 2018).

111 Menéndez C, Bardají A, Sigauque B, *et al.* Malaria prevention with IPTp during pregnancy reduces neonatal mortality. *PLoS One* 2010;**5**:e9438. doi:10.1371/journal.pone.0009438

112 Nisar YB, Dibley MJ. Iron/folic acid supplementation during pregnancy prevents neonatal and under-five mortality in Pakistan: propensity score matched sample from two Pakistan Demographic and Health Surveys. *Glob Health Action* 2016;**9**:29621.

113 McGuire S. WHO Guideline: Vitamin A Supplementation in Pregnant and Postpartum Women. *Adv Nutr* 2012;**3**:215–6. doi:10.3945/an.111.001701

114 NACC. Kenya HIV prevalence estimates. 2020.https://nacc.or.ke/ (accessed 22 Oct 2020).

115 Macharia PM, Giorgi E, Noor AM, *et al.* Spatio-temporal analysis of Plasmodium falciparum prevalence to understand the past and chart the future of malaria control in Kenya. *Malar J* 2018;**17**:340. doi:10.1186/s12936-018-2489-9

116 Ngandu NK, Manda S, Besada D, *et al.* Does adjusting for recall in trend analysis affect coverage estimates for maternal and child health indicators? An analysis of DHS and MICS survey data. *Glob Health Action* 2016;**9**:32408. doi:10.3402/GHA.V9.32408
