# Supplementary material for: The impact of child health interventions and risk factors on child survival in Kenya, 1993–2014: a Bayesian spatio-temporal analysis with counterfactual scenarios
Source: BMC Med. 2021 May 4;19:102. doi: 10.1186/s12916-021-01974-x (PMC8094495; doi:10.1186/s12916-021-01974-x)
Supplement: Supplementary file 2 — Additional file 2. Model development framework to select a set of factors significantly associated with child survival in Kenya between 1993 and 2014 (Section 1) and model diagnostics (Section 2). [file 12916_2021_1974_MOESM2_ESM.docx]

# Additional File 2

# Section 1: Model development

Factors whose impact is captured by other variables under consideration were excluded to reduce circularity, collinearity and confounding in the counterfactual model [1–3]. The effect of antimalarial medicine and insecticide-treated bed nets (ITNs) use is captured by malaria infection prevalence [4–7]. Intermittent preventive treatment in pregnancy (IPTp) is recommended only in 13 malaria-endemic counties [8] and administered to prevent malaria in pregnancy which results in low birth weight (LBW) [9–12]. Vaccination coverage was modelled for three doses of diphtheria, pertussis, and tetanus (DPT3) and Polio (Polio3), measles and BCG including their composite called fully immunized status [13]. Consequently, factors on the use of ITNs, antimalarials, IPTp, DPT3, Polio3, measles and BCG were excluded [1–3].

Similarly, factors with overlapping roles( measuring the same thing) (e.g. ANC1 and ANC4; maternal literacy and maternal education) were grouped [1–3] (Table 1). Following previous approaches [2,3], the relationship between U5M and each factor per group was explored by fitting a univariable regression model. The models in each group were compared via the Akaike Information Criterion (AIC) and the factor with the lowest AIC per group was included (Table 1).

Table 1: Regression coefficients and AIC from the simple linear regression models including the retained (green) and excluded (orange) factors. The correlation coefficient (r) is also shown

| **ID** | **Group** | **Determinant** | **coefficient and 95% CI** | **AIC** | **r** |
| --- | --- | --- | --- | --- | --- |
| 1 | Environment | Rainfall | 0.00028 [0.0002-0.0003] | 1003.6 | 0.80 |
|  |  | Enhanced Vegetation Index | 0.87071 [0.5763-1.1651] | 1129.6 |  |
| 2 | Health education | Maternal education | 0.49469 [0.369-0.6204] | 1104.7 | 0.88 |
|  |  | Maternal literacy | -0.02359 [-0.1197-0.0725] | 1162.6 |  |
| 3 | Fertility | Short birth spacing | 2.45362 [2.0407-2.8666] | 1034.9 | 0.66 |
|  |  | High Parity | 1.81663 [1.5494-2.0839] | 998.3 |  |
| 4 | Access to sanitation | Improved sanitation | -0.22875 [-0.5157-0.0582] | 1160.4 | 0.25 |
|  |  | Better sanitation | -0.23623 [-0.3352--0.1373] | 1141.1 |  |
| 5 | Access to clean water | Improved water | -0.52163 [-0.6487--0.3946] | 1099.7 | 0.79 |
|  |  | Better water | -0.37742 [-0.5117--0.2431] | 1132.8 |  |
| 6 | Antenatal care | ANC1 | -0.15096 [-0.4137-0.1118] | 1161.5 | 0.70 |
|  |  | ANC4 | 0.66412 [0.4167-0.9116] | 1135.4 |  |
| 7 | Institutional care | Skilled Birth attendance | -0.601 [-0.7301--0.4719] | 1082.4 | 0.96 |
|  |  | Health facility births | -0.64412 [0.7799--0.5083] | 1079.5 |  |

**Simple linear regression**

Previous studies have shown all the factors under consideration to be associated with U5M (Additional file 2). To validate these findings against the Kenyan context, a univariable regression model was fitted between U5M and each of the 28 factors retained in the preceding stage. Factors with a p-value > 0.2 coloured orange in Table 2 were excluded.

Table 2: Regression coefficients from univariate models assessing the association between 28 factors and U5M. Factors not statistically significant are coloured orange.

| **ID** | **Variable** | **Estimate [95% CI]** | **P value** | **AIC** |
| --- | --- | --- | --- | --- |
| 1 | Rural residence | 0.1534 [0.0238-0.283] | 0.0200 | 1157.4 |
| 2 | Precipitation | 0.0003 [0.0002-0.0003] | <0.0001 | 1003.6 |
| 3 | EVI | - | - | - |
| 4 | Maternal education | 0.4947 [0.369-0.6204] | <0.0001 | 1104.7 |
| 5 | Maternal literacy | - |  |  |
| 6 | Female-headed Households | 0.8596 [0.4827-1.2364] | <0.0001 | 1142.9 |
| 7 | Short birth spacing | - | - | - |
| 8 | Use of modern contraceptives | -1.0063 [-1.2013--0.8113] | <0.0001 | 1064.9 |
| 9 | High parity | 1.81663 [1.5494-2.0839] | <0.0001 | 998.3 |
| 10 | Underweight | 0.52262 [0.1235-0.9218] | <0.0001 | 1156.2 |
| 11 | Wasted | -0.32379 [-0.8976-0.25] | 0.2680 | 1161.6 |
| 12 | Stunted | 2.30384 [1.8417-2.766] | <0.0001 | 1071.1 |
| 13 | Breastfed within first hour of birth | -1.9575 [-2.1256--1.7894] | <0.0001 | 739.5 |
| 14 | Exclusive breastfeeding | 0.0357 [-0.2124-0.2838] | 0.7780 | 1162.7 |
| 15 | Continued breastfeeding | -0.71724 [-1.8098-0.3753] | 0.1980 | 1161.1 |
| 16 | Low Birthweight | 0.4821 [-2.2758-3.24] | 0.7320 | 1162.7 |
| 17 | Poor household | 0.74312 [0.6122-0.874] | <0.0001 | 1045.3 |
| 18 | Improved Sanitation | - | - | - |
| 19 | Better sanitation | -0.2362 [-0.3352--0.1373] | <0.0001 | 1141.1 |
| 20 | Improved water | -0.5217 [-0.6487--0.3946] | <0.0001 | 1099.7 |
| 21 | Access better water | - |  |  |
| 22 | HIV | 2.0026 [1.883-2.1222] | <0.0001 | 422.7 |
| 23 | Malaria | 6.0134 [5.5712-6.4556] | <0.0001 | 620.2 |
| 24 | ANC1 | - | - | - |
| 25 | ANC4 | 0.6641 [0.4167-0.9116] | <0.0001 | 1135.4 |
| 26 | Skilled birth attendance | - | - | - |
| 27 | Health facility births | -0.64412[0.7799--0.5083] | <0.0001 | 1079.5 |
| 28 | Diarrhoea treatment-seeking | -0.6909 [-1.0585--0.3231] | <0.0001 | 1149.3 |
| 29 | Fever/cough treatment-seeking | -2.94264 [-3.2902--2.595] | <0.0001 | 917.8 |
| 30 | BCG | - | - | - |
| 31 | DPT3 | - | - | - |
| 32 | Polio3 | - | - | - |
| 33 | Measles | - | - | - |
| 34 | Fully immunized | -0.8220 [-0.9825--0.6615] | <0.0001 | 1066.3 |
| 35 | ORS use | 0.01009 [-0.4586-0.4787] | 0.9660 | 1162.8 |
| 36 | Vitamin A- children | -0.5900 [-0.7078--0.472] | <0.0001 | 1070.5 |
| 37 | ITN use by children | - | - | - |
| 38 | Antimalarial use | - | - | - |
| 39 | Tetanus toxoid injection | -1.60765 [-2.0192--1.1961] | 0.0000 | 1105.6 |
| 40 | IPTp 1 | - | - | - |
| 41 | IPTp 2 | - | - | - |
| 42 | Iron supplement | -0.2846 [-0.3657--0.2034] | <0.0001 | 1116.4 |
| 43 | Vitamin A-mothers | -0.5417 [-0.6642--0.4192] | <0.0001 | 1090.0 |

Consequently, 24 determinants were candidate variables in elastic net regression. By balancing between simplicity (least number of predictors) and accuracy via the *lambda.1se value* from the *glmnet* package [14,15], nine factors had a regression coefficient of zero were therefore excluded. They included household wealth index, continued breastfeeding, precipitation, contraceptive use, maternal education, access to improved water, rural residence, two doses of tetanus toxoid vaccination and underweight. Based on variance inflation fractions (VIF) of the remaining 15 factors, three supplements (iron and vitamin A for mother and child ) were multicollinear [16]. They were combined into a single index using PCA because they were introduced and scaled up around the same time and when combined, their effect on U5M would be interpretable (Table 3)

Table 3: Variance Inflation Factors summary of 15 factors. Three supplements (Vitamin A for mothers, for Iron mother and Vitamin A for children) were collinear and were combined into a supplements index using PCA

| **ID** | **Variable** | **VIF** |
| --- | --- | --- |
| 1 | Vitamin A-Mother | 23.76 |
|  | Iron mother | 15.7 |
|  | Vitamin A- Child | 4.33 |
| 2 | Health facility delivery | 3.88 |
| 3 | High parity | 3.83 |
| 4 | ANC4 | 3.49 |
| 5 | Fully immunized | 3.21 |
| 6 | Malaria | 2.81 |
| 7 | Better sanitation | 2.71 |
| 8 | Fever treatment-seeking | 2.68 |
| 9 | Diarrhoea treatment-seeking | 2.62 |
| 10 | HIV | 2.09 |
| 11 | Breastfed within one hour | 2.07 |
| 12 | Stunted | 2.07 |
| 13 | Female-headed households | 1.32 |

The default model of the remaining 13 factors had a DIC of -2452.83. However, the model excluding ANC4, treatment-seeking for diarrhoea and the supplements index had the lowest DIC (-2457.43) relative to any other possible combination of the 13 factors (Table 4). Consequently, ten factors; the proportion of health facility delivery, mothers with high parity, proportion of fully immunized children, malaria infection prevalence, households with access to better sanitation, proportion of children seeking treatment after fever, HIV infection prevalence, infants breast within the first hour of birth, proportion of stunted and the proportion of female-headed households were included a Bayesian ecological space-time mixed-effects regression model.

Table 4: Deviance information criterion (DIC) of various models.

| **ID** | **Factor** | **DIC** |
| --- | --- | --- |
| 1 | Malaria | -1711.02 |
| 2 | Malaria + Parity | -2043.07 |
| 3 | Malaria + Parity+ early breastfeeding | -2173.37 |
| 4 | Malaria + Parity+ early breastfeeding +Better sanitation | -2264.05 |
| 5 | Malaria + Parity+ early breastfeeding +Better sanitation + HIV | -2330.00 |
| 6 | Malaria + Parity+ early breastfeeding +Better sanitation +HIV + female head | -2405.77 |
| 7 | Malaria + Parity+ early breastfeeding +Better sanitation +HIV +female head + fever treatment seeking | -2434.54 |
| 8 | Malaria + Parity+ early breastfeeding +Better sanitation +HIV +female head + fever treatment seeking+ health facility births | -2455.22 |
| 9 | Malaria + Parity+ early breastfeeding +Better sanitation +HIV + female head + fever treatment seeking+ health facility births +Stunted | -2457.19 |
| 10 | Malaria + Parity+ early breastfeeding +Better sanitation +HIV + female head + fever treatment seeking+ health facility births +Stunted + fully immunized | -2457.43 |
| 11 | Model 10 +ANC4 | -2455.4 |
| 12 | Model 10 + diarrhoea treatment seeking | -2455.4 |
| 13 | Model 10 +ANC4+ diarrhoea treatment seeking | -2453.39 |
| 14 | Model 10 + supplements index | -2457.04 |
| 15 | Model 10 + supplements index+ANC4 | -2454.77 |
| 16 | Model 10 + supplements index+ diarrhoea treatment-seeking | -2455.03 |
| 17 | All 13 factors | -2452.8 |

The predictive capability/accuracy of the *base model 17* with 13 factors was compared against *model 10* with the least DIC and having ten factors using mean square error (MSE), root mean square error (RMSE), and the correlation between observed and predicted U5M values using a 15% hold out set. MSE is the mean of the squared differences indicating how close the estimator is to the true value and incorporates both bias and precision. On the other hand, RMSE is an accuracy measure equal to the square root of the MSE [17]. The best fitting model based on DIC with ten factors (*Model 10*) had a marginally better predictive accuracy with both the RSME and MSE being lower compared to the *base model 17* with all the 13 factors (Table 5)

Table 5: The Root Mean Square Error (RMSE), mean square error (MSE) and the correlation coefficient between observed and predicted U5M values for the default model with ten factors and the best fitting model with ten factors.

| **Indicator** | **13 factors - Model 17** | **10 factors - Model 10** |
| --- | --- | --- |
| RMSE | 0.071696 | 0.071298 |
| MSE | 0.005140 | 0.005083 |
| correlation coefficient | 0.986541 | 0.986695 |

**Section 2: Model convergence and accuracy assessment**

The stabilization and convergence of the model were explored using trace plots and Gelman-Rubin statistics while the accuracy was assessed via Monte Carlo error and standard deviation (SD) [18,19]. The trace plots history after running two chains simultaneously are shown in Figure 1 for several randomly picked parameters in blue (chain 1) and red (chain 2). The two chains appear to be overlapping, consequently, there is some reasonable confidence that convergence and or stabilization was achieved (Figure 1).

The Gelman-Rubin convergence statistics assessment of model convergence is shown in Figure 2 for 12 randomly picked parameters. Green shows the width of the central 80% interval of the pooled runs, blue shows the average width of the 80% intervals within the individual runs. The ratio between them *R* (= pooled / within) is indicated by red [18,19]. The R converges to 1 while the width of both the pooled and within interval widths are seen to converge to stability (Figure 2)

The accuracy of the posterior estimates was assessed using the MC error, the difference between the mean of the sampled values and the true posterior mean. The rule of thumb states that the simulation should be run until the MC error for each parameter is less than about 5% of the SD [18]. The ratio of MC error and SD from the summary statistics are shown in Table 1 and were all less than 5%.

Figure 1: Trace plots history of monitored parameters for exploring convergence. The red and blue colours represent chain 1 and 2 respectively run simultaneously.


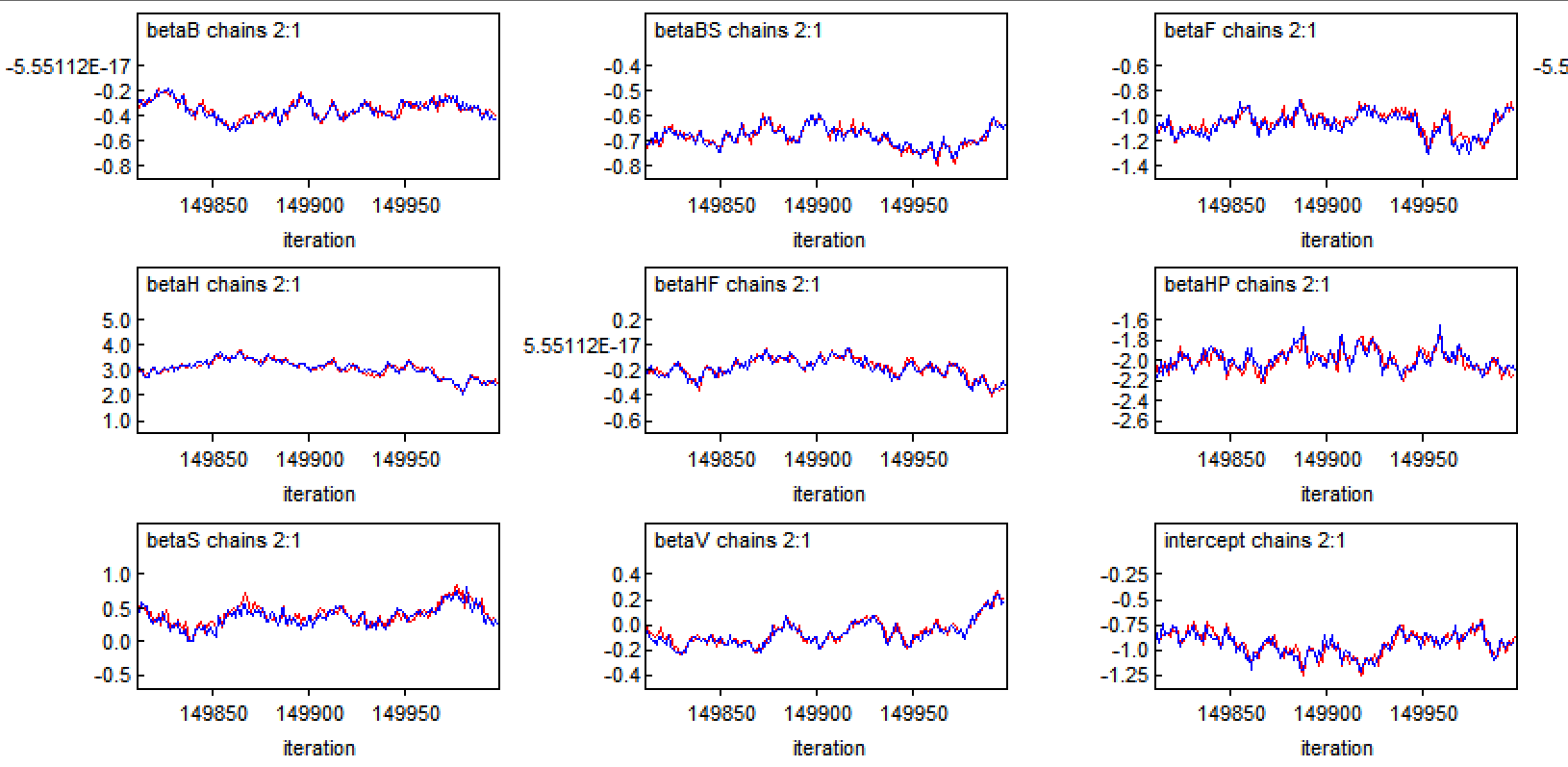


Figure 2: Plots of Gelman -Rubin statistics monitored parameters. Green is the width of the central 80% interval of the pooled runs, blue the average width of the 80% intervals within the individual runs and their ratio indicated by red.


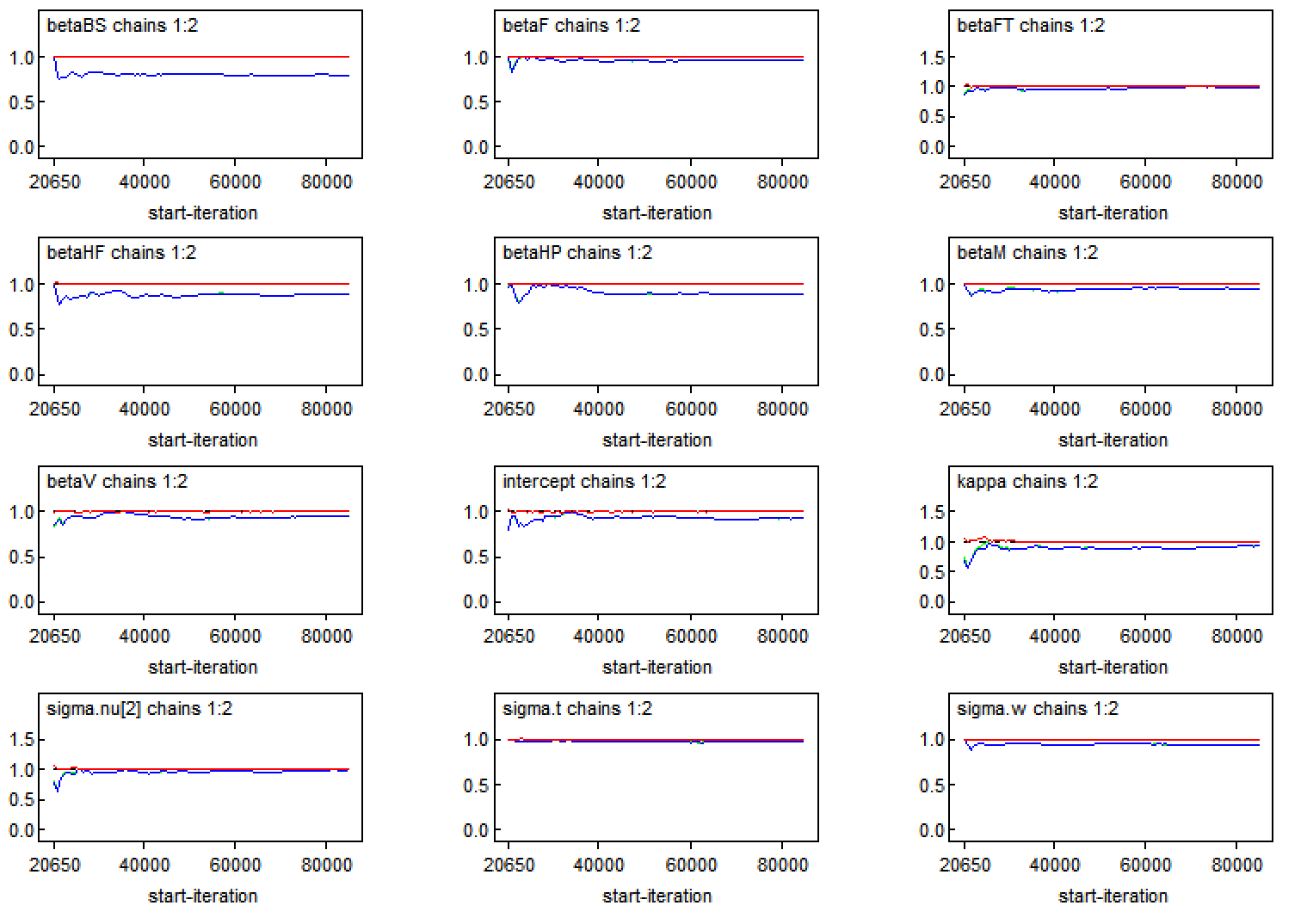


Table 6: The SD, MC error and their a ratio from a total of 130,000 iterations and a burn of 20,000 for the intercept, fixed effects, spatial and temporal random effects and their interaction

| **Determinant** | **SD** | **MC error** | **Ratio** |
| --- | --- | --- | --- |
| Breastfed within one hour | 0.06628 | 0.00085 | 1.3% |
| Better Sanitation | 0.05935 | 0.00112 | 1.9% |
| Female headed household | 0.09821 | 0.00124 | 1.3% |
| Fever treatment seeking | 0.11080 | 0.00127 | 1.2% |
| HIV risk | 0.43160 | 0.00894 | 2.1 % |
| Health Facility Delivery | 0.08057 | 0.00138 | 1.7% |
| High Parity | 0.13310 | 0.00186 | 1.4% |
| Malaria risk | 0.03936 | 0.00039 | 1.0% |
| Stunted | 0.17660 | 0.00279 | 1.6% |
| Fully immunized | 0.07179 | 0.00087 | 1.2% |
| Intercept | 0.13650 | 0.00234 | 1.7% |
| sigma.nu[1] | 0.00434 | 0.00015 | 3.4% |
| sigma.nu[2] | 0.01267 | 0.00025 | 2.0% |
| sigma.t | 0.00903 | 0.00004 | 0.4% |
| sigma.w | 0.06225 | 0.00028 | 0.5% |

# References

1 Okiro EA. Estimates of subnational health trends in Kenya. *Lancet Glob Heal* 2019;**7**:e8–9. doi:10.1016/S2214-109X(18)30516-3

2 Moraga P, Cano J, Baggaley RF, *et al.* Modelling the distribution and transmission intensity of lymphatic filariasis in sub-Saharan Africa prior to scaling up interventions: Integrated use of geostatistical and mathematical modelling. *Parasites and Vectors* 2015;**8**:1–16. doi:10.1186/s13071-015-1166-x

3 Deribe K, Fronterre C, Dejene T, *et al.* Measuring the spatial heterogeneity on the reduction of vaginal fistula burden in Ethiopia between 2005 and 2016. *Sci Rep* 2020;**10**:972. doi:10.1038/s41598-020-58036-0

4 Noor AM, Kinyoki DK, Mundia CW, *et al.* The changing risk of Plasmodium falciparum malaria infection in Africa: 2000-10: A spatial and temporal analysis of transmission intensity. *Lancet* 2014;**383**:1739–47. doi:10.1016/S0140-6736(13)62566-0

5 Snow RW, Kibuchi E, Karuri SW, *et al.* Changing malaria prevalence on the Kenyan coast since 1974: Climate, drugs and vector control. *PLoS One* 2015;**10**:e0128792. doi:10.1371/journal.pone.0128792

6 Macharia PM, Giorgi E, Noor AM, *et al.* Spatio-temporal analysis of Plasmodium falciparum prevalence to understand the past and chart the future of malaria control in Kenya. *Malar J* 2018;**17**:340. doi:10.1186/s12936-018-2489-9

7 Gething PW, Casey DC, Weiss DJ, *et al.* Mapping *Plasmodium falciparum* Mortality in Africa between 1990 and 2015. *N Engl J Med* 2016;**375**:2435–45. doi:10.1056/NEJMoa1606701

8 GoK MoH. Kenya National Malaria policy. 2010.www.nmcp.or.ke (accessed 22 May 2016).

9 Brabin BJ. An analysis of malaria in pregnancy in Africa. *Bull World Health Organ* 1983;**61**:1005–16.

10 Brabin BJ. The risks and severity of malaria in pregnant women. 1991;**1**:1–33.http://apps.who.int/iris/bitstream/10665/61511/1/TDR_FIELDMAL_1.pdf (accessed 18 Oct 2016).

11 Guyatt HL, Snow RW. The epidemiology and burden of Plasmodium Falciparum-related anemia among pregnant women in sub-Saharan Africa. *Am J Trop Med Hyg* 2001;**64**:36–44.

12 Guyatt HL, Snow RWR. Impact of malaria during pregnancy on low birth weight in sub-Saharan Africa. *Clin Microbiol Rev* 2004;**17**:760–9. doi:10.1128/CMR.17.4.760

13 Macharia PM, Joseph NK, Sartorius B, *et al.* Subnational estimates of factors associated with under-five mortality in Kenya: a spatio-temporal analysis, 1993-2014. *Submitted* 2020.

14 Waldmann P, Mészáros G, Gredler B, *et al.* Evaluation of the lasso and the elastic net in genome-wide association studies. *Front Genet* 2013;**4**:1–11. doi:10.3389/fgene.2013.00270

15 Friedman J, Hastie T, Tibshirani R. Regularization paths for generalized linear models via coordinate descent. *J Stat Softw* 2010;**33**:1–22. doi:10.18637/jss.v033.i01

16 Pezzulo C, Bird T, Utazi EC, *et al.* Geospatial Modeling of Child Mortality across 27 Countries in Sub- Saharan Africa. DHS Spat. Anal. Reports No. 13. 2016.http://dhsprogram.com/pubs/pdf/SAR13/SAR13.pdf (accessed 1 Jan 2016).

17 Walther BA, Moore JL, Rahbek C. The concepts a literature with of species richness the performance estimators , of estimator review performance precision. *Ecography (Cop)* 2005;**28**:815–29.

18 Spiegelhalter D, Thomas A, Best N, *et al.* WinBUGS User Manual Version 1.4 MRC Biostatistics Unit. 2003.http://citeseerx.ist.psu.edu/viewdoc/download?doi=10.1.1.726.775&rep=rep1&type=pdf (accessed 5 Jan 2019).

19 Gelman A, Rubin DB. Inference from iterative simulation using multiple sequences. *Stat Sci* 1992;**7**:457–72. doi:10.1214/ss/1177011136
