# Supplementary material for: The impact of child health interventions and risk factors on child survival in Kenya, 1993–2014: a Bayesian spatio-temporal analysis with counterfactual scenarios
Source: BMC Med. 2021 May 4;19:102. doi: 10.1186/s12916-021-01974-x (PMC8094495; doi:10.1186/s12916-021-01974-x)

**Additional file 3:**

**Section 1: Number of deaths averted per year [1994-2014] per county associated with changes in ten key factors relative to the baseline (1993)**

Figure 1: Number of deaths averted (green shades) and lives lost (yellow to brown) per year [1994-2014] per county if access to better sanitation relative to the baseline (1993) had remained unchanged.


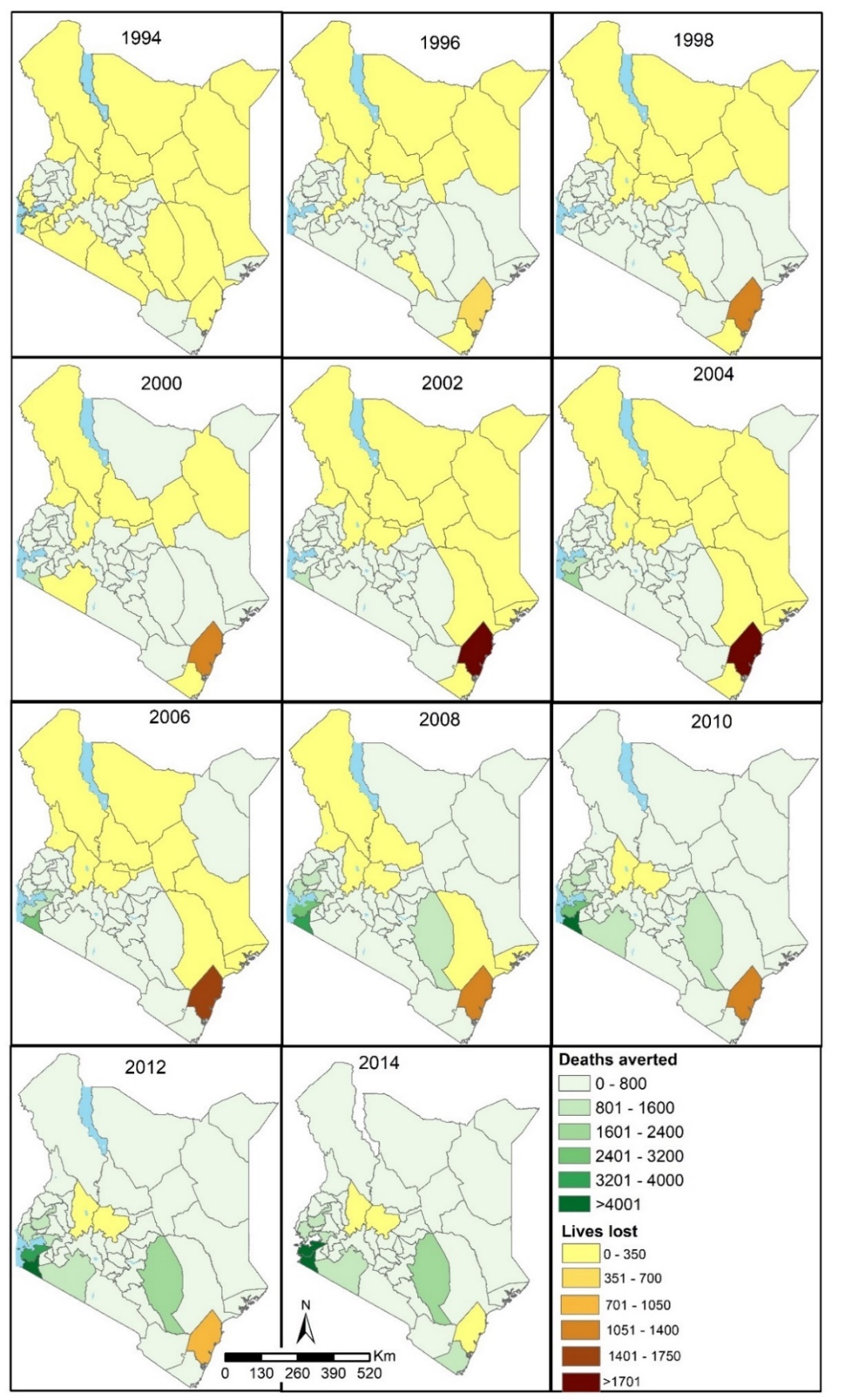


Figure 2: Number of deaths averted (green shades) and lives lost (yellow to brown) per year [1994-2014] per county if fever treatment-seeking rates relative to the baseline (1993) had remained unchanged.


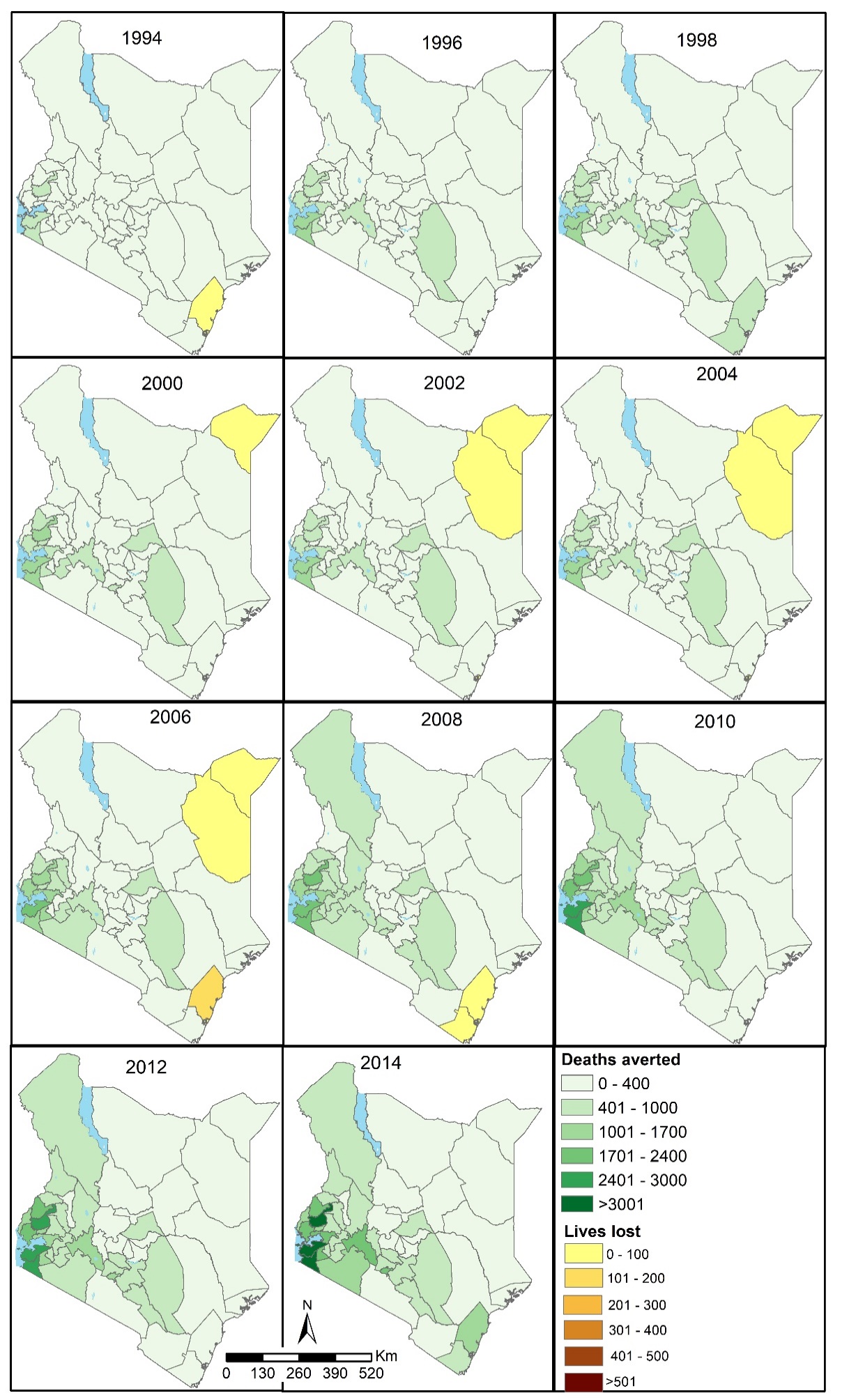


Figure 3: Number of deaths averted (green shades) and lives lost (yellow to brown) over 22 years [1993-2014] per county if malaria infection prevalence relative to the baseline (1993) had remained unchanged.


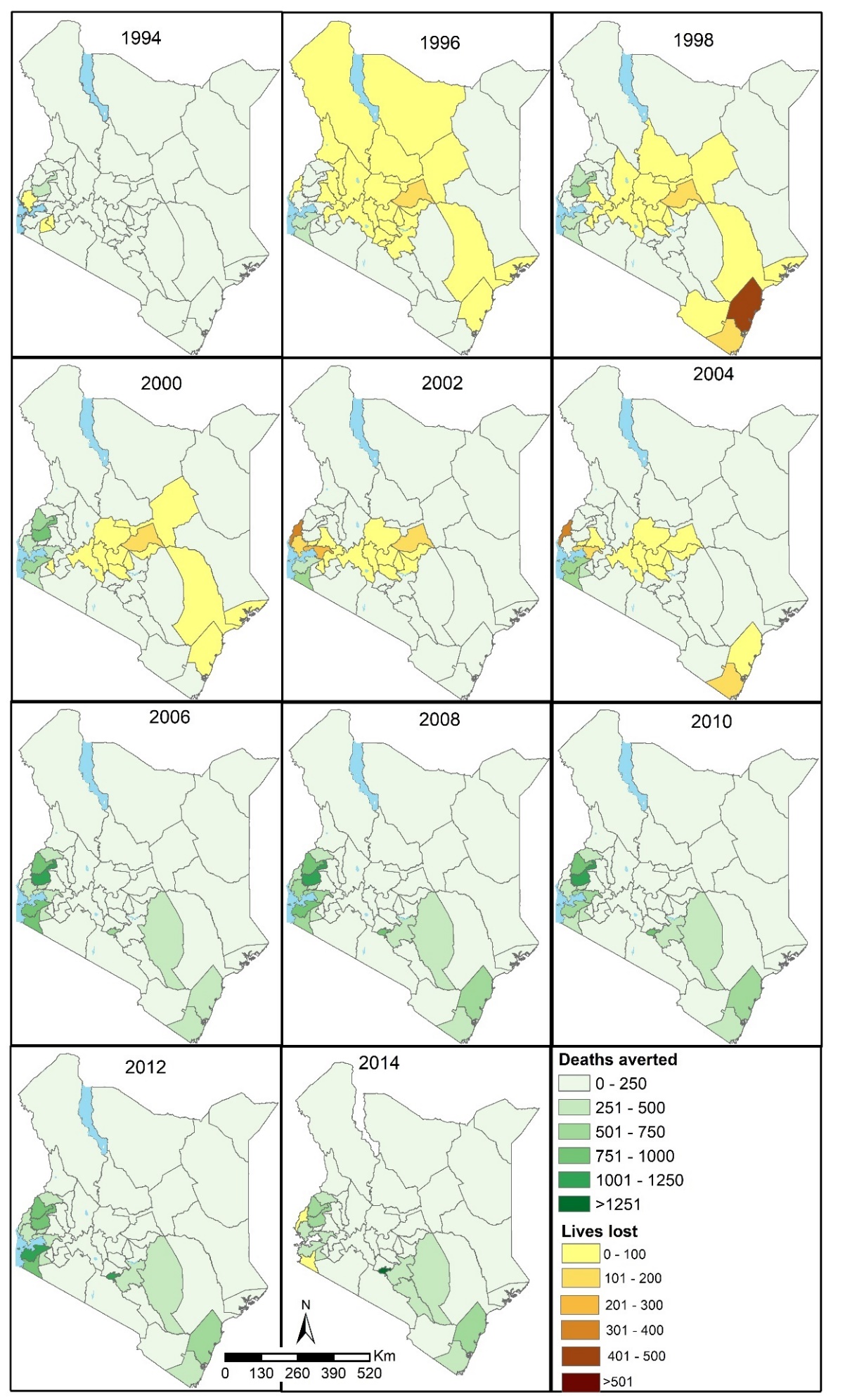


Figure 4: Number of deaths averted (green shades) and lives lost (yellow to brown) over 22 years [1993-2014] per county if the coverage of early breastfeeding relative to the baseline (1993) had remained unchanged.


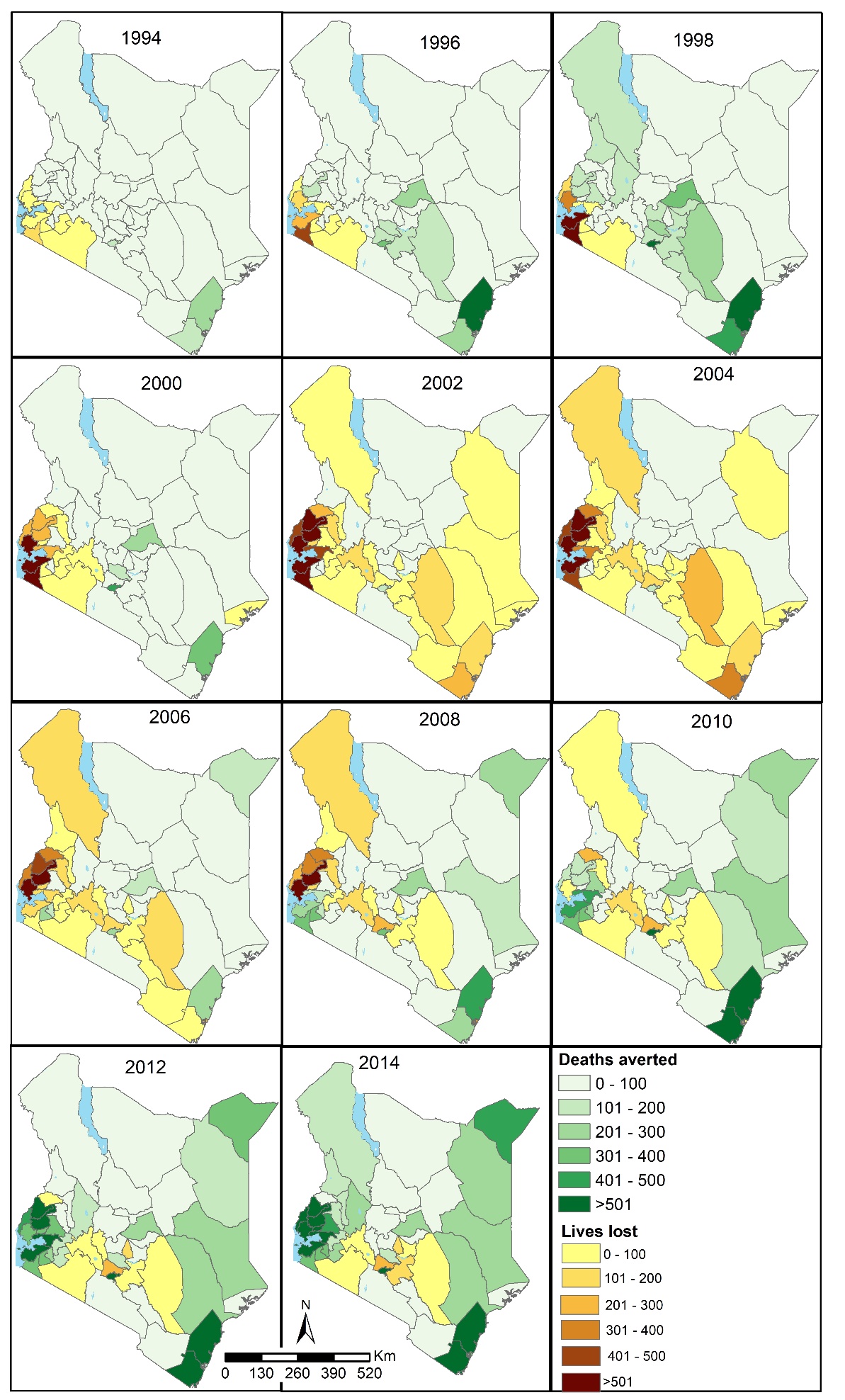


Figure 5: Number of deaths averted (green shades) and lives lost (yellow to brown) over 22 years [1993-2014] per county if the prevalence of stunting among children relative to the baseline (1993) had remained unchanged.


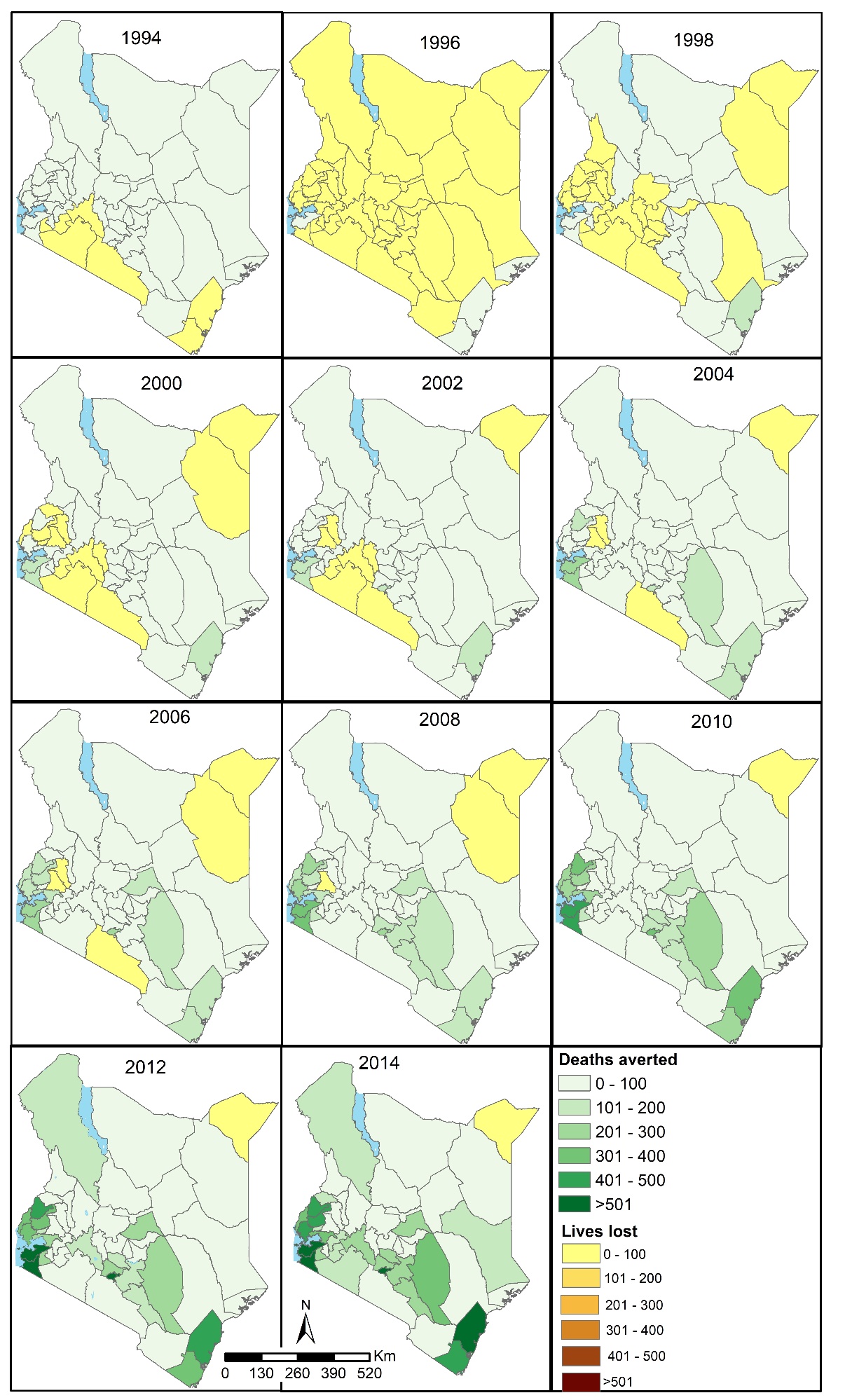


Figure 6: Number of deaths averted (green shades) and lives lost (yellow to brown) over 22 years [1993-2014] per county (if the coverage of maternal autonomy relative to the baseline (1993) had remained unchanged.


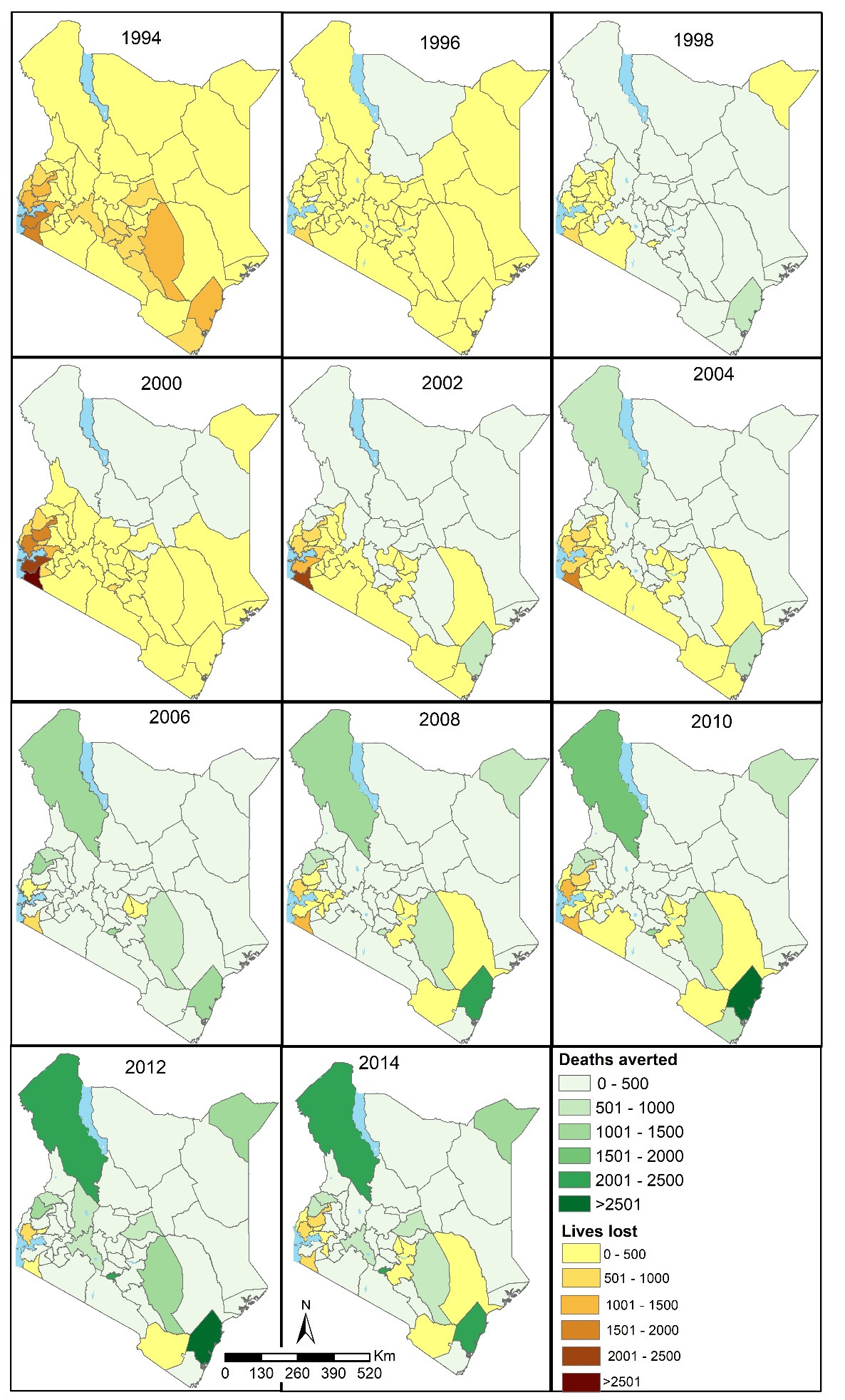


Figure 7: Number of deaths averted (green shades) and lives lost (yellow to brown) over 22 years [1993-2014] per county if the coverage of health facility deliveries relative to the baseline (1993) had remained unchanged.


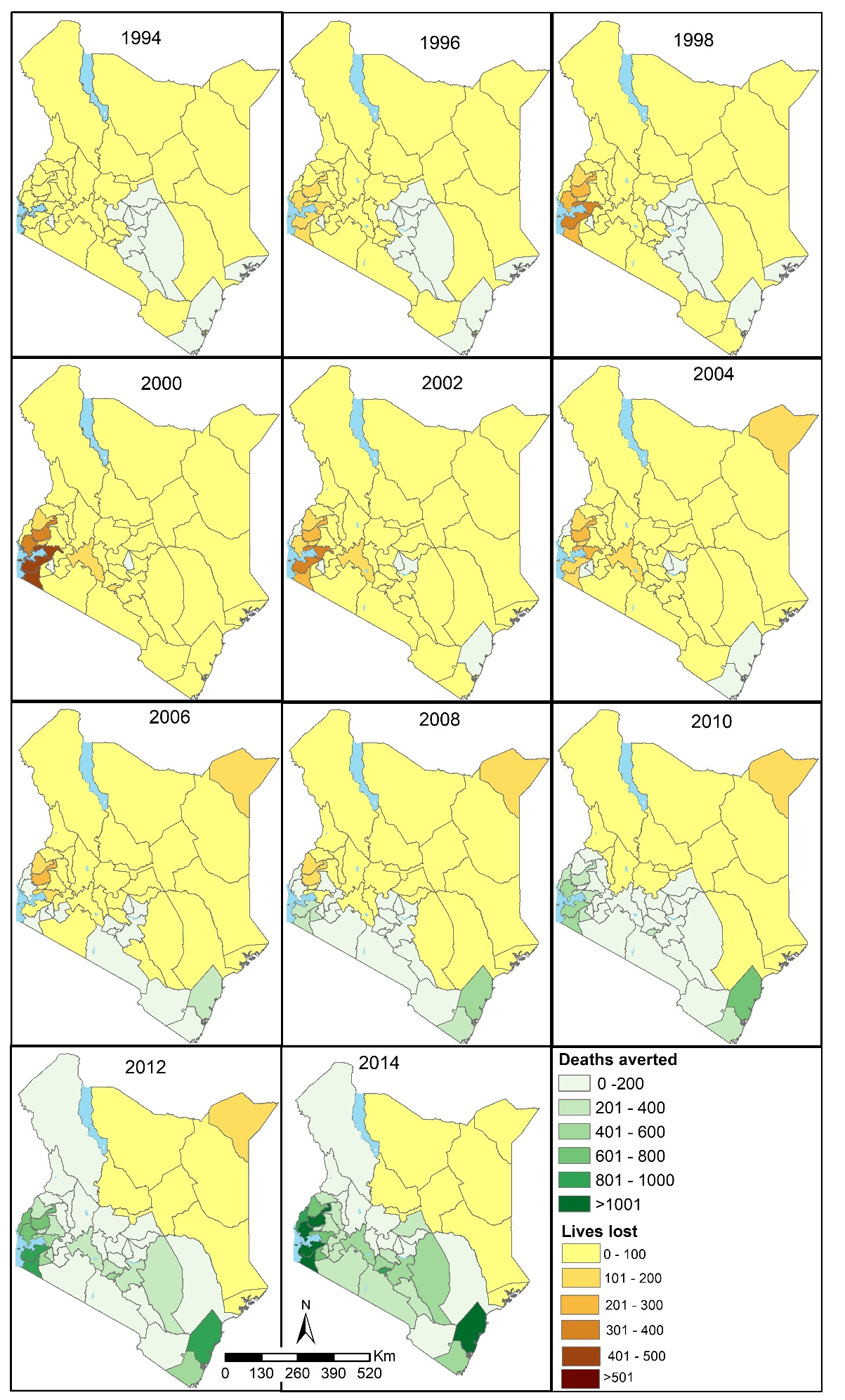


Figure 8: Number of deaths averted (green shades) and lives lost (yellow to brown) over 22 years [1993-2014] per county if the coverage of fully immunized children relative to the baseline (1993) had remained unchanged.


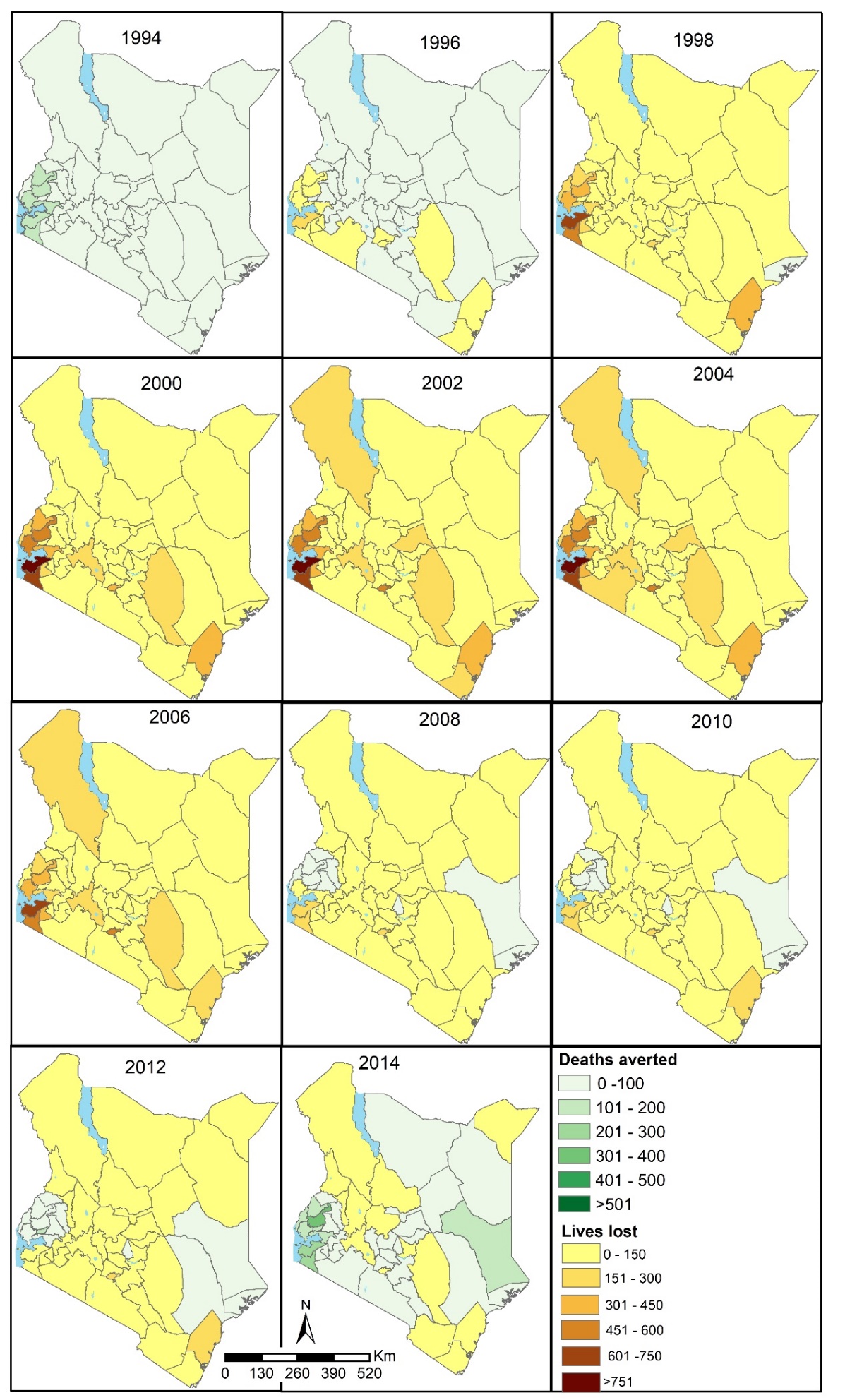


Figure 9: Number of deaths averted (green shades) and lives lost (yellow to brown) over 22 years [1993-2014] per county if the prevalence of high parity relative to baseline (1993) had remained unchanged.


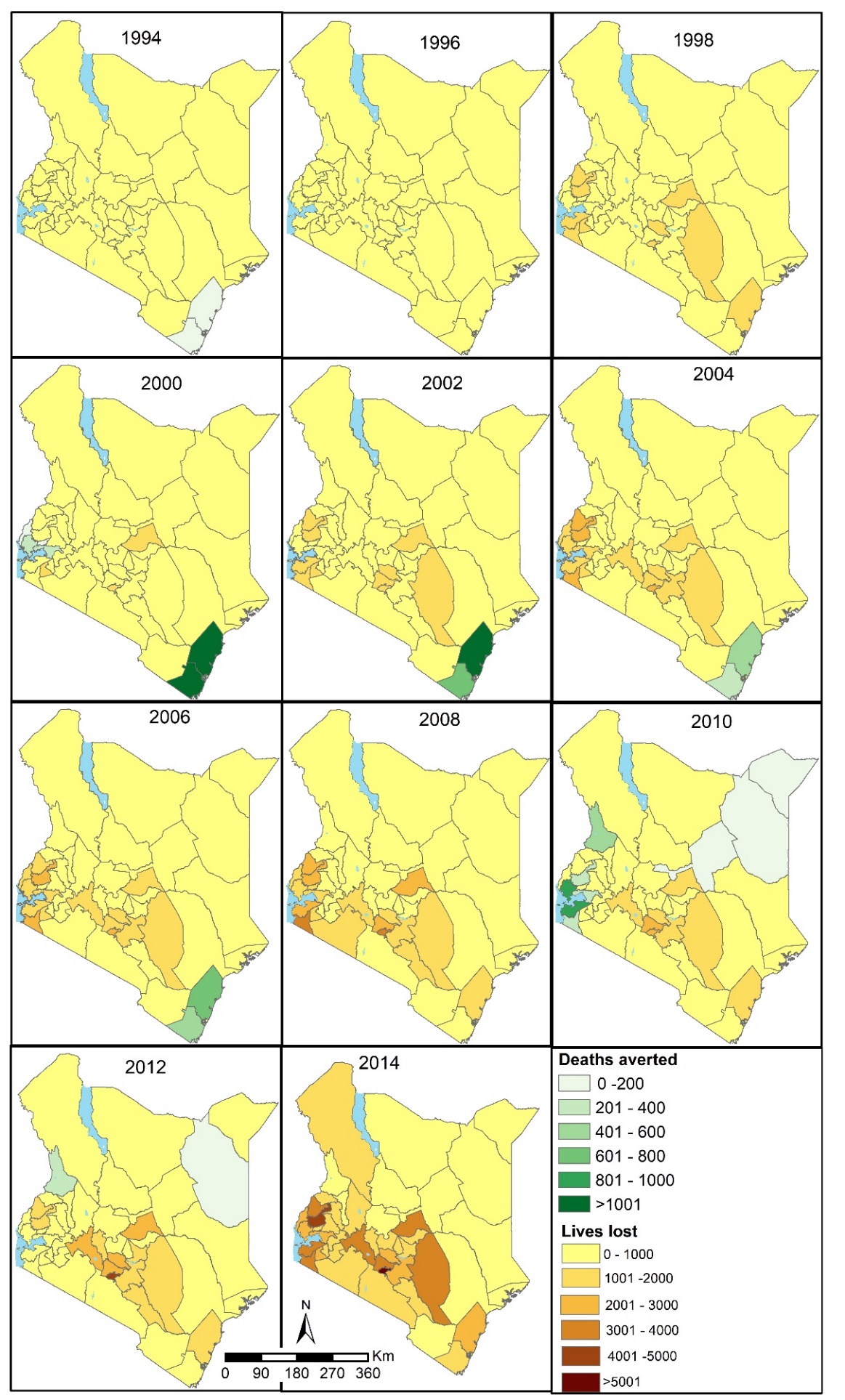


**Section 1:** A comparison of the annual number of under-five deaths averted (U5-DA) or lives lost (U5-LL) between 1993 and 2014 at the county. The magnitude of each colour represents the U5-DA (above zero-line) or U5-LL (below zero-line) per 1000 live births if the coverage/prevalence of the factors relative to 1993 had remained unchanged.


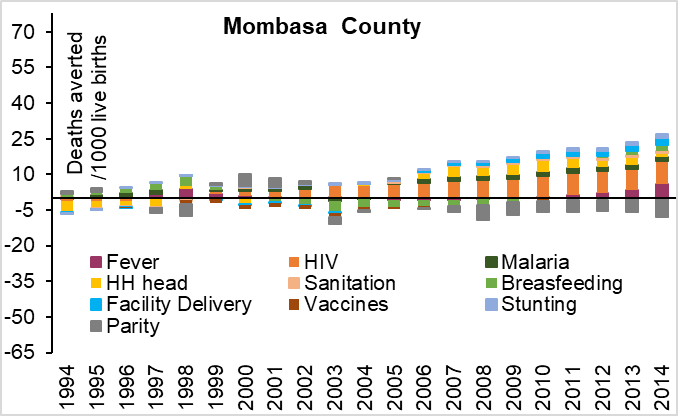

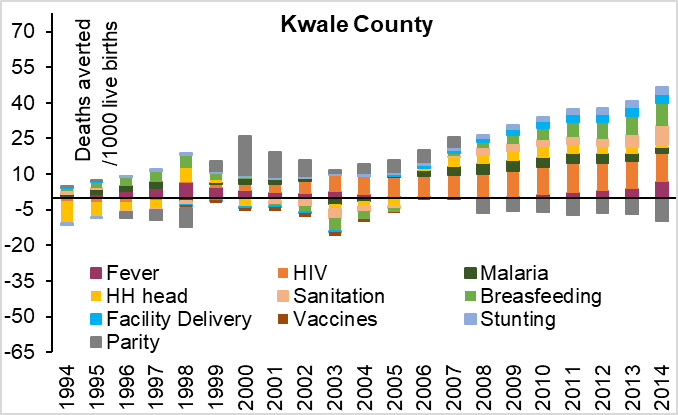


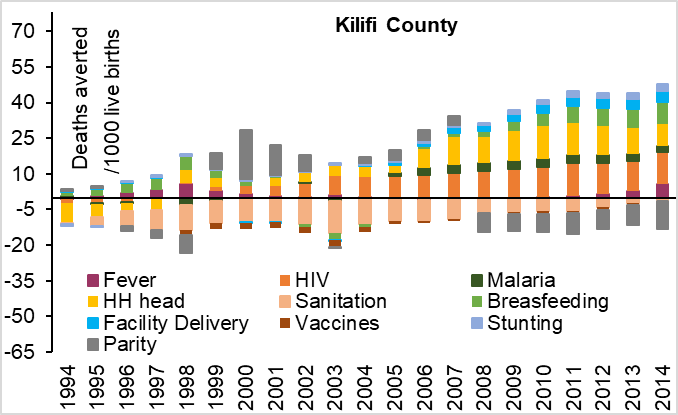

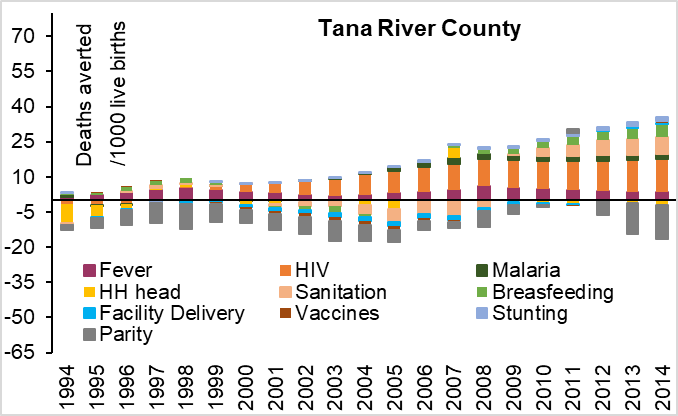


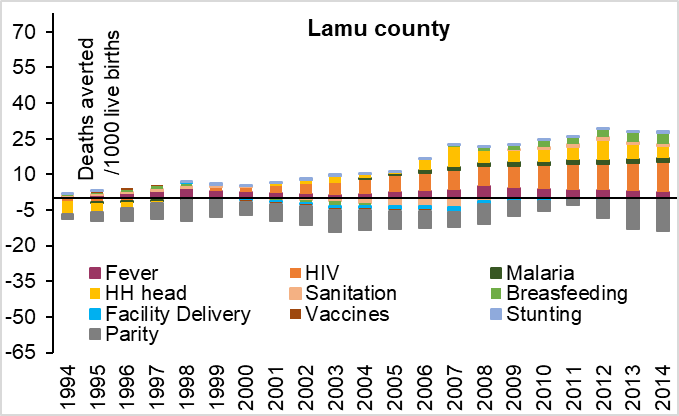

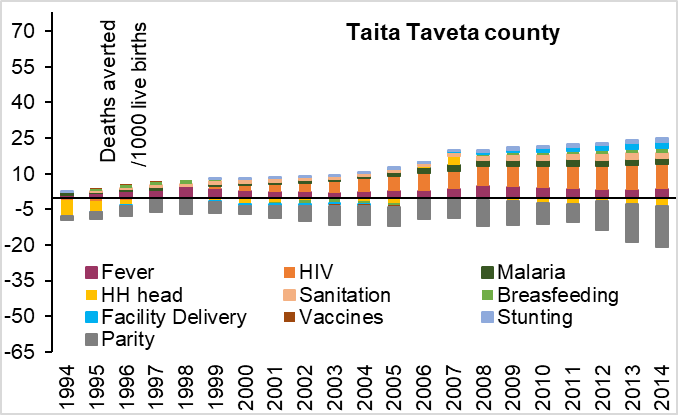


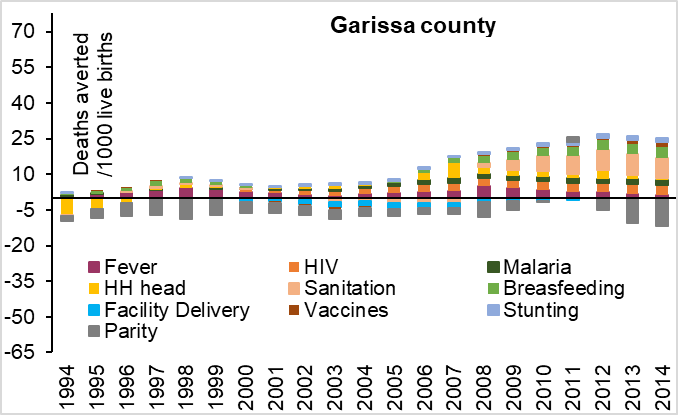

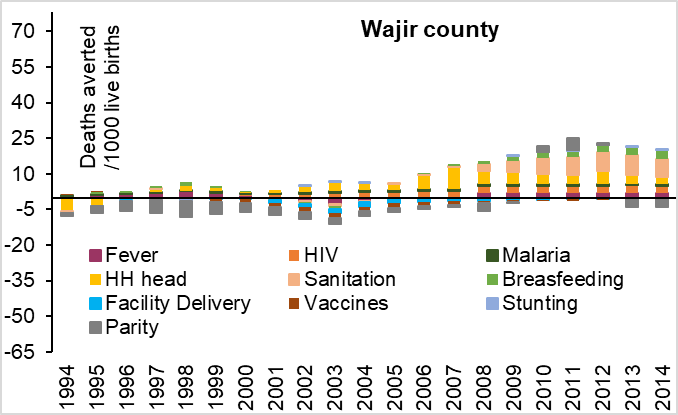


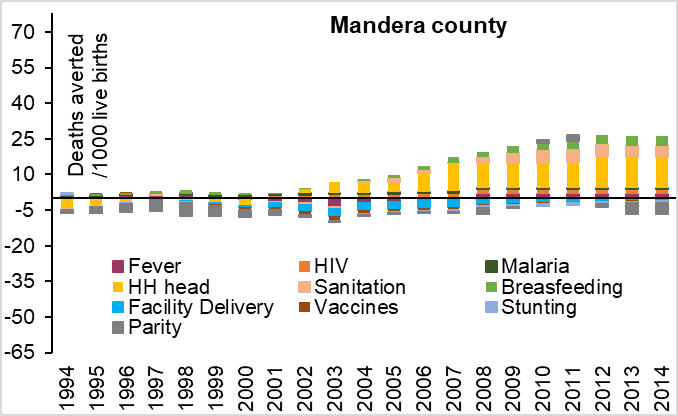

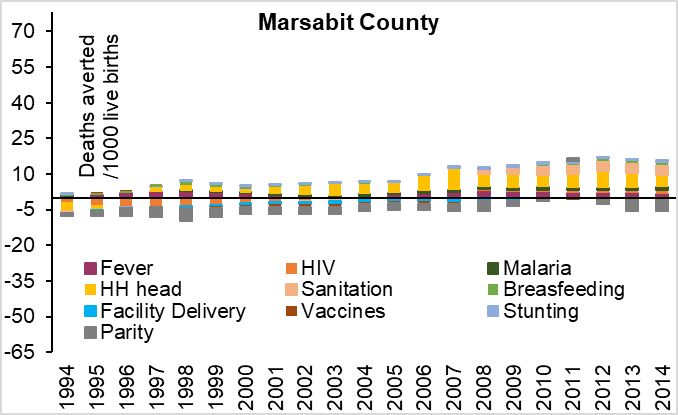


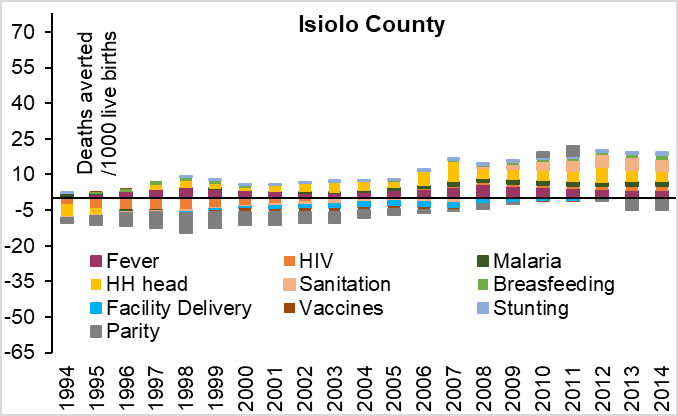

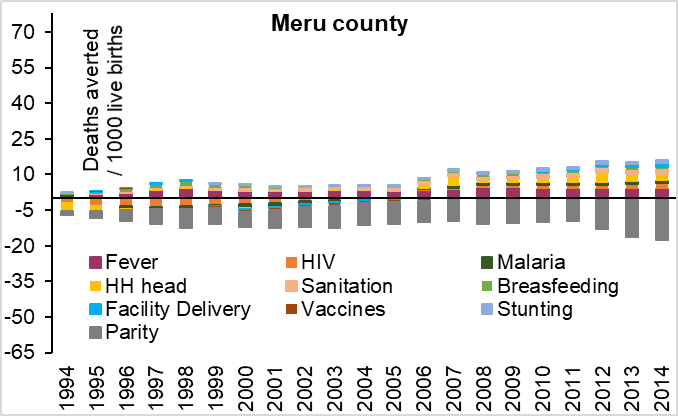


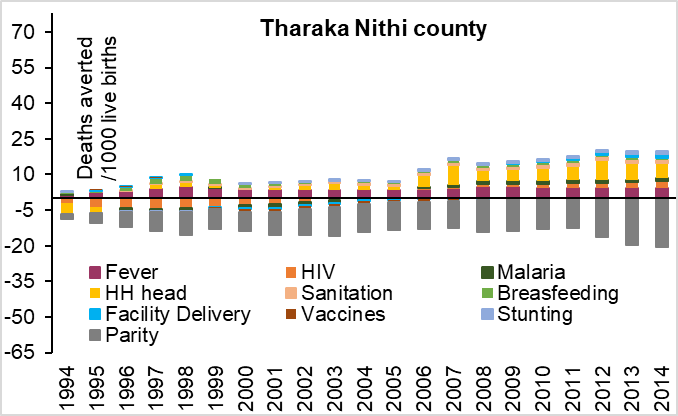

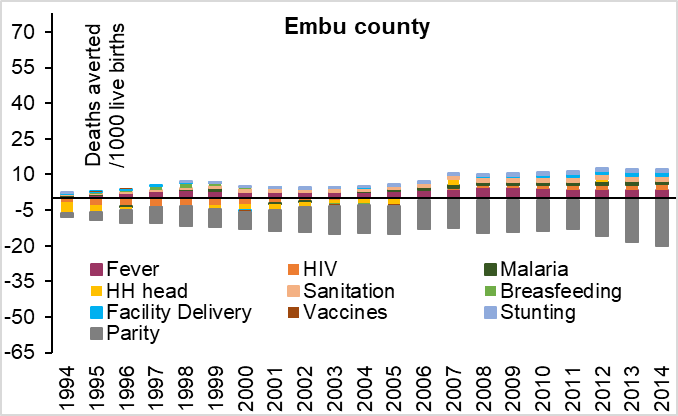


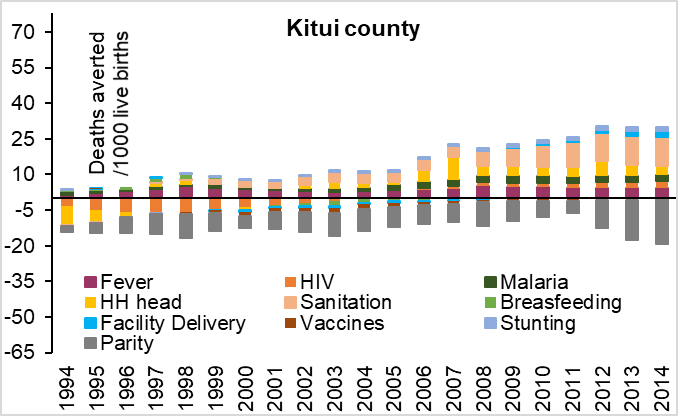

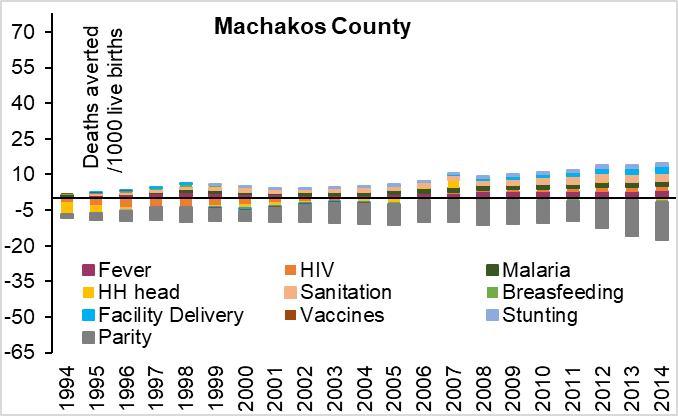


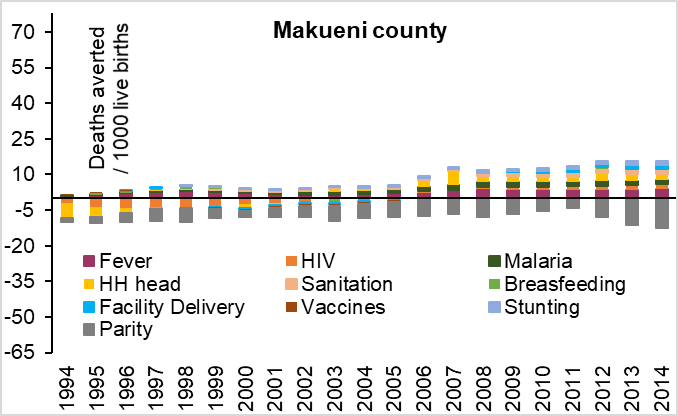

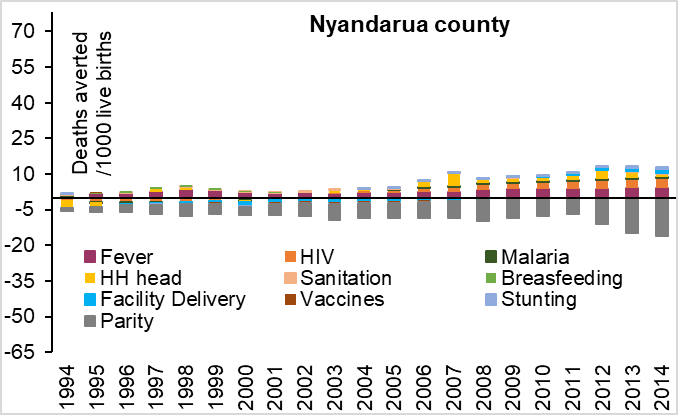


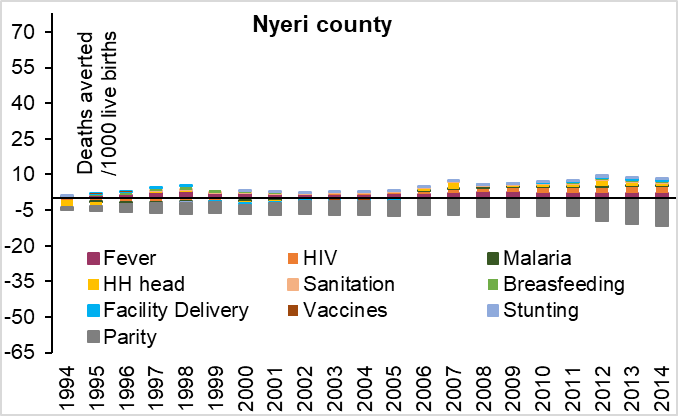

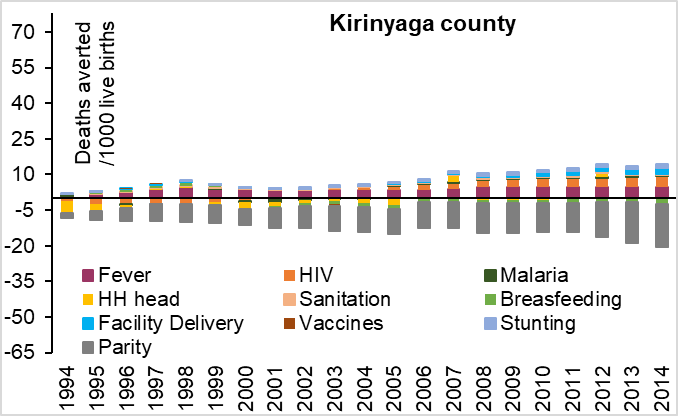


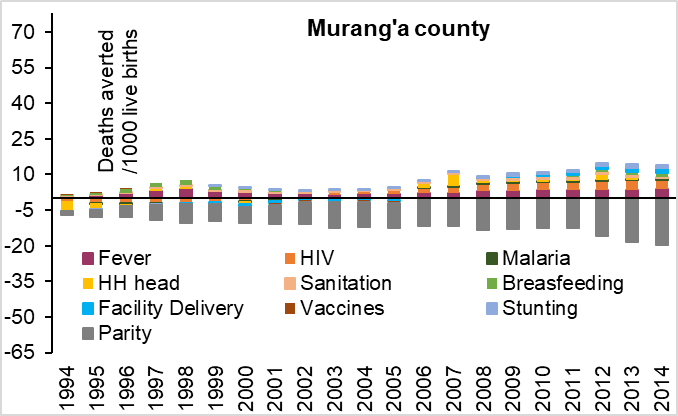

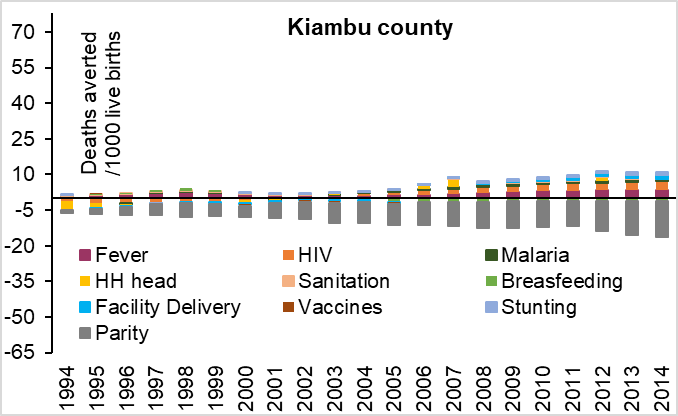


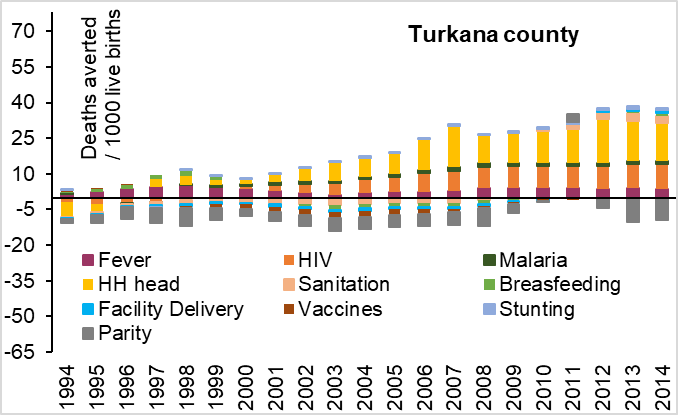

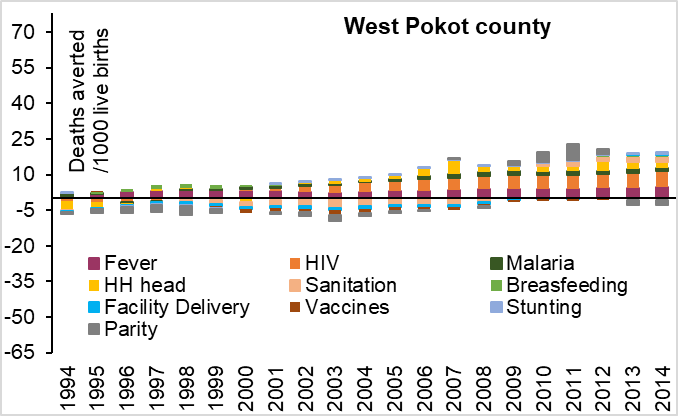


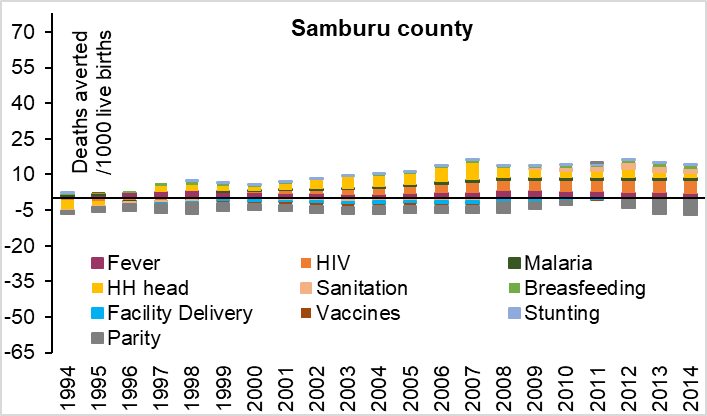

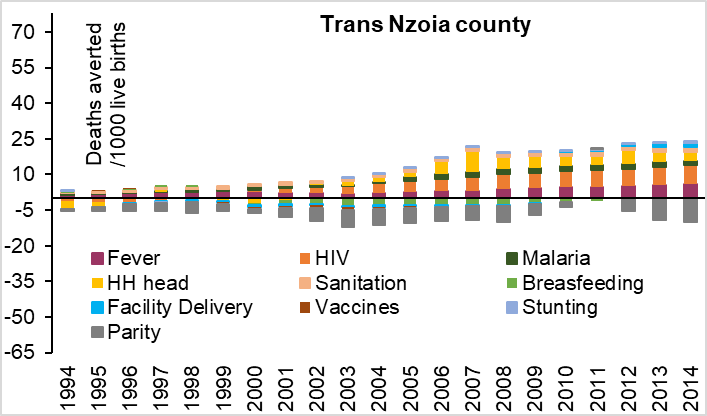


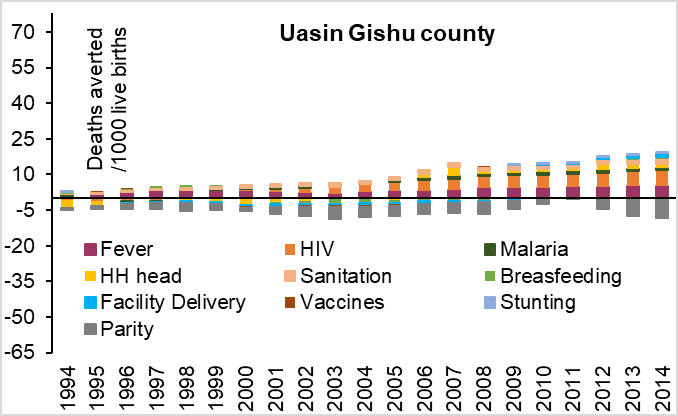

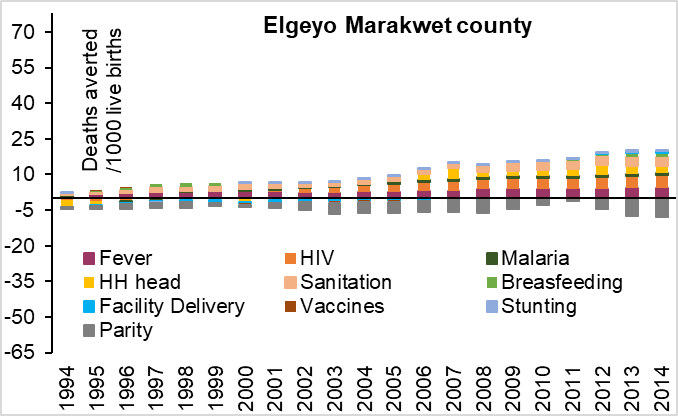


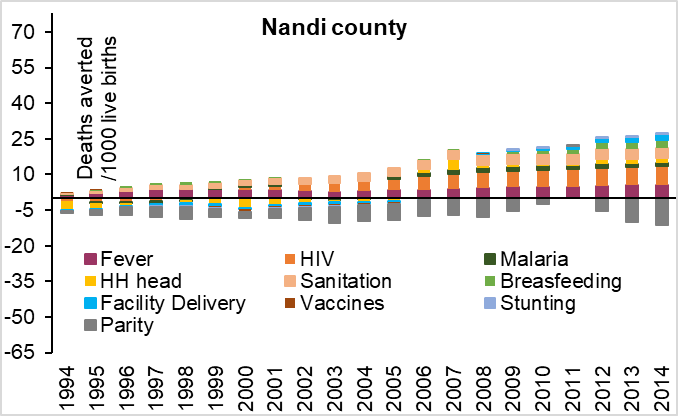

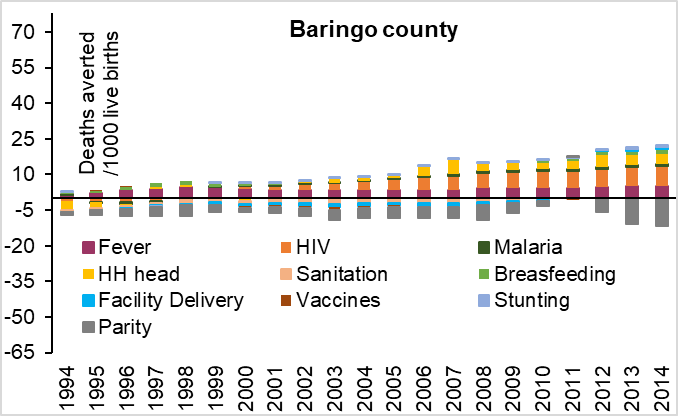

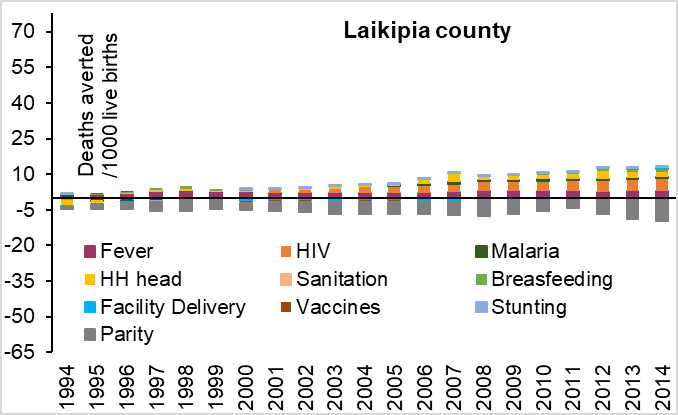

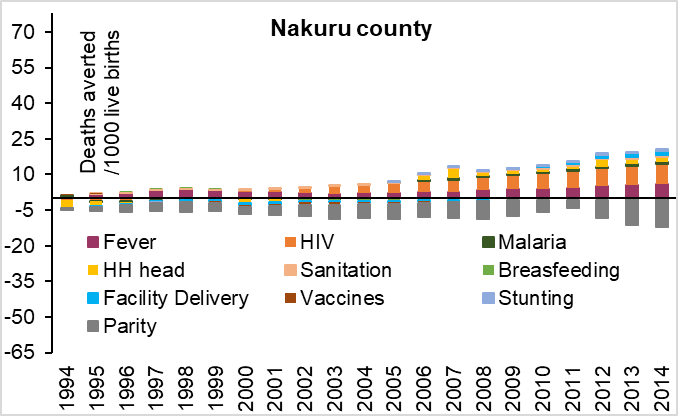

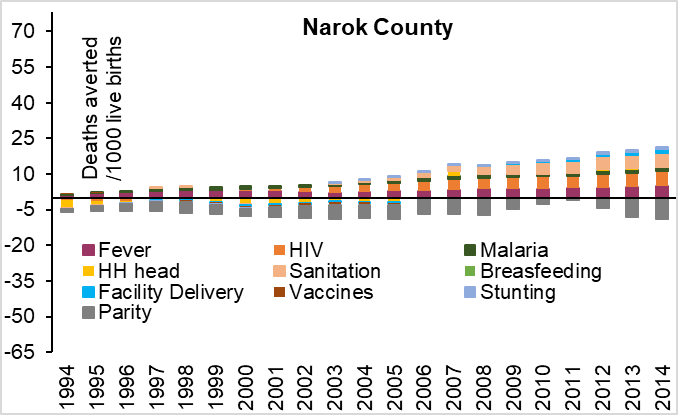

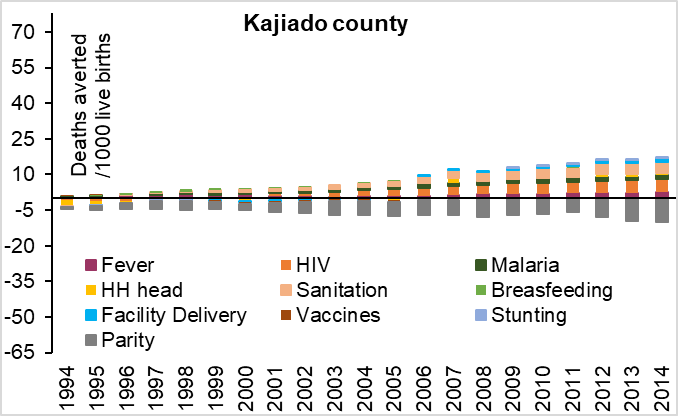

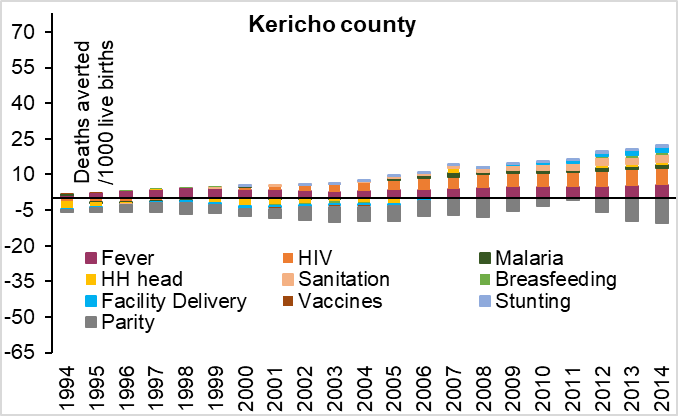

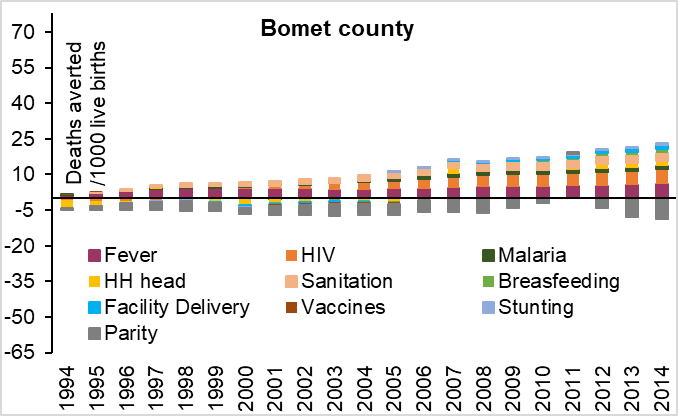

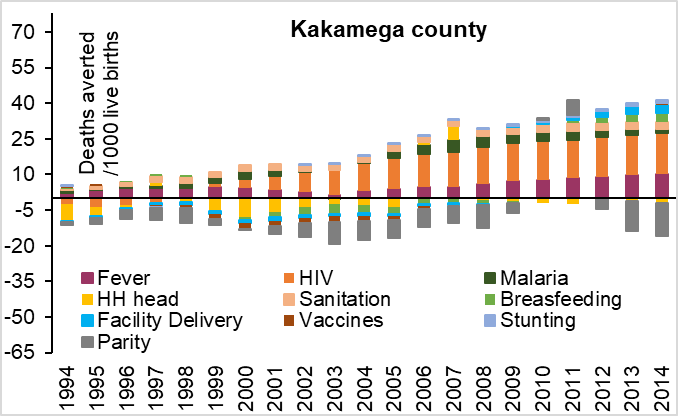

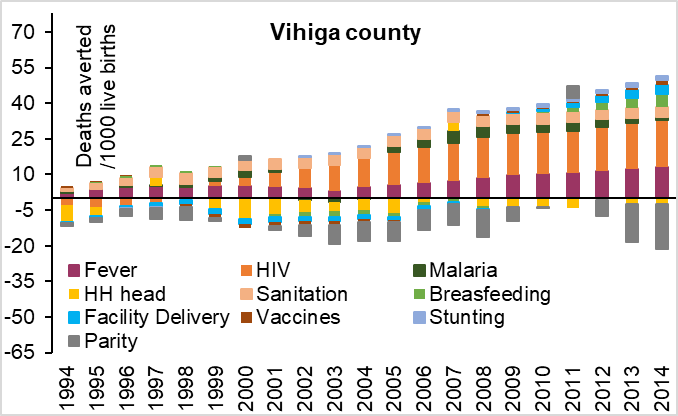

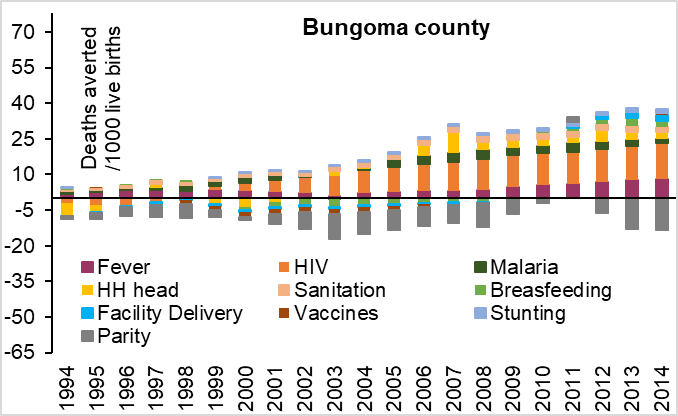

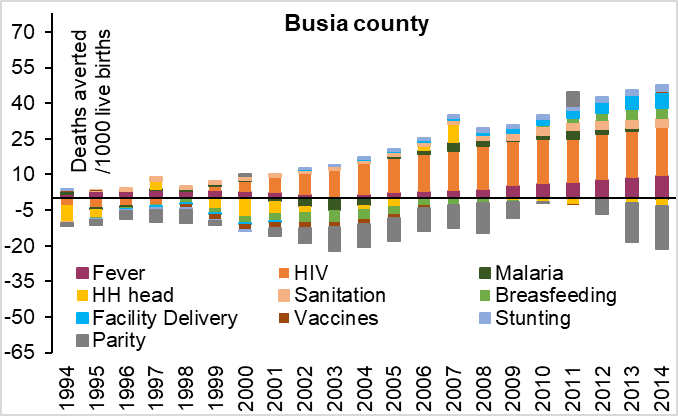

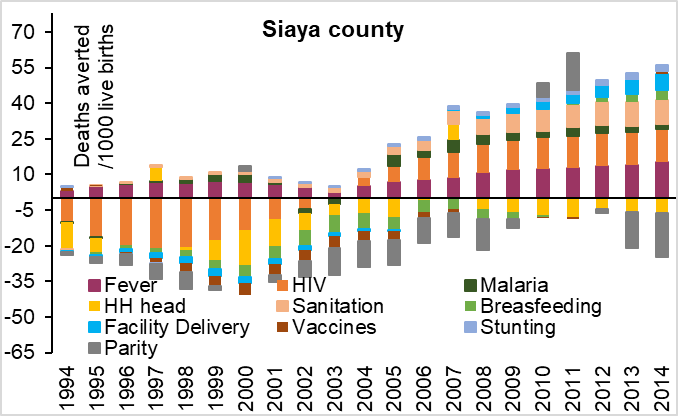

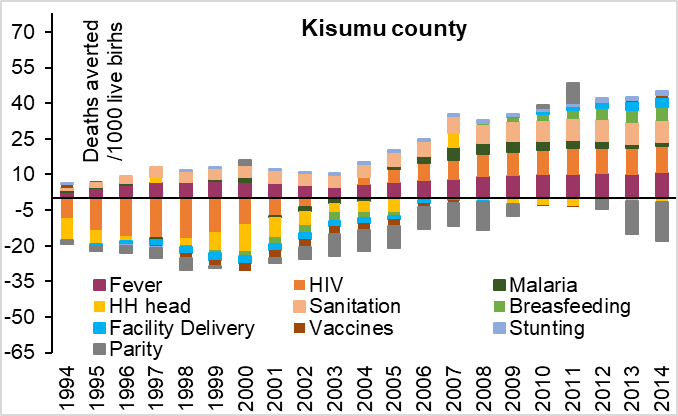

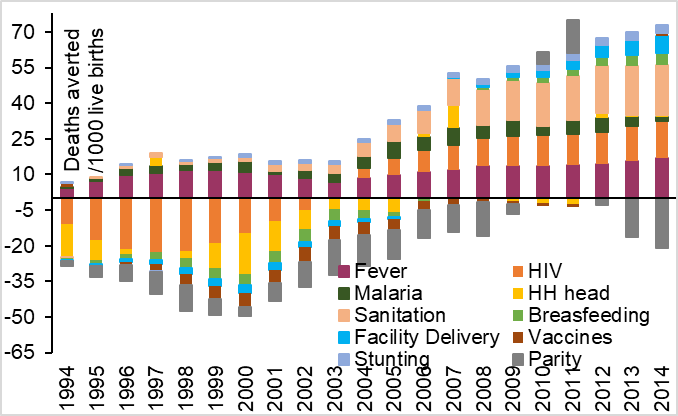

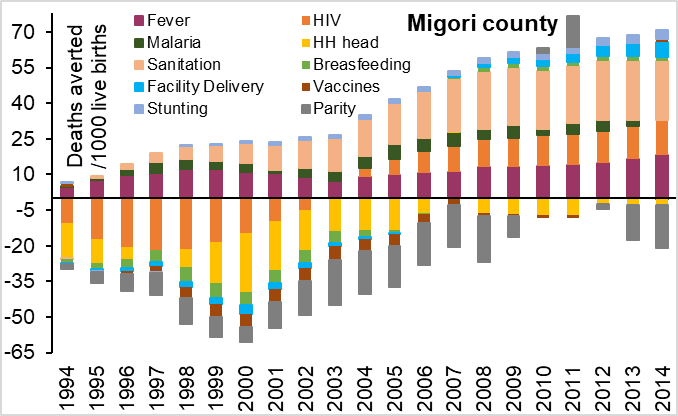


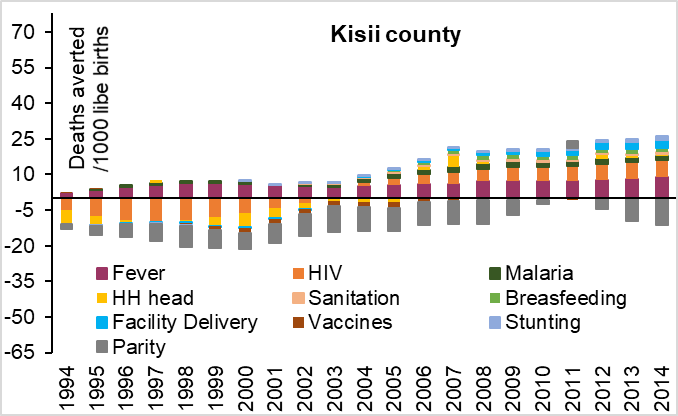

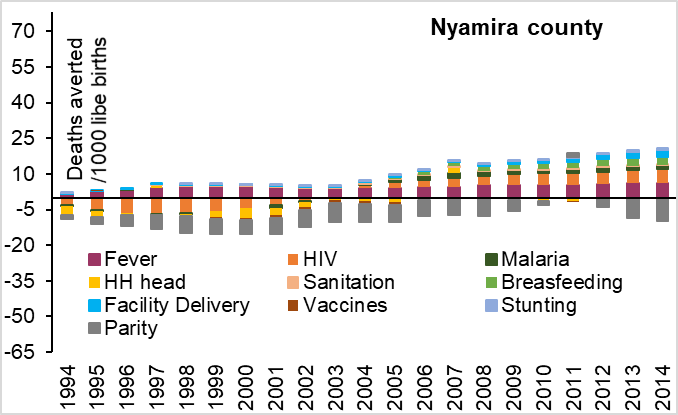

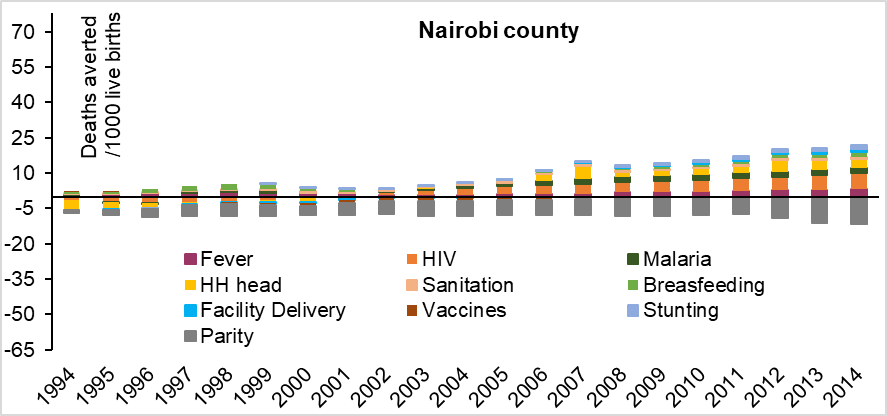

Supplement: Supplementary file 3 — Additional file 3. Annual number of deaths averted, and lives lost (1994–2014) per county if coverage/ prevalence relative to 1993 had remained unchanged for factors associated with child survival (Section 1) and their comparison via an overlay of all ten factors per county (Section 2). [file 12916_2021_1974_MOESM3_ESM.docx]
